# Supplementary material for: Recognizing and Responding to Overt Racism Towards Medical Trainees: Using the IRES Tool and Scripted Language
Source: MedEdPORTAL. 2024 Oct 24;20:11453. doi: 10.15766/mep_2374-8265.11453 (PMC11500618; doi:10.15766/mep_2374-8265.11453)
Supplement: Supplementary file 1 — Facilitator Guide.docxSlide Deck.pptxPractice Cases.docxIRES Tool.docxScripted Language.docxPostworkshop Evaluation.docx [file mep_2374-8265.11453-s001.zip › C. Practice Cases.docx]

Appendix C. Practice Cases

This handout provides the two case scenarios for participants to practice using the IRES Tool (Appendix D) and scripted language (Appendix E) with a partner in a breakout session. Use Scenario 1 for the first breakout session which will then be followed by a large group debrief. Use Scenario 2 for the second breakout session which will then be followed by a large group debrief. Each breakout session should be about 10 minutes followed by 10-15 minute large group facilitated debriefs.

| Case Based Practice  We do not intend to retraumatize individuals with this exercise. No one is playing the offending patient in this encounter. Both individuals in your small group will practice responding to this scenario. The focus of this exercise is to practice the language of responding to racial discrimination and supporting the learner. |
| --- |
| Scenario 1  You are the attending physician rounding with residents and third-year medical students. You are examining the patient, Ms. F, and ask if it is ok for the medical student to also examine her. She refuses, comments on the student’s appearance, then says a racial slur.   - - Take turns practicing responding to this incident.     - Go through the IRES tool     - Use the Scripted Language and say phrases out loud to your partner. Try out different phrases and note if any feel more natural to you.   - Then take turns practice checking in with your learner and team. |
| Scenario 2  You are the supervising physician in resident continuity clinic. One of your PGY-1 residents is asked by the patient to leave the room. The patient stated to the resident, “I can’t understand you. I only want to speak to a doctor who speaks English.”   - Take turns practicing responding to this incident.   - - Go through the IRES Tool     - Use the Scripted Language and say phrases out loud to your partner. Try out different phrases and note if any feel more natural to you. - Take turns practicing checking in with your learner and team.   - - Use the Scripted Language and say phrases out loud to your partner. |
