# Supplementary material for: Recognizing and Responding to Overt Racism Towards Medical Trainees: Using the IRES Tool and Scripted Language
Source: MedEdPORTAL. 2024 Oct 24;20:11453. doi: 10.15766/mep_2374-8265.11453 (PMC11500618; doi:10.15766/mep_2374-8265.11453)
Supplement: Supplementary file 1 — Facilitator Guide.docxSlide Deck.pptxPractice Cases.docxIRES Tool.docxScripted Language.docxPostworkshop Evaluation.docx [file mep_2374-8265.11453-s001.zip › D. IRES Tool.docx]

Appendix D. The IRES (Identify, Respond, End, Support) Tool

Use this handout in the break out sessions. Read each case scenario and practice applying each part of this framework to the case out loud with your partner.

# Responding to Racism in the Learning Environment using the **IRES** Tool
