# Supplementary material for: Recognizing and Responding to Overt Racism Towards Medical Trainees: Using the IRES Tool and Scripted Language
Source: MedEdPORTAL. 2024 Oct 24;20:11453. doi: 10.15766/mep_2374-8265.11453 (PMC11500618; doi:10.15766/mep_2374-8265.11453)
Supplement: Supplementary file 1 — Facilitator Guide.docxSlide Deck.pptxPractice Cases.docxIRES Tool.docxScripted Language.docxPostworkshop Evaluation.docx [file mep_2374-8265.11453-s001.zip › E. Scripted Language.docx]

Appendix E. Scripted Language Handout

Use this handout during each practice case along with the IRES Tool (Appendix D). Read phrases out loud to your partner when you are ready to practice responding to the aggressor in each practice scenario. Try using different phrases and see if any of them feel more natural for you. Feel free to alter the wording and ask for feedback from your partner.

| Scripting to Respond to Racism |
| --- |
| We do not tolerate racist/bigoted/derogatory language here. It is harmful to our staff members and other patients.  I would like to provide care for you, but I found that comment very disrespectful to me and my team. We will need to step away for a moment.  We don’t tolerate the use of language like that in this hospital.  If you continue to use racist language, I will not be able to continue to provide care for you at this time. We will need to end this visit.  This is a member of our team, if you disrespect them, you disrespect our whole team.  We want to take care of you, but everyone on this team deserves respect and if you disrespect one of us, then you disrespect everyone. We can’t tolerate that.  I would like to provide you care, but I cannot tolerate your language. We are going to need to step away for a moment. |

| Debriefing the Learner |
| --- |
| The language that individual used was very offensive and inappropriate and we will not tolerate that type of behavior.  I am here to support you and hold people accountable for their comments or actions.  I don’t feel it is appropriate for you to be subjected to this kind of language and with your permission I would like to assign you to another patient.  I am not sure what you need right now, but I am here to support you.  That was difficult for me to hear that comment and I can’t imagine how you must have felt.  Let me know if you need some time to process what just happened. I am available to talk now or whenever you need support.  Experiences like this can be traumatic. I want to always remind you of the support systems we have here. |
