# Supplementary material for: Recognizing and Responding to Overt Racism Towards Medical Trainees: Using the IRES Tool and Scripted Language
Source: MedEdPORTAL. 2024 Oct 24;20:11453. doi: 10.15766/mep_2374-8265.11453 (PMC11500618; doi:10.15766/mep_2374-8265.11453)
Supplement: Supplementary file 1 — Facilitator Guide.docxSlide Deck.pptxPractice Cases.docxIRES Tool.docxScripted Language.docxPostworkshop Evaluation.docx [file mep_2374-8265.11453-s001.zip › A. Facilitator Guide.docx]

# *Recognizing and Responding to overt Racism Towards Medical Trainees: Using the ires tool and scripted LANGUAGE*

# Appendix a. Facilitator Guide

## Framing the Workshop

- This is an interactive faculty development workshop with the aim of training faculty on how to identify and respond to overt racism directed towards a trainee in the clinical environment.
- Medical trainees from diverse backgrounds face racism and discrimination during clinical rotations. The source may be patients, family, peers, attending physicians, or nursing staff.
- Exposure to chronic racism in the learning environment impacts significantly impacts wellbeing, leads to burn out and can impact performance.
- Microaggressions may be subtle or ambiguous and also need to be addressed, however this workshop specifically aims to equip faculty to react to egregious racism, such as the use of slurs or other violent language.
- The harm of a racist encounter directed towards a learner is amplified if there is faculty present who either do not respond, or do not respond appropriately.
- Appropriate responses to an incident of overt racism:
  - Do not minimize what has happened – deflection, humor, trying to align or negotiate may cause more harm.
  - Set firm boundaries and do not engage with the aggressor
  - Empower the learner, not the aggressor
  - Understand the importance of debriefing

Educational Objectives:

By the end of this workshop, you will be able to:

1. Differentiate between the concepts of bias, microaggressions and overt racism.
2. Contrast the response to microaggressions versus the response to overt racism.
3. Understand the role of academic faculty as an upstander and ally to trainees from diverse backgrounds.
4. Apply the Identify-Respond-End-Support (IRES) tool and scripted language to interrupt racism in a clinical encounter.

## What this Workshop Does *Not* Specifically Address or practice

- Responding to microaggressions or more subtle offenses. Some helpful resources for this topic are as follows:
  - **INTERRUPT**: DallaPiazza M, Padilla-Register M, Dwarakanath M, Obamedo E, Hill J, Soto-Greene ML. Exploring racism and health: an intensive interactive session for medical students. MedEdPORTAL. 2018; 14:10783. https://doi.org/10.15766/mep_2374-8265.10783
  - **OTWFTD:** Sotto-Santiago S, Mac J, Duncan F, Smith J. "I Didn't Know What to Say": Responding to Racism, Discrimination, and Microaggressions With the OWTFD Approach. MedEdPORTAL. Jul 31 2020;16:10971. doi:10.15766/mep_2374-8265.10971
  - **A.C.T.I.O.N:** Ganote C, Souza T, Cheung F. Microresistance and ally development: Powerful antidotes to microaggressions. Psychologist. 2007;62:271
- Responding when the attending physician is the target. We acknowledge that this is an unfortunately common occurrence that needs to be addressed, particularly on an institutional level. For this exercise, we will focus on instances where a learner is the target and the important role of faculty in creating safe learning environment.
- Clinical emergencies and situations where mental status of a patient is altered.

## Our Approach


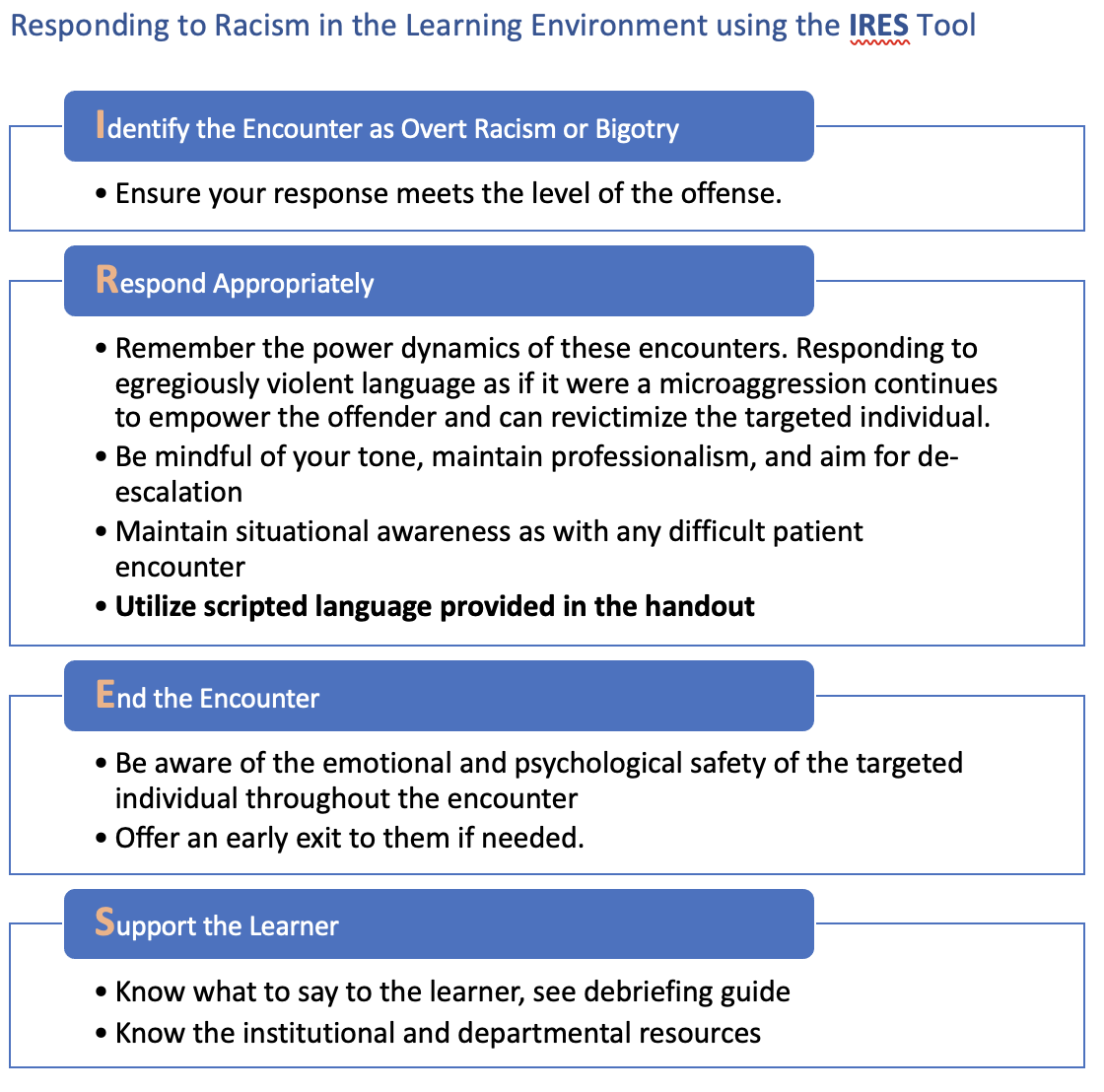


IRES Framework + Scripted Language: Both will be provided to participants. The goal is to emphasize identifying egregious racism and utilize scripted language to overcome the fight/flight/freeze response that can prevent an adequate and timely response. Practicing scripted language helps make this “muscle memory”.

Author Owned Image

## Recommended Structure

Facilitators: We recommend at least two facilitators.

Participants: Approximately 10-24 participants.

May be virtual or in-person

### Optional Review for Facilitators

- Jones CP. Levels of racism: a theoretic framework and a gardener's tale. Am J Public Health. Aug 2000;90(8):1212-5. doi:10.2105/ajph.90.8.121
- Lall MD, Bilimoria KY, Lu DW, Zhan T, Barton MA, Hu YY, Beeson MS, Adams JG, Nelson LS, Baren JM. Prevalence of discrimination, abuse, and harassment in emergency medicine residency training in the US. JAMA Netw Open. 2021;4(8):e2121706. doi:10.1001/jamanetworkopen.2021.21706
- McKillip KM, Moss RFF. Power, Silence, and Debriefing: Hidden Harms When Palliative Teams Encounter Racism. J Pain Symptom Manage. Jun 2021;61(6):1311-1315. doi:10.1016/j.jpainsymman.2021.01.128
- Osseo-Asare A, Balasuriya L, Huot SJ, et al. Minority Resident Physicians' Views on the Role of Race/Ethnicity in Their Training Experiences in the Workplace. JAMA Netw Open. Sep 7 2018;1(5):e182723. doi:10.1001/jamanetworkopen.2018.2723
- Racial Equity Tools Glossary. [Internet]. Available from: https://www.racialequitytools.org/glossary
- Souza TJ. Responding to Microaggressions in the Classroom: Taking A.C.T.I.O.N. Faculty Focus Premium [Internet]. 2018 [cited 2024 May 4]. Available from: Madison, WI: Magna Publications.
- Yuce TK, Turner PL, Glass C, Hoyt DB, Nasca T, Bilimoria KY, Hu YY. National evaluation of racial/ethnic discrimination in US surgical residency programs. JAMA Surg. 2020;155(6):526. doi:10.1001/jamasurg.2020.0260

### Suggested 1.5 Hour Session Plan OVerVIew

Didactic Portion

- 30 minutes. Presentation by the facilitators using slides 1-44.

Case Based Practice

- Case 1 Practice: 10 minutes. Slide 34. Read the case as a group, then participants separate into breakout groups of 2-3. During the break out group, participants practice saying scripted language to respond to the case.

Case 1 Large Group Debrief: 10 minutes. Slides 35-38. All participants exit their break out rooms, facilitators debrief the case using the questions in the slide deck.

- Case 2 Practice: 10 minutes. Slide 39. Read the case as a group, then participants separate into breakout groups of 2-3. During the break out group, participants practice saying scripted language to respond to the case.
- Case 2 Large Group Debrief: 10 minutes. Slide 40. All participants exit their break out rooms, facilitators debrief the case using the questions in the slide deck.

Wrap-Up

- 15 minutes. Slides 41-44. Wrap up slides, take aways and questions.

Post-Session Survey

- 5 minutes. Slide 45.

### Detailed Workshop Guide

Prior to the workshop, we suggest either the day before or day of, email the IRES tool handout (Appendix D) and the scripted language handout (Appendix E) to participants.

Slides 1-11: Introduction and Establishing a Safe Environment

- Have the Slide Deck (Appendix B) pulled up to present. Introduce the topic of this workshop. This is a faculty development workshop aimed at giving faculty the tools and skills to respond to incidents of overt racism directed towards trainees in clinical encounters. Facilitators should introduce themselves and why this topic is meaningful to them. Tell participants about the structure of the work shop.
- Slide 2: Facilitators may conduct a brief icebreaker. This is especially important if participants do not know each other. We have found that this is an important step to encourage participation and a psychologically safe space given the difficult nature of this topic.
- Slides 3-4: Discuss the why this topic is important at your institution or department. Also highlight that many medical organizations have clear statements on the importance of addressing racism in medical education.
- Slides 5-7: Here we acknowledge that we are all learning, and set ground rules to maintain a safe space. These rules are written on the slides, feel free to add others if needed.
- Slides 8-11: Share the learning objectives. Let participants know that the IRES tool and scripted language will be reviewed in more detail towards the end of the didactic session. They will get an opportunity to practice using them during the case scenarios. Review the vocabulary in the slides. Bias and microaggressions will be explored in more detail later in the didactic session.

SLIDES 12-17: Historical and Structural Racism in Medical Education

- Introduce this section as a brief overview of the ways in which historical and structural racism influences the environment in which medical trainees from diverse backgrounds come to learn. As we will talk more about, it is important to understand that medical education itself has been used to uphold racist structures, that remnants of this continue to the present day, and that this environment impacts how trainees experience episodes of interpersonal racism.
- Slide 13: We are focusing on race but this can apply to any marginalized characteristic. This model, developed by Dr. Camara Jones, helps to visualize how an individual may experience racism from multiple levels, and the confluence will shape their how they experience an environment. ​This workshop focuses on the interpersonal level, but as we can see here, it is important to briefly touch on how learners experience systemic racism specifically in academic medicine
- Slide 14: We will briefly illustrate three commonly cited areas in which trainees may experience the confluence of the medical field and structural racism, though it is by no means an exhaustive list. ​We’ll touch on curricular racism, which is when educational materials and concepts usually unknowingly perpetuate racism, Inequitable practices and outcomes for marginalized patients whom they may identify with, and then a lack of representation and community within the medical field. ​
- Slide 15: Curricular racism is presence of racism and misuse of the concept of race in educational materials and methods. It promotes, at minimum, propagation of implicit bias against diverse individuals who are often pathologized, portrayed as less than or are absent in these materials. ​For example, emphasizing the presence of a disease process within a certain group without addressing structural causes of racial health disparities, instead implying race as a genetic risk factor.​ Read the rest of the examples.
- Slide 16: Racism in medical education leads to bias against marginalized patient groups. For trainees belonging to these groups who witness and learn inequitable treatment based on race, there can be a tension resulting in lack of belonging and internalized racism. These are just some of the studies that have shown when corrected for other factors such as socioeconomic status and access to medical care, that racial or gender bias has concrete effects on patient care and outcomes. ​Read examples on slide.
- Slide 17: Lastly, we will touch on underrepresentation in medicine as another factor contributing to the experience of systemic racism for trainees from diverse backgrounds. ​The US has a long history of policies and practices that result in the exclusion of certain groups of people from positions of prestige or power, including the medical field. ​Since like many institutions, medical education was segregated in the late 19th and early 20th century, black physicians formed their own medical schools to meet the need of training future black doctors. In this time period, there was also a proliferation of commercial medical schools with wide variations in quality.​At the turn of the century, to modernize and standardize medical education, the AMA commissioned the Flexner Report, published in 1910 after it’s author had inspected all 155 medical schools in North America. The report resulted in the closure of 50% of medical schools. At the time there were only 9 black medical schools to educate all of the united states black physicians. After the report 6 were closed. Howard, Meharry and Morehouse survived, which greatly limited the capacity to train black physicians for years to come until the desegregation of medical schools. ​

The lack of access to a medical education was compounded through the years by ongoing discriminatory practices and socioeconomic factors that lead to an environment where becoming a physician as person of color required overcoming a multitude of hurdles.​

- Slide 18: This is a visualization of how all of these systemic factors influence the learning environment in medical training and negatively impact the sense of belonging for trainees from diverse backgrounds.
- Slides 19-21: Review the content of these slides that focus on the data around trainee experience of racism on a structural and interpersonal level.

Slides 22-26: Interpersonal Racism and Medical Training

- Where we just reviewed structural racism, the next few slides will go over the types of interpersonal racism including implicit bias, microaggressions and overt racism. Emphasize that all of these are harmful and are vital to address, but for the purpose of this workshop, the focus is on learning how to respond in the moment to acts of overt racism. Also reiterate that while this structure and our workshop has focused on race, interpersonal discrimination applies to any other marginalized identity.
- Slide 23: Implicit Bias, previously defined, has several different types
  - In group bias- preferring or favoring those who are similar to ourselves.
  - ​Illusory bias- false correlation, seeing a relationship between things that does not exist
  - Confirmation bias Giving more weight to evidence that supports your already held, preferred belief​
- Slides 24-25: Microaggressions. Review definition on slide. Even if they occur unconsciously micro aggressions have been shown to be harmful to the physical and emotional well being of the individual, and to have harmful, downstream systemic effects.​ There has been more recent debate about terminology, however the most important thing here is to notices that someone who doesn’t experience microaggressions frequently, these things can look innocuous while being very harmful to the targeted individual.​ Some proposed types of microaggressions are as follows:
  - Micro invalidation- often unconscious  verbal comments and behaviors that invalidate, exclude, negate or nullify the psychological thoughts, feelings or epiretinal realty of the individual​.Example: Color blindness, alien in own land​
  - Micro insult- often unconscious behavioral or verbal remarks that convey insensitivity or rudeness and demeans a person’s identity.​ Example: Ascription of intelligence or criminal status, idea that values and communication styles of these individuals are abnormal​
  - Microassult- often conscious explicit racial derogations by violent verbal or non verbal attack meant to hurt to the victim through name calling, avoidant behavior or purposeful discriminatory actions. ​*Note that it could be argued that this is not distinguishable from overt racism. As participants will see in Practice Case 2 (Appendix C), when the intent to harm is obvious it doesn’t matter what you call it, the important thing is to be able to respond.
- Slide 26: The focus of this faculty development is to provide language for instances of overt racism since intensity of these situations can catch us off guard- resulting freezing, fight or flight moments in which we may not have the ability to respond appropriately without scripted and practiced language. ​

SLIDES 27-31: Tools for Taking Action

- In preparation for our role play practice cases, we want to first look at one of the published tools available to help respond to microaggressions and contrast it with our proposed tool to respond to episodes of overt racism.
- Slide 28: The published tools for responding to microaggressions form the basis of a response around asking clarifying questions or inquiring about the meaning of a particular statement
- Slide 29-30: Read information on the slides. Here, we emphasize that for an incident of overt racism such as a slur, these tools are no longer useful. It would not be appropriate to address this with a clarifying question or to investigate intent With overt racism, intent is known and is purposefully harmful so clarifying questions are not appropriate Furthermore, without a practiced response, a person may fall back on a flight-fight-freeze-fawn response which can also cause harm to the target by escalating, minimizing, or brushing off.
- Slides 31-32: Introduce the IRES Tool as detailed in the slide and reinforce the importance of responding in these situations .

SLIDES 33-40: Case based practice And debriefings

- There are two two practice cases in this section. The cases are written both in the slides (Slides 34 & 39) and available as a handout (Appendix C). Before showing the first case, tell participants that they’ll be paired off to practice saying responding to two scenarios in which a learner experiences racism in a clinical setting. Instruct participants as follows:
- Facilitators demonstrate the case based role play as follows:
  - Ask participants to have their handouts for the IRES tool (Appendix D) and Scripted language (Appendix E) ready.
  - Go through the IRES tool as a large group
    - I- Identify. Ask participants to exam whether the incident feels to be an overt incident of racism
    - R-Respond. Ask participants to respond out loud to this simulated case using the scripted language. Facilitators can demonstrate by simply reading a phrase of their choosing from the scripted language hand off. Emphasize that the goal is to try out different phrases, explore how it feels to respond, and choose a phrase you are comfortable with to “keep in your back pocket” in case you are met with an incident.
    - E- End the encounter. Ask participants to practice language for ending the encounter and exiting with the learners.
    - S-Support the learner. Ask participants to practice supporting the learner using scripted language provided. Facilitators can demonstrate by reading from the handout.
- Note that it is OK to try different phrases or change them in a way that feels easy to use for the individual. The most important thing is to have language that is simple and easily available to the participant in a stressful encounter. Reiterate that we do not want anyone to act out the role of the aggressor or to use offensive language. Encourage pairs to give each other feedback and share any experiences they have had.
- It is important that facilitators be familiar with their institution’s own policies regarding patient behavior.
- Assign participants randomly to breakout rooms if virtual or pairs if in person. If there are odd numbers, facilitators may pair with a participant.
- Allot 10 minutes for each breakout session. After each case, go through the debriefing questions detailed in the slide deck.
  - Slide 35 Debrief #1
    - What was challenging about that case?
      - Some participants may express doubt that something this egregious actually occurs. If this comes up, see studies by Yuce, 2020 and Lall, 2021 in references.
    - Did it feel hard to respond? Would it be harder without having a scripted phrase on hand?
    - What could you use more help with?
  - Slide 36-37 Debrief #1
    - What emotions do you feel as the bystander? or as the target?
    - What is your gut reaction response?
      - These slides aim to emphasize that overt racism in the moment is a very emotionally charged, violent interaction that naturally ignites a stress response- fight, flight, freeze, fawn. It is a normal autonomic response that we have, but in order to adequately support the targeted trainee, we want to over ride this response by having prepared and direct language.
      - An article that does a good job explaining why a bystander response of asking clarifying questions, brushing off or freezing can be harmful to the target is Mckillip, 2021.
  - Slide 40 Debrief #2
    - What was challenging about this case?
    - Is it more difficult to respond to racism that is overt but perhaps less violent than say a slur? If so, why do you think that is?
    - If a patient was being physically threatening, would you feel more able or less able to respond?

Slides 41-46: Wrap up

- Review wrap up slides, emphasizing the importance of responding appropriately as a vital part of maintaining a psychologically safe learning environment for trainees from diverse backgrounds, as well as the role of faculty as allies and upstanders.
- Have a list of resources at your institution that faculty can provide to trainees who have experienced an incident of overt racism. This may be counseling services, your ACGME ombudsman, contacts or links to report incidents etc. We also recommend offering resources to faculty who have been effected by racism as this workshop could potentially be retraumatizing. Share the post session evaluation (Appendix F) with participants.

​
