# Supplementary material for: Recognizing and Responding to Overt Racism Towards Medical Trainees: Using the IRES Tool and Scripted Language
Source: MedEdPORTAL. 2024 Oct 24;20:11453. doi: 10.15766/mep_2374-8265.11453 (PMC11500618; doi:10.15766/mep_2374-8265.11453)
Supplement: Supplementary file 1 — Facilitator Guide.docxSlide Deck.pptxPractice Cases.docxIRES Tool.docxScripted Language.docxPostworkshop Evaluation.docx [file mep_2374-8265.11453-s001.zip › B. Slide Deck.pptx]

## Slide 1
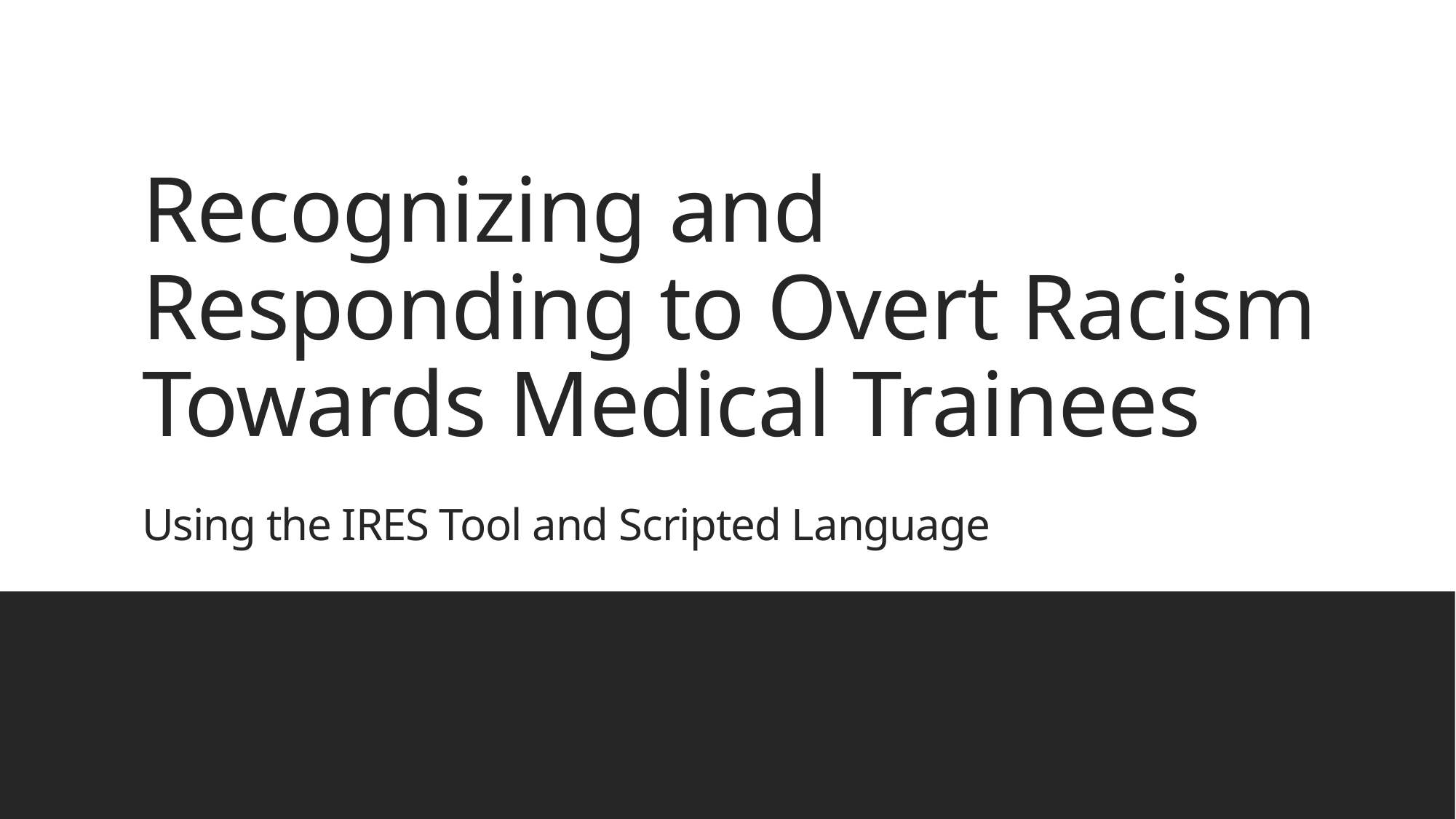

# Recognizing and Responding to Overt Racism Towards Medical Trainees Using the IRES Tool and Scripted Language

## Slide 2
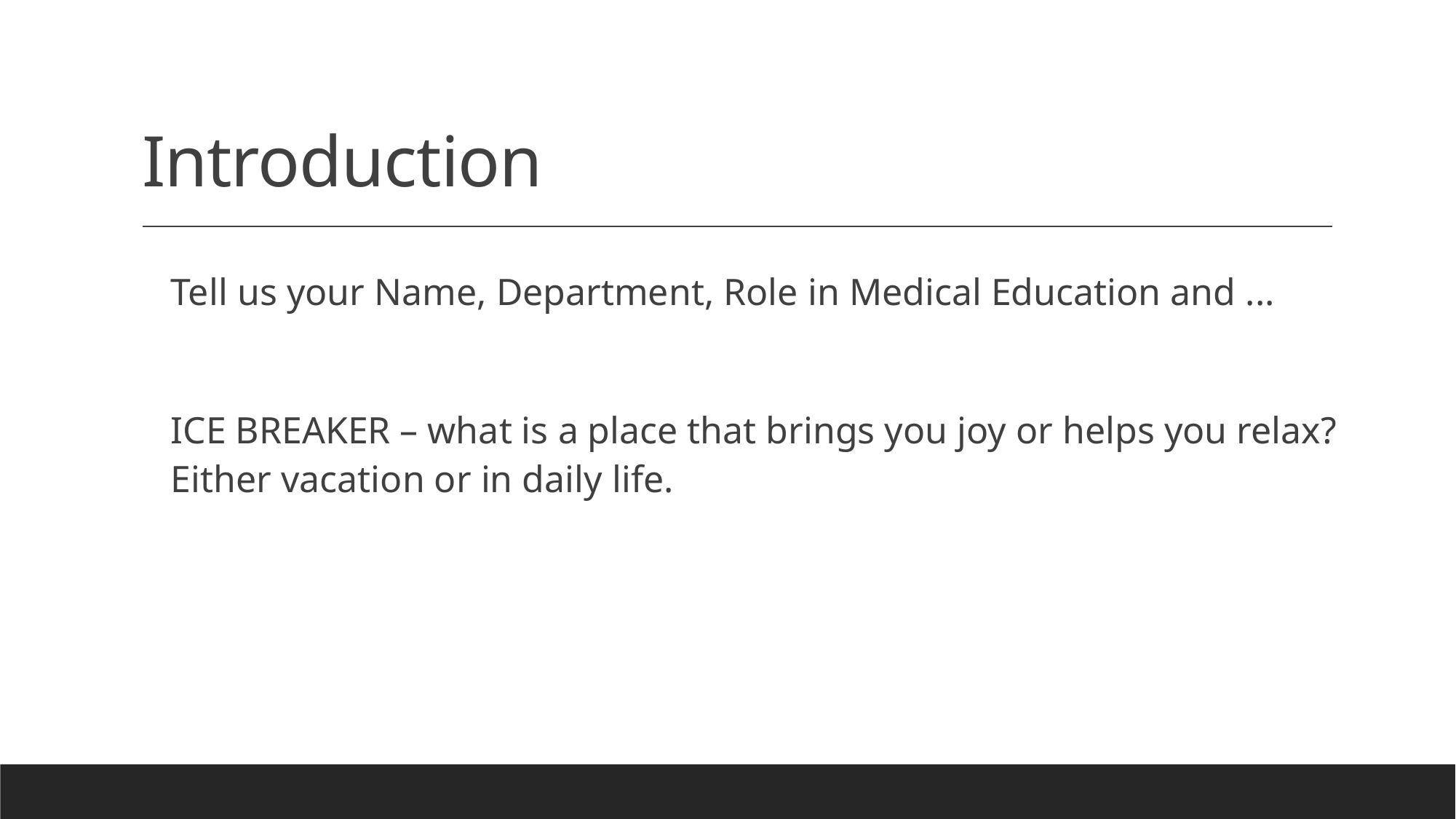

# Introduction
Tell us your Name, Department, Role in Medical Education and ...
ICE BREAKER – what is a place that brings you joy or helps you relax? Either vacation or in daily life.

## Slide 3
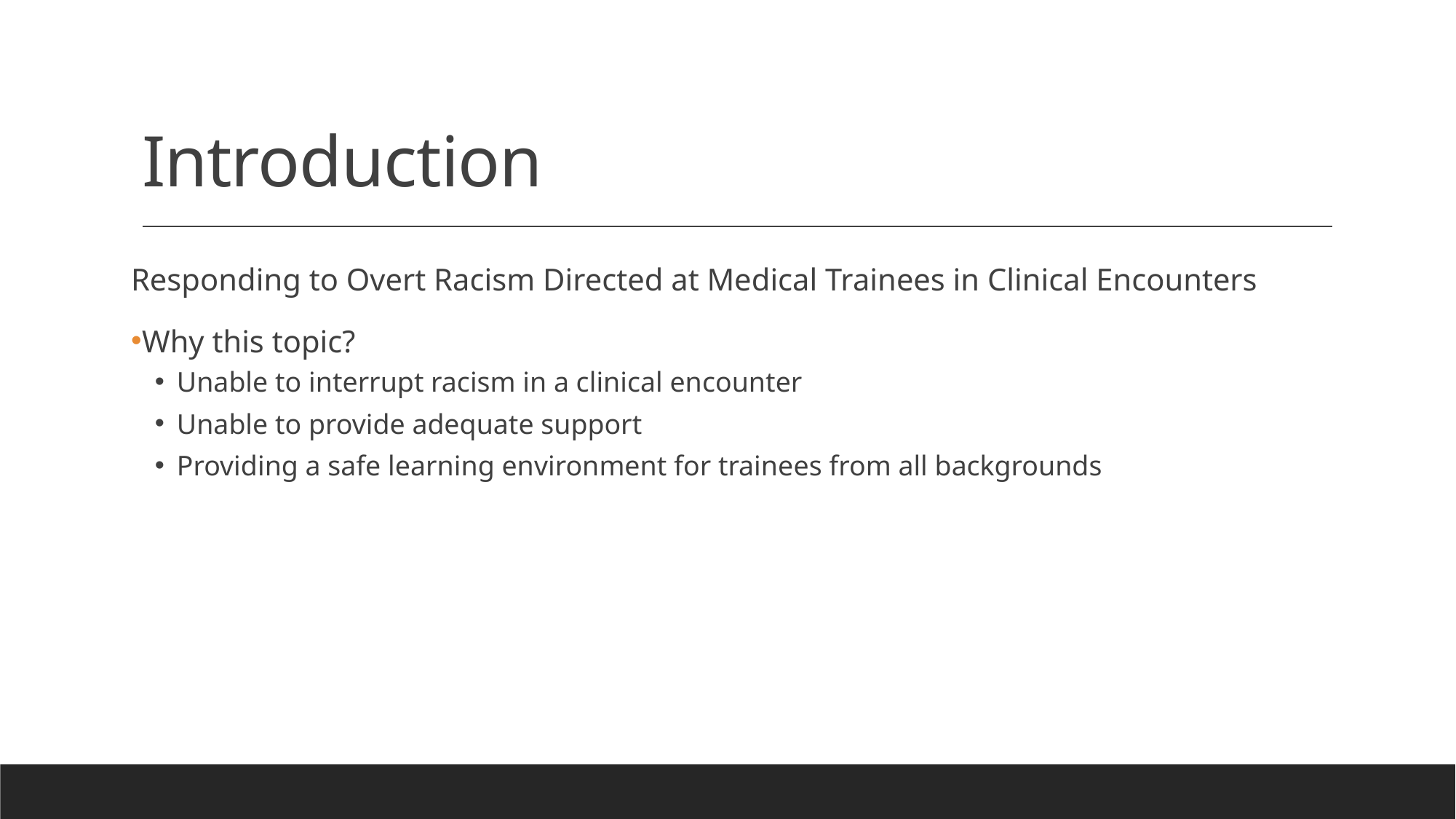

# Introduction
Responding to Overt Racism Directed at Medical Trainees in Clinical Encounters
Why this topic?
Unable to interrupt racism in a clinical encounter
Unable to provide adequate support
Providing a safe learning environment for trainees from all backgrounds

## Slide 4
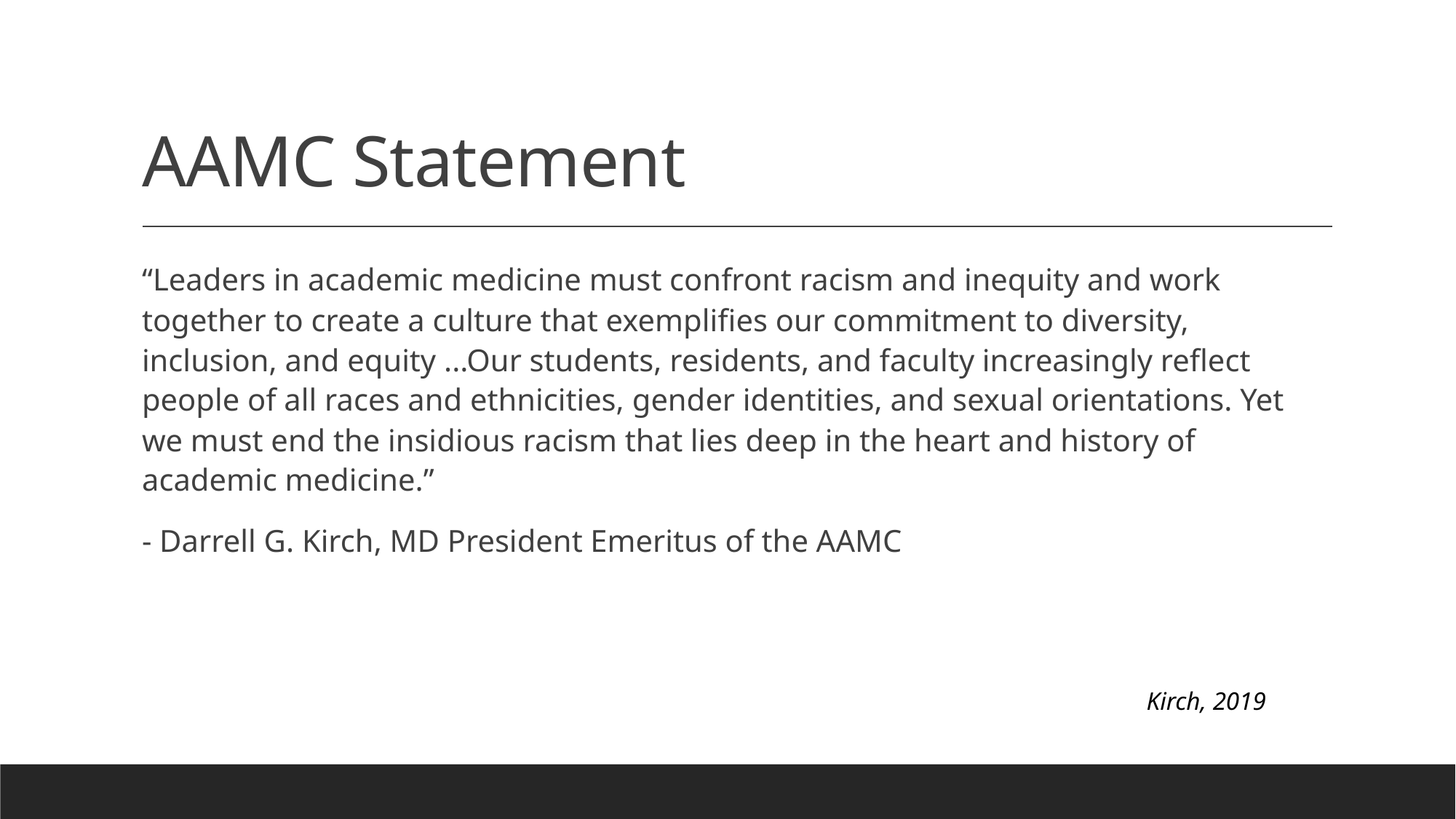

# AAMC Statement
“Leaders in academic medicine must confront racism and inequity and work together to create a culture that exemplifies our commitment to diversity, inclusion, and equity ...Our students, residents, and faculty increasingly reflect people of all races and ethnicities, gender identities, and sexual orientations. Yet we must end the insidious racism that lies deep in the heart and history of academic medicine.”
- Darrell G. Kirch, MD President Emeritus of the AAMC
Kirch, 2019

## Slide 5
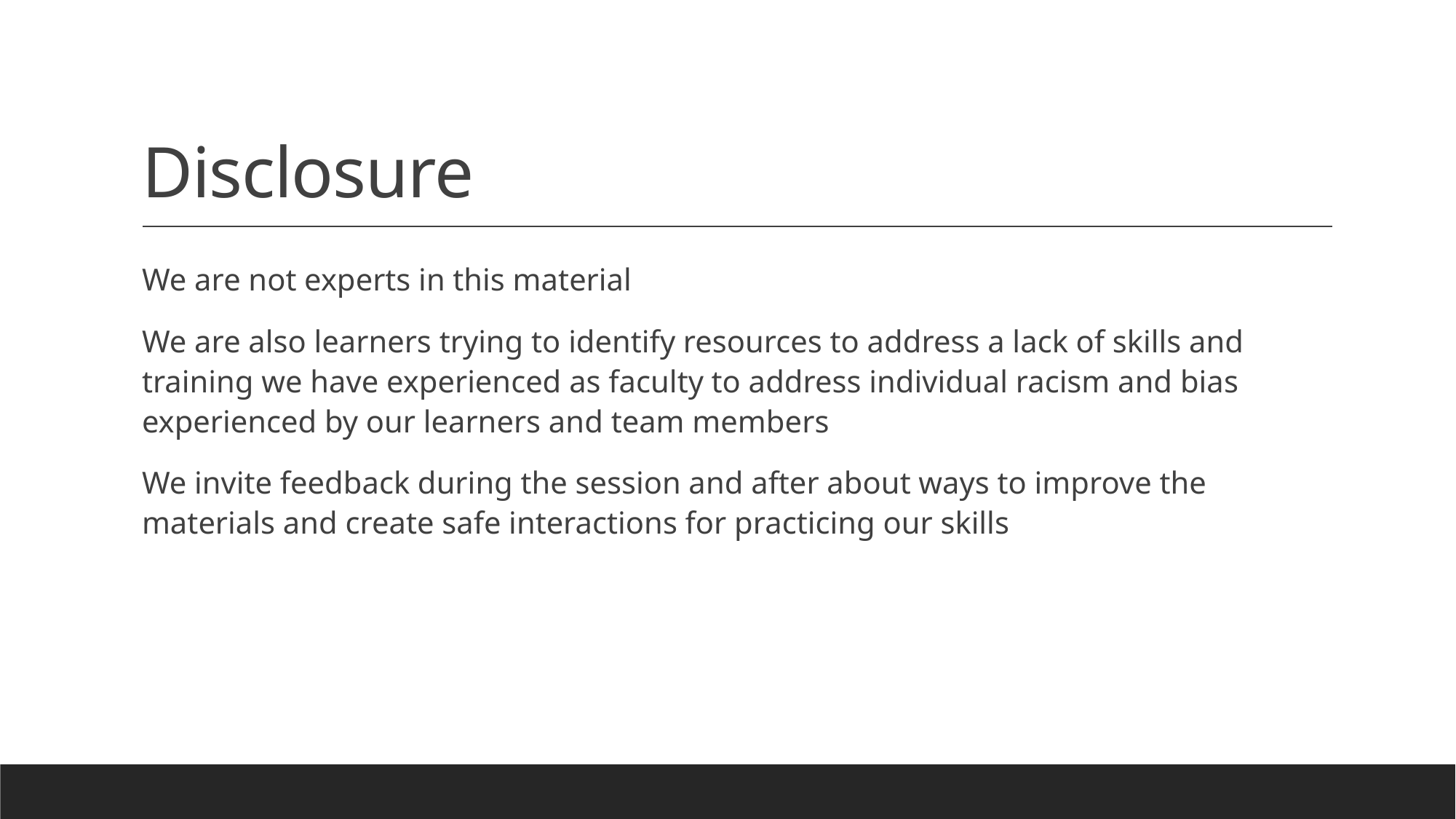

# Disclosure
We are not experts in this material
We are also learners trying to identify resources to address a lack of skills and training we have experienced as faculty to address individual racism and bias experienced by our learners and team members
We invite feedback during the session and after about ways to improve the materials and create safe interactions for practicing our skills

## Slide 6
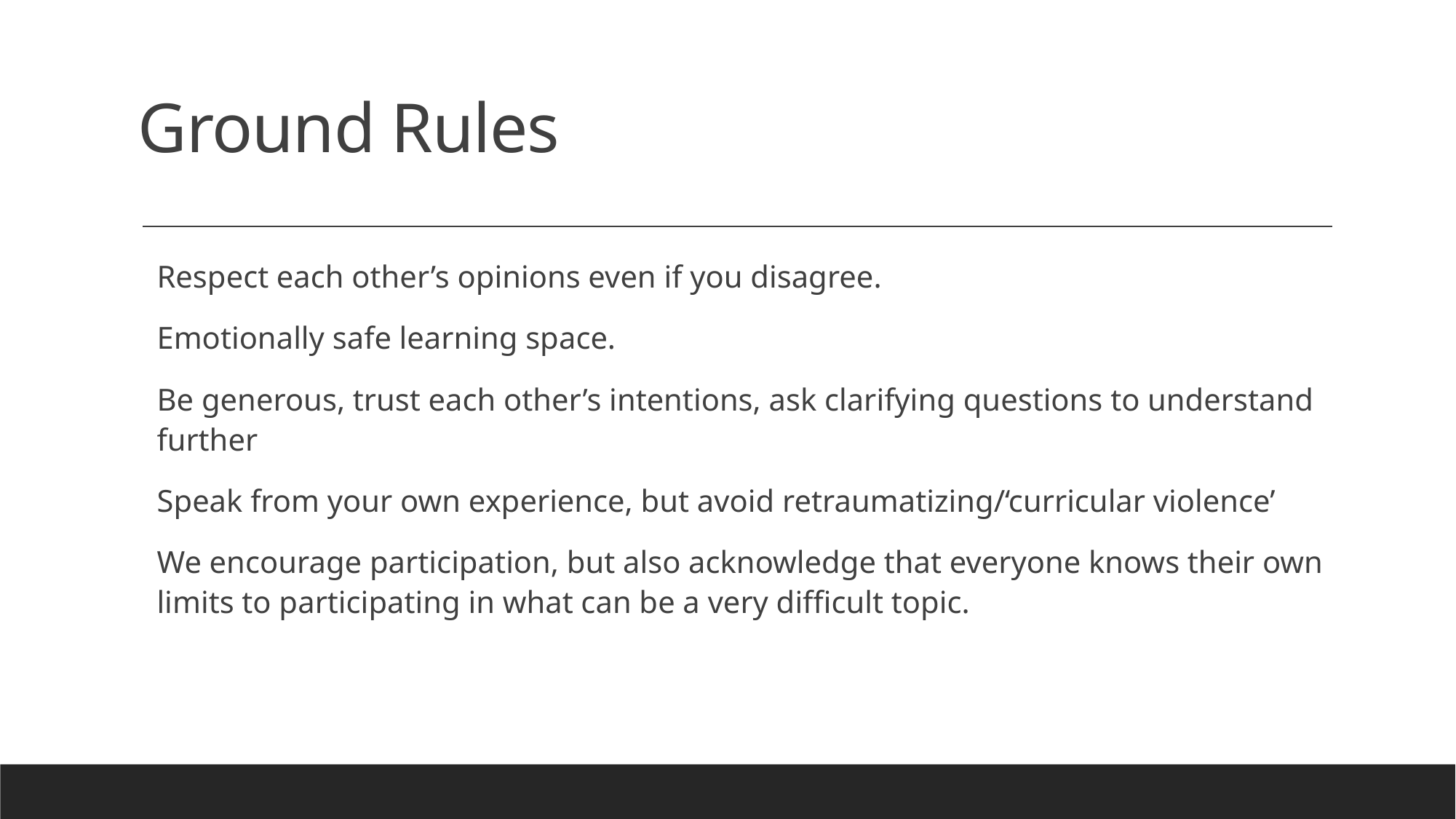

# Ground Rules
Respect each other’s opinions even if you disagree.
Emotionally safe learning space.
Be generous, trust each other’s intentions, ask clarifying questions to understand further
Speak from your own experience, but avoid retraumatizing/‘curricular violence’
We encourage participation, but also acknowledge that everyone knows their own limits to participating in what can be a very difficult topic.

## Slide 7
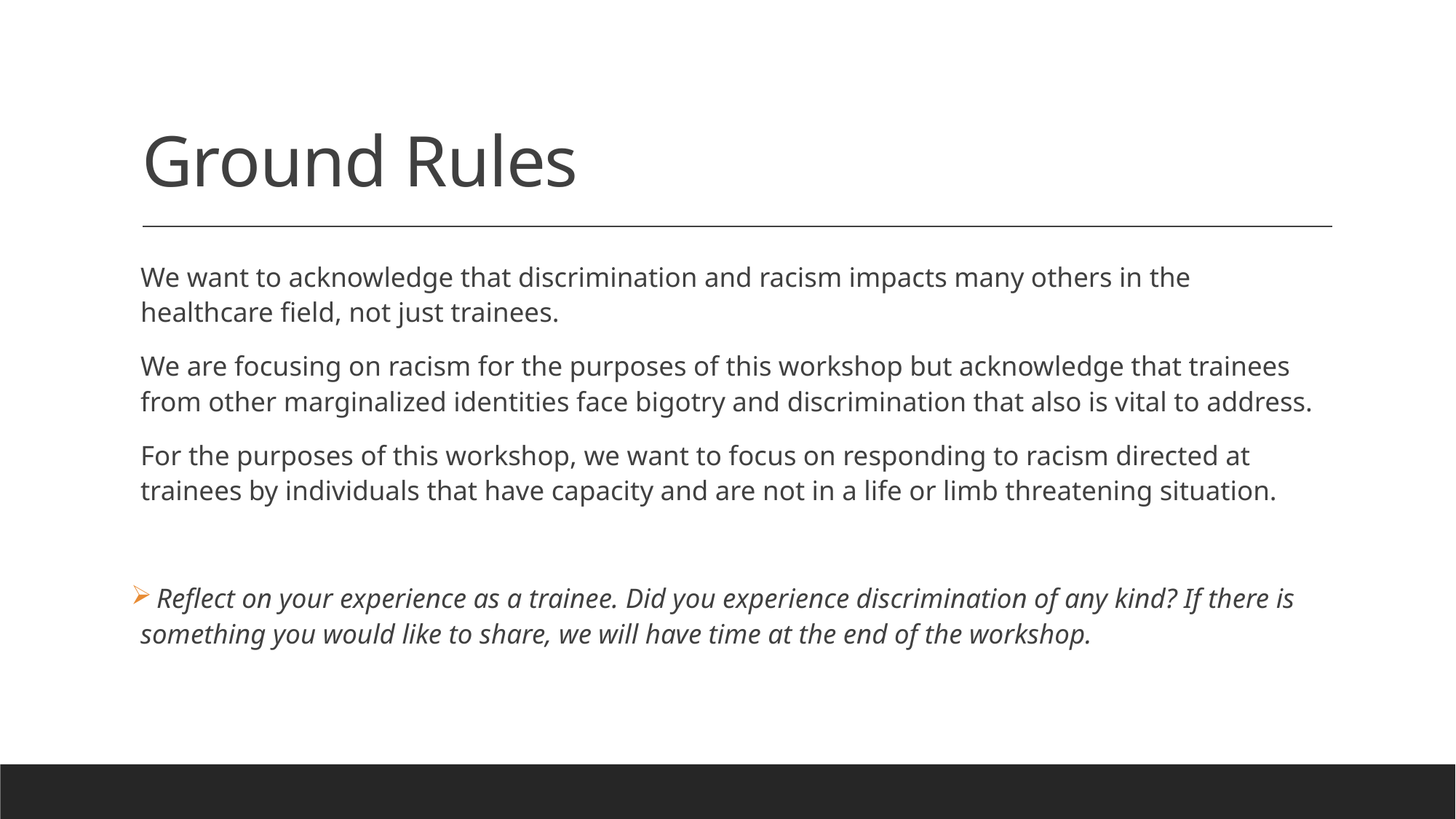

# Ground Rules
We want to acknowledge that discrimination and racism impacts many others in the healthcare field, not just trainees.
We are focusing on racism for the purposes of this workshop but acknowledge that trainees from other marginalized identities face bigotry and discrimination that also is vital to address.
For the purposes of this workshop, we want to focus on responding to racism directed at trainees by individuals that have capacity and are not in a life or limb threatening situation.
 Reflect on your experience as a trainee. Did you experience discrimination of any kind? If there is something you would like to share, we will have time at the end of the workshop.

## Slide 8
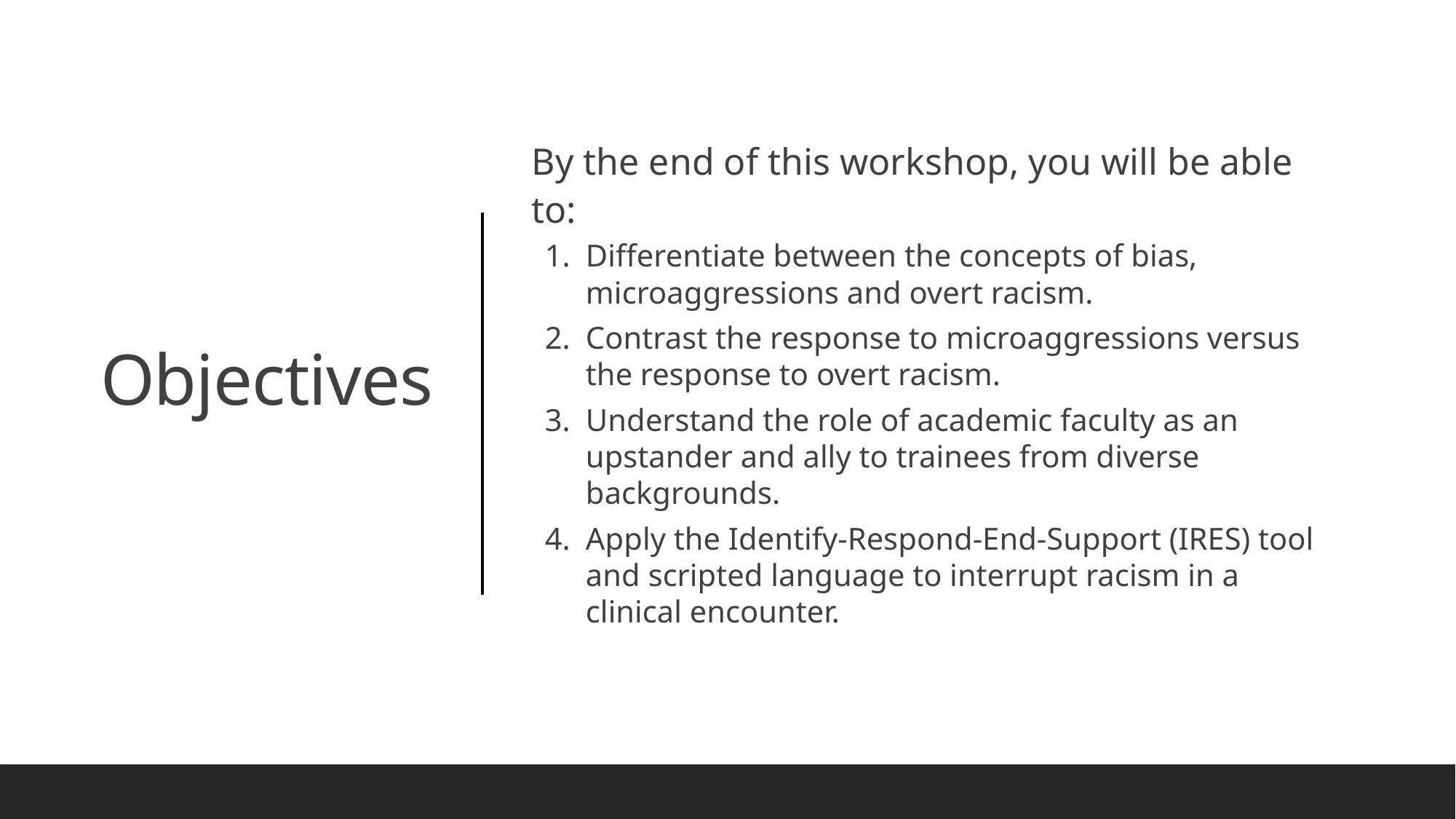

By the end of this workshop, you will be able to:
Differentiate between the concepts of bias, microaggressions and overt racism.
Contrast the response to microaggressions versus the response to overt racism.
Understand the role of academic faculty as an upstander and ally to trainees from diverse backgrounds.
Apply the Identify-Respond-End-Support (IRES) tool and scripted language to interrupt racism in a clinical encounter.
# Objectives

## Slide 9
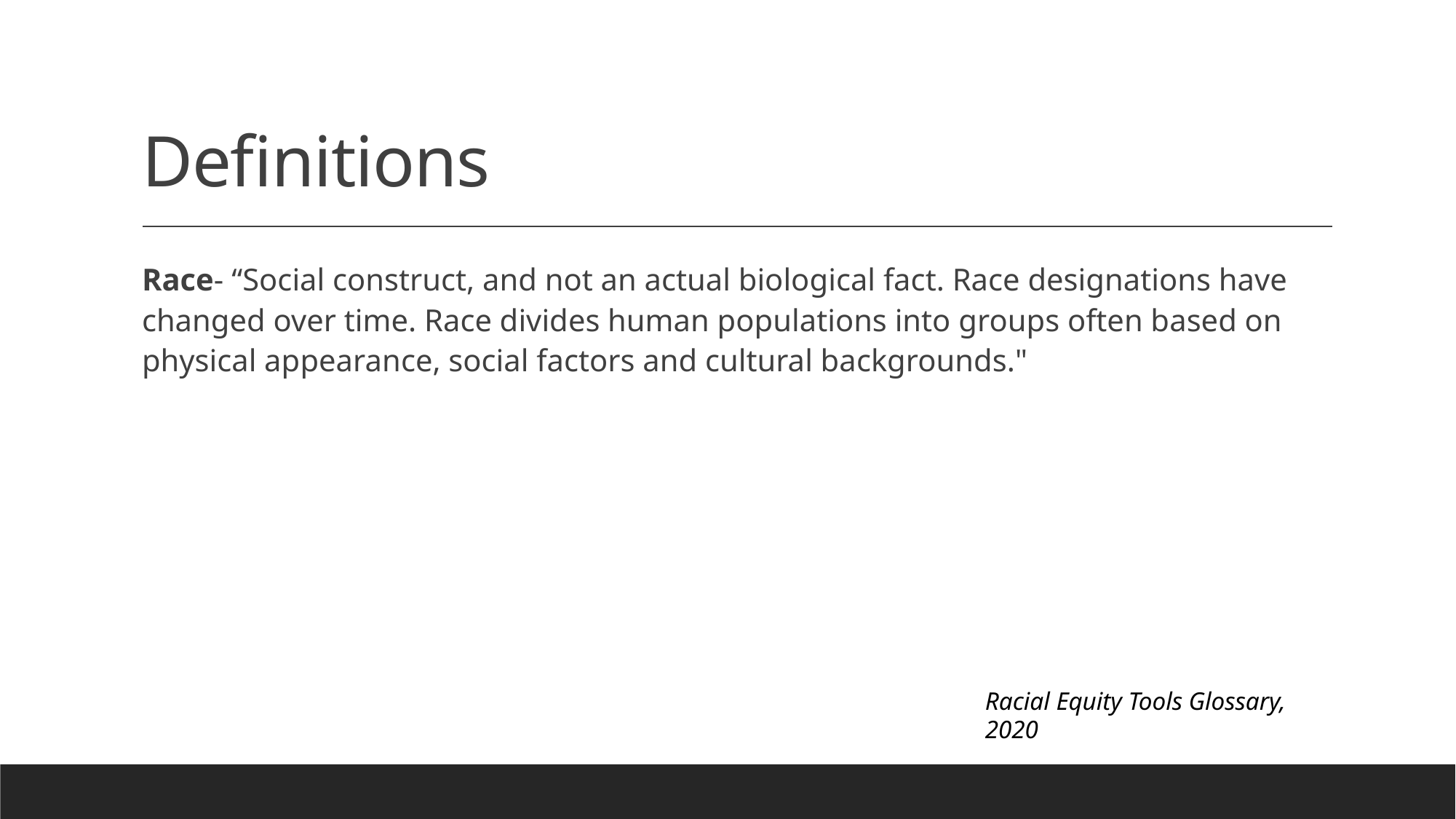

# Definitions
Race- “Social construct, and not an actual biological fact. Race designations have changed over time. Race divides human populations into groups often based on physical appearance, social factors and cultural backgrounds."
Racial Equity Tools Glossary, 2020

## Slide 10
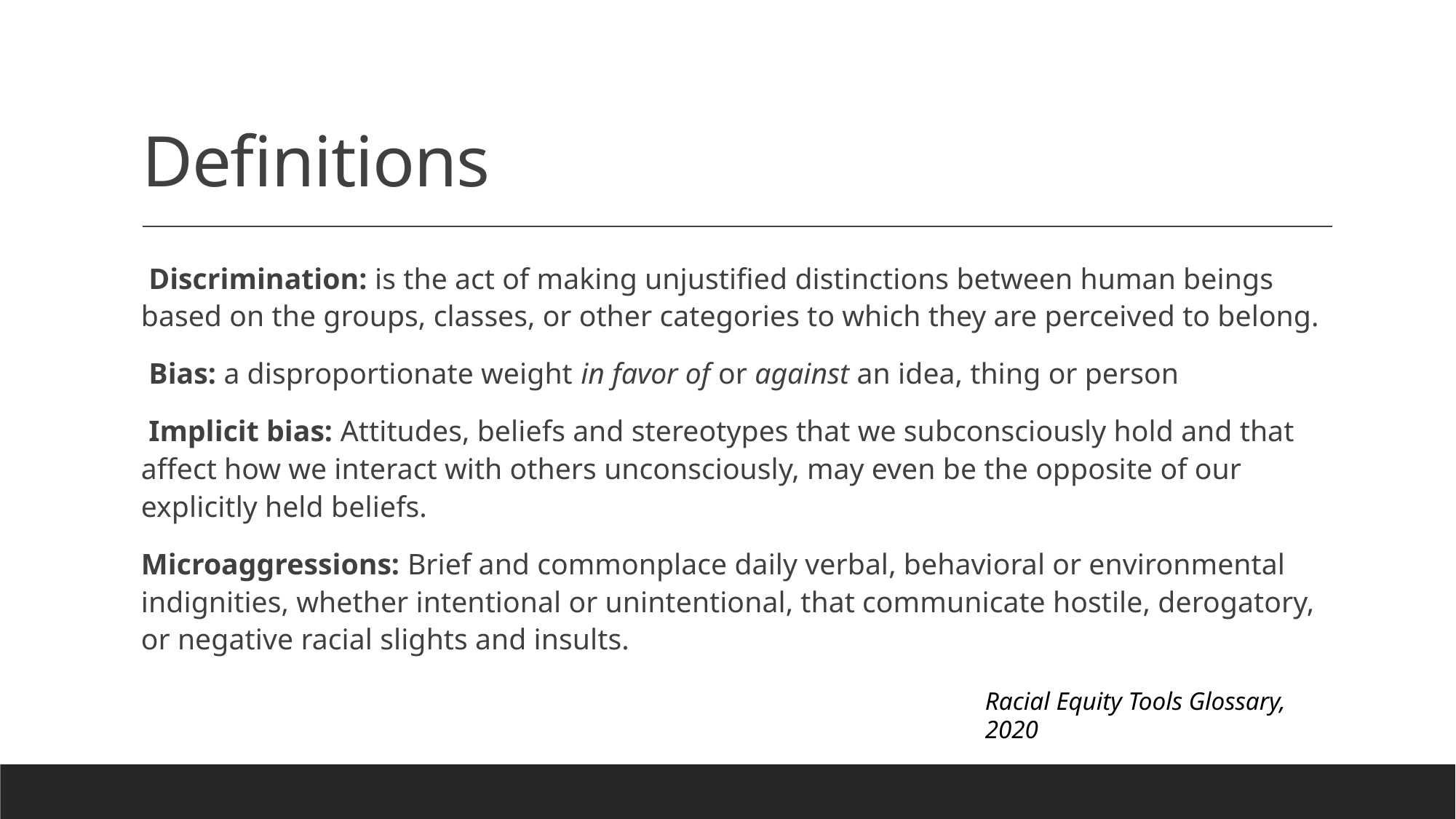

# Definitions
 Discrimination: is the act of making unjustified distinctions between human beings based on the groups, classes, or other categories to which they are perceived to belong.
 Bias: a disproportionate weight in favor of or against an idea, thing or person
 Implicit bias: Attitudes, beliefs and stereotypes that we subconsciously hold and that affect how we interact with others unconsciously, may even be the opposite of our explicitly held beliefs.
Microaggressions: Brief and commonplace daily verbal, behavioral or environmental indignities, whether intentional or unintentional, that communicate hostile, derogatory, or negative racial slights and insults.
Racial Equity Tools Glossary, 2020

## Slide 11
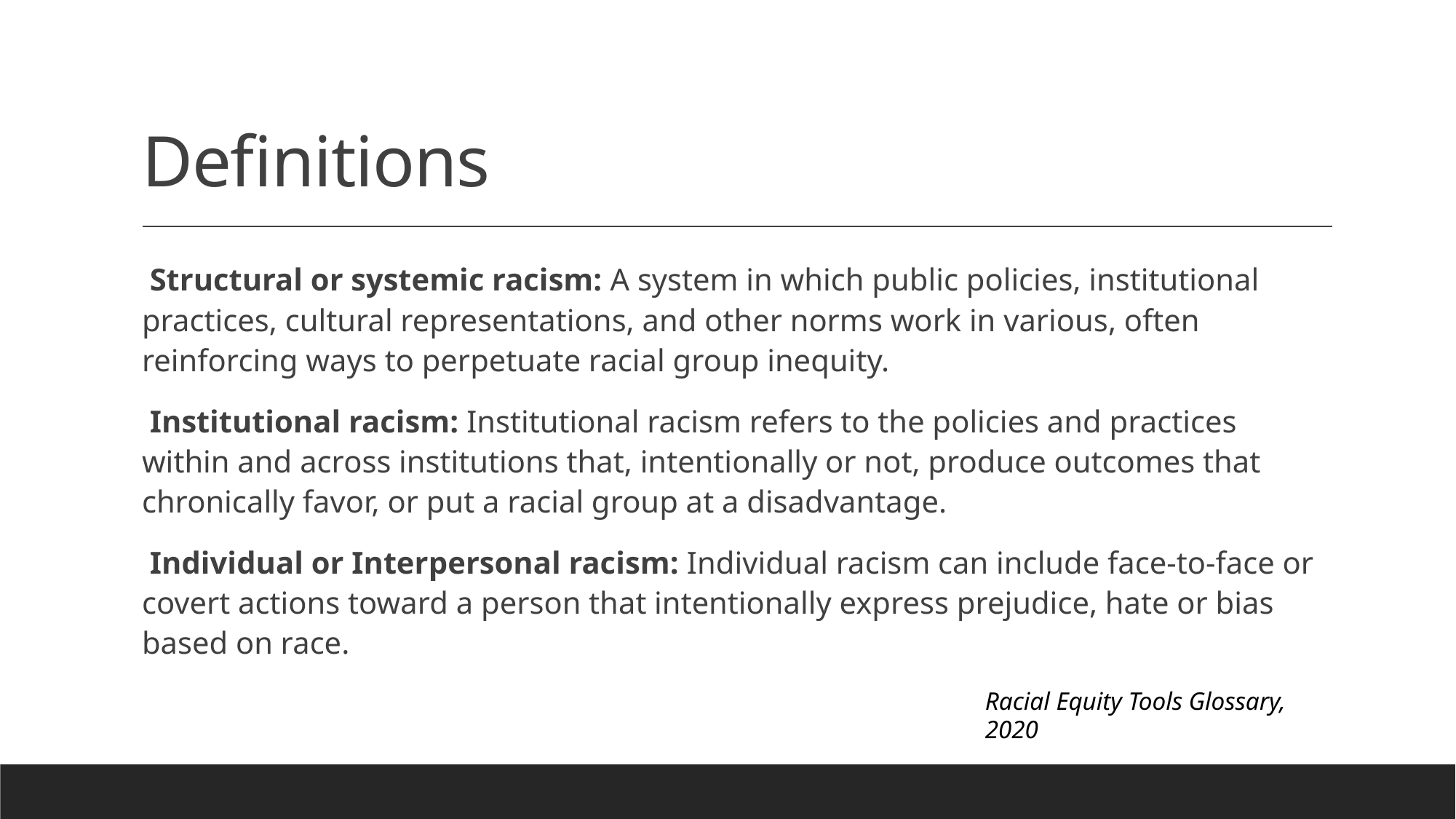

# Definitions
 Structural or systemic racism: A system in which public policies, institutional practices, cultural representations, and other norms work in various, often reinforcing ways to perpetuate racial group inequity.
 Institutional racism: Institutional racism refers to the policies and practices within and across institutions that, intentionally or not, produce outcomes that chronically favor, or put a racial group at a disadvantage.
 Individual or Interpersonal racism: Individual racism can include face-to-face or covert actions toward a person that intentionally express prejudice, hate or bias based on race.
Racial Equity Tools Glossary, 2020

## Slide 12
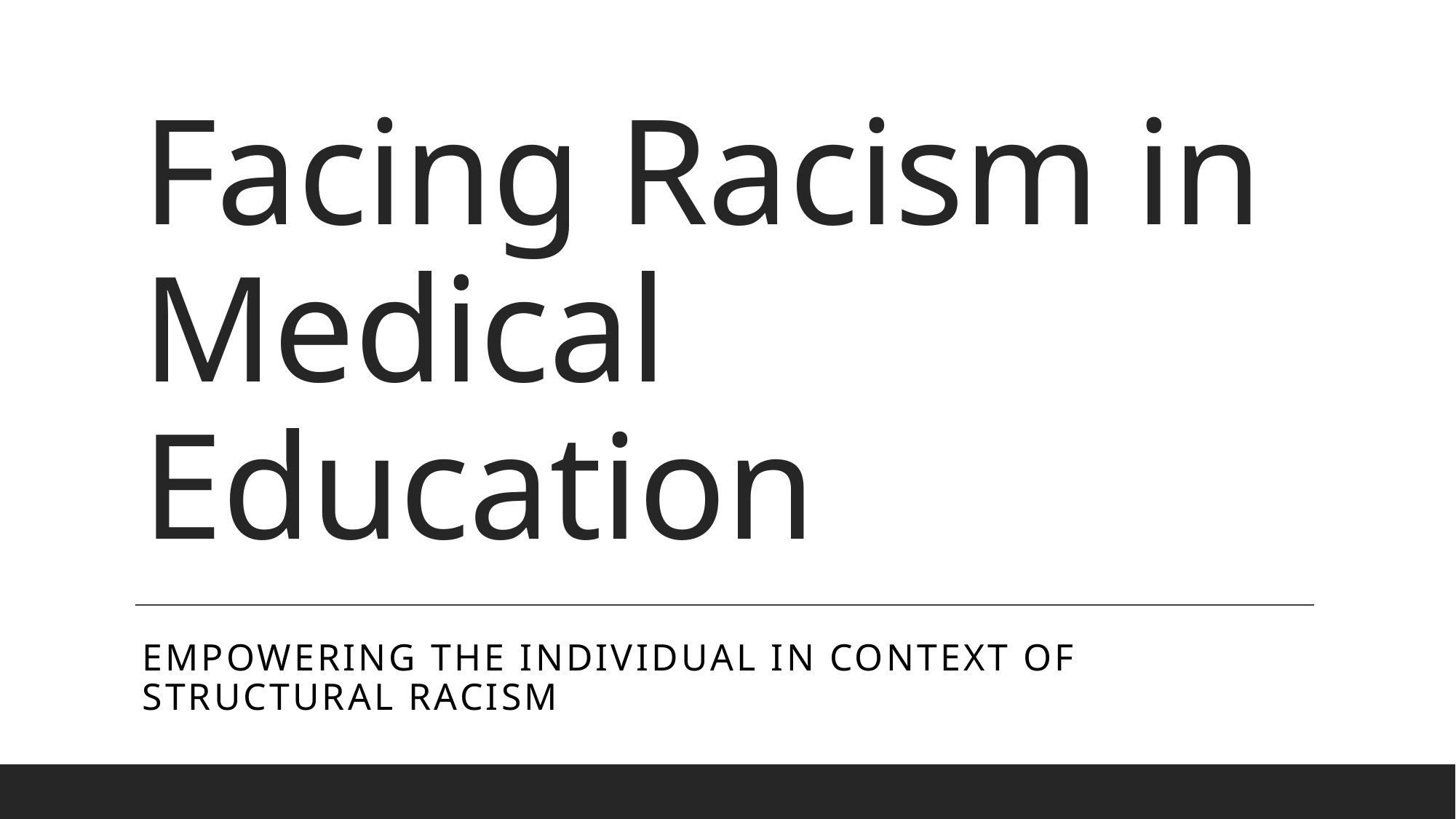

# Facing Racism in Medical Education
Empowering the Individual in Context of structural racism

## Slide 13
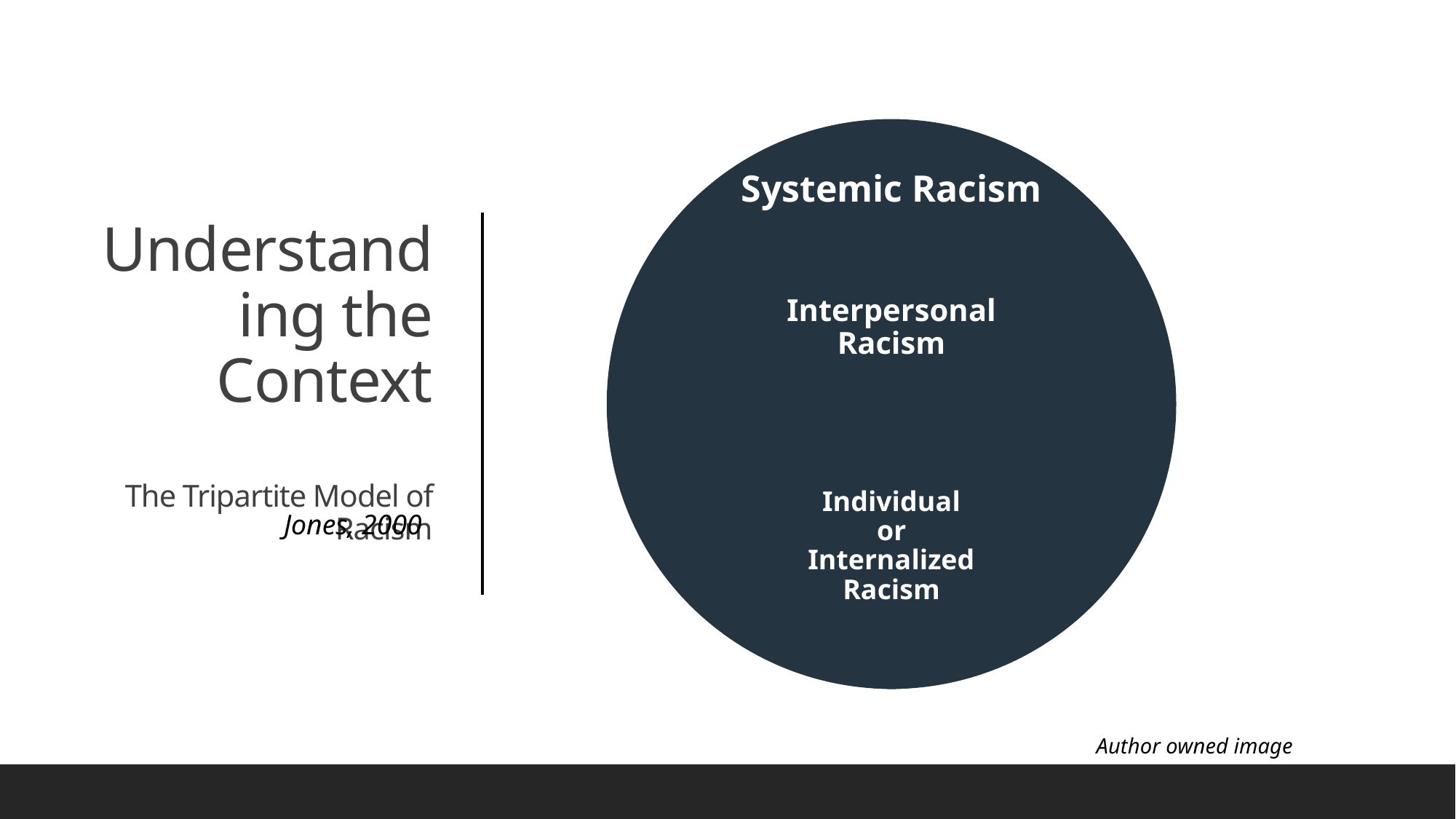

# Understanding the Context The Tripartite Model of Racism
Jones, 2000
Author owned image

## Slide 14
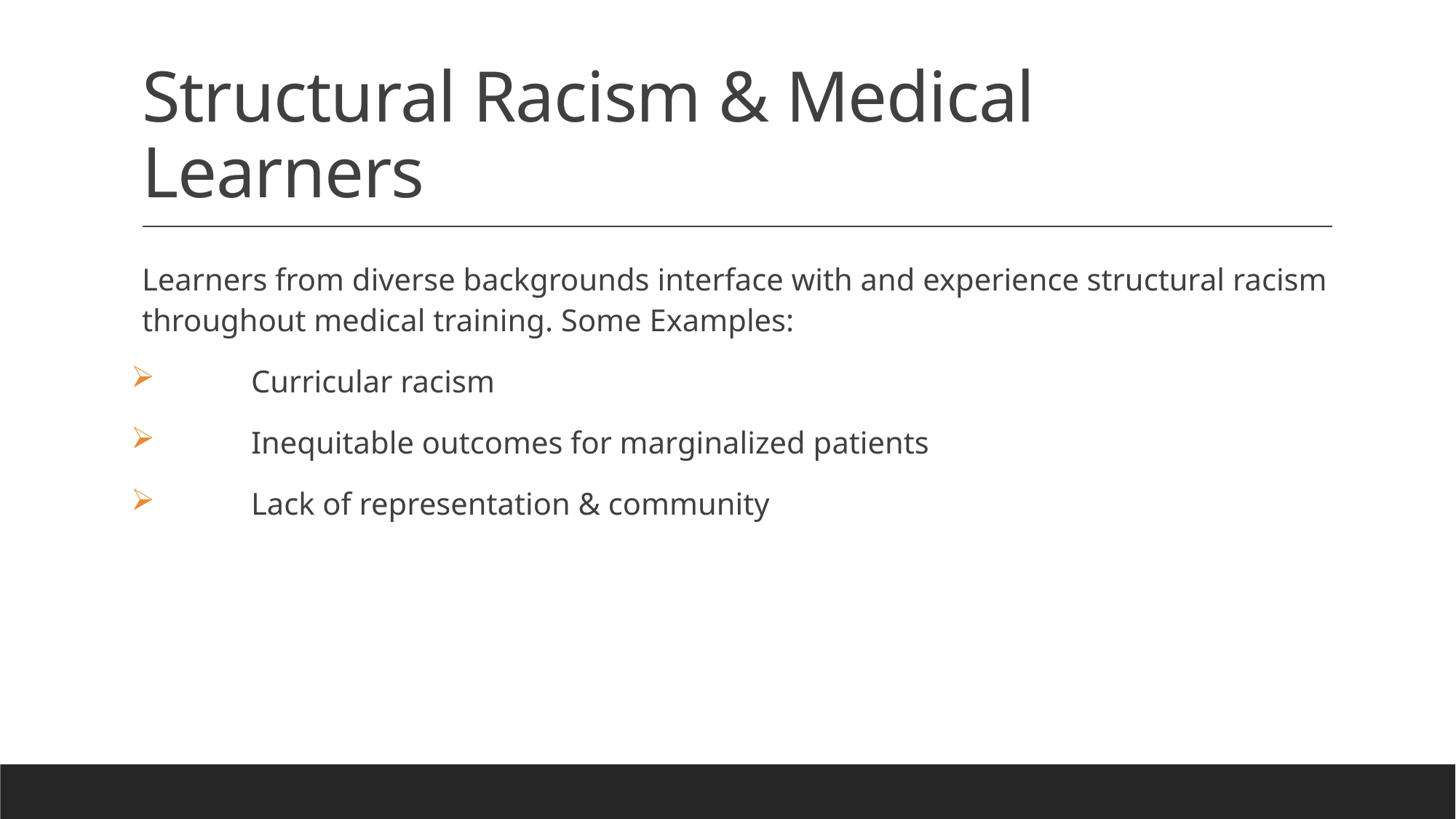

# Structural Racism & Medical Learners
Learners from diverse backgrounds interface with and experience structural racism throughout medical training. Some Examples:
	Curricular racism
	Inequitable outcomes for marginalized patients
	Lack of representation & community

## Slide 15
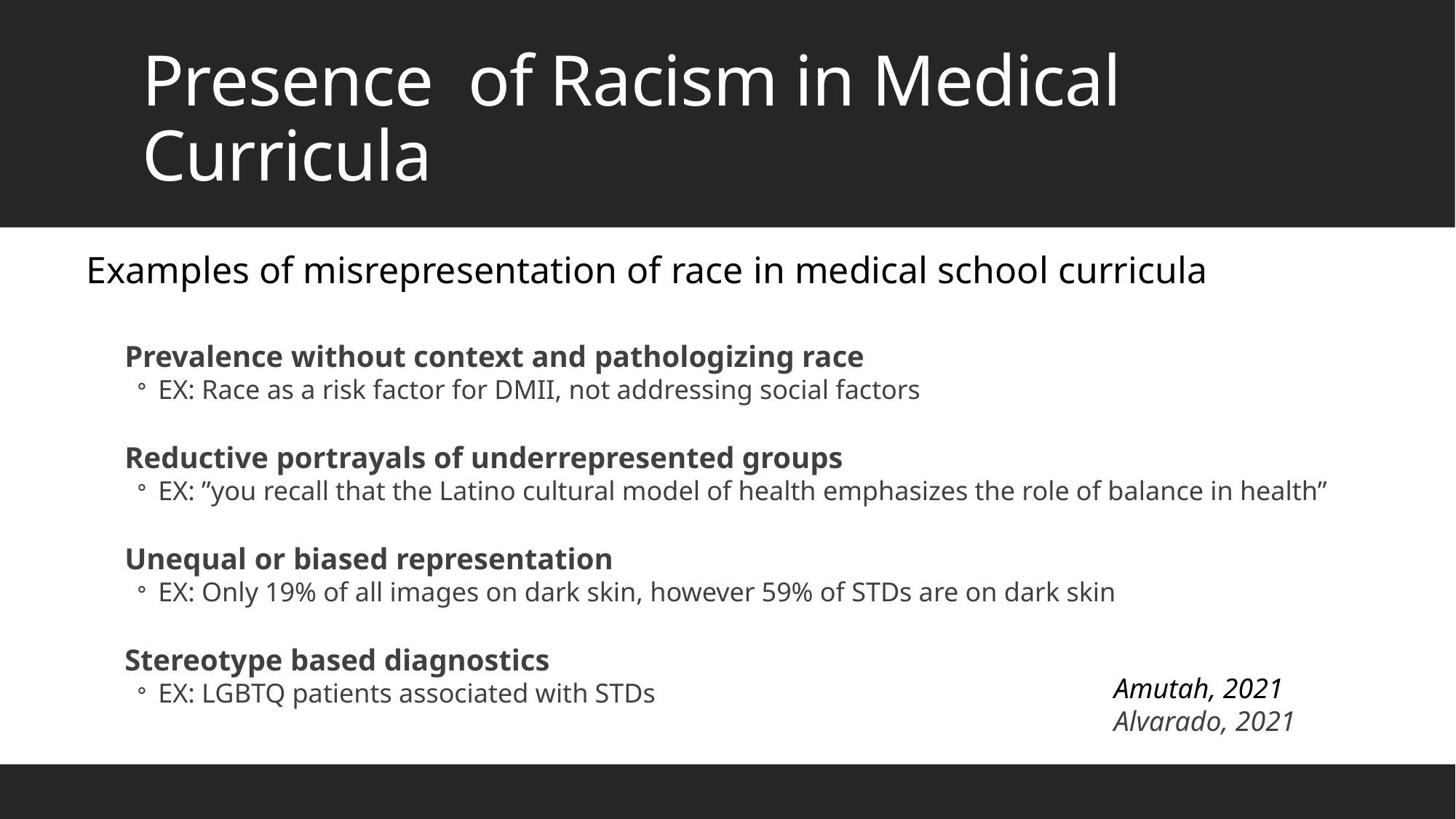

# Presence of Racism in Medical Curricula
Examples of misrepresentation of race in medical school curricula
Prevalence without context and pathologizing race
EX: Race as a risk factor for DMII, not addressing social factors
Reductive portrayals of underrepresented groups
EX: ”you recall that the Latino cultural model of health emphasizes the role of balance in health”
Unequal or biased representation
EX: Only 19% of all images on dark skin, however 59% of STDs are on dark skin
Stereotype based diagnostics
EX: LGBTQ patients associated with STDs
Amutah, 2021
Alvarado, 2021

## Slide 16
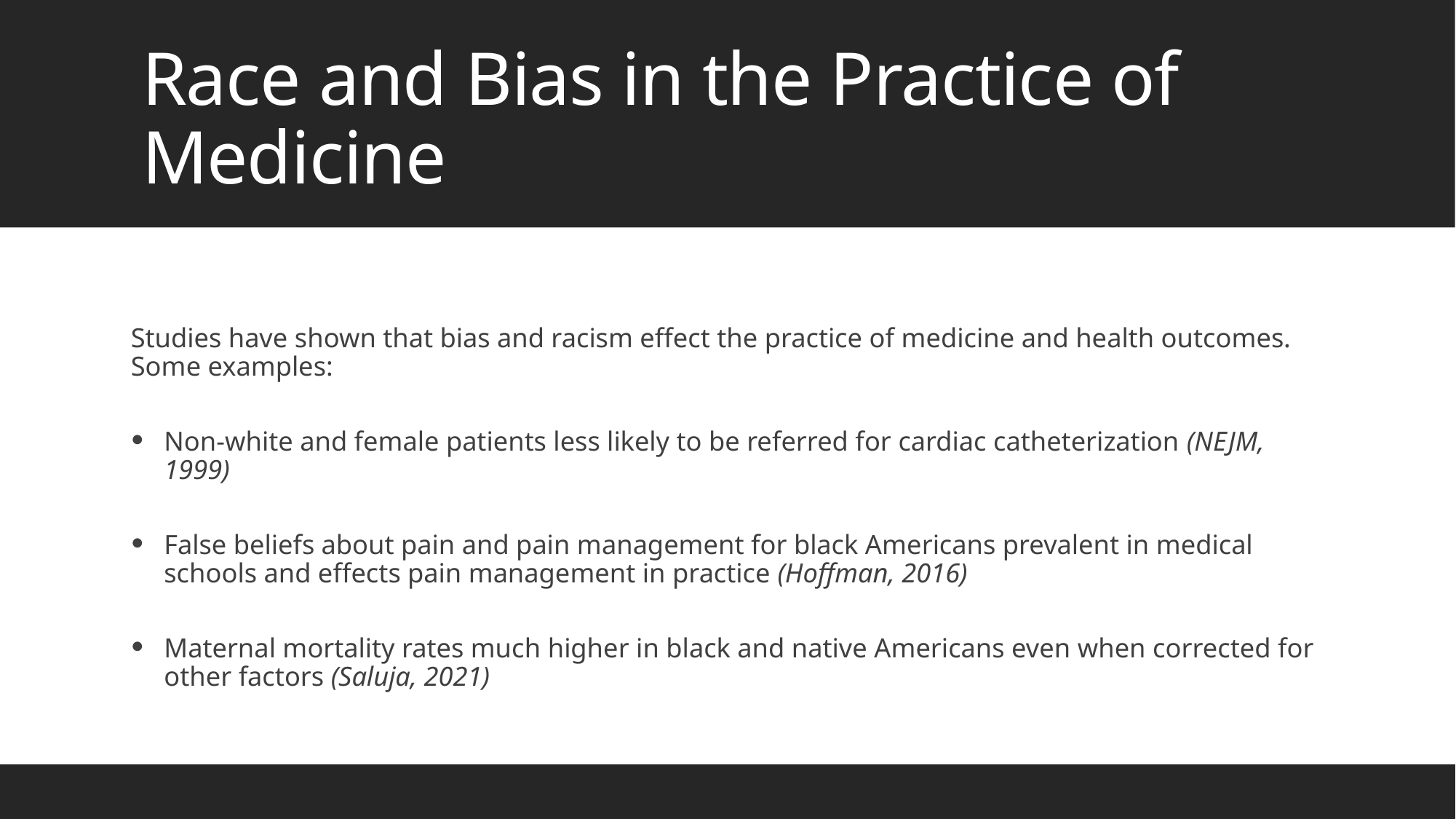

# Race and Bias in the Practice of Medicine
Studies have shown that bias and racism effect the practice of medicine and health outcomes. Some examples:
Non-white and female patients less likely to be referred for cardiac catheterization (NEJM, 1999)
False beliefs about pain and pain management for black Americans prevalent in medical schools and effects pain management in practice (Hoffman, 2016)
Maternal mortality rates much higher in black and native Americans even when corrected for other factors (Saluja, 2021)

## Slide 17
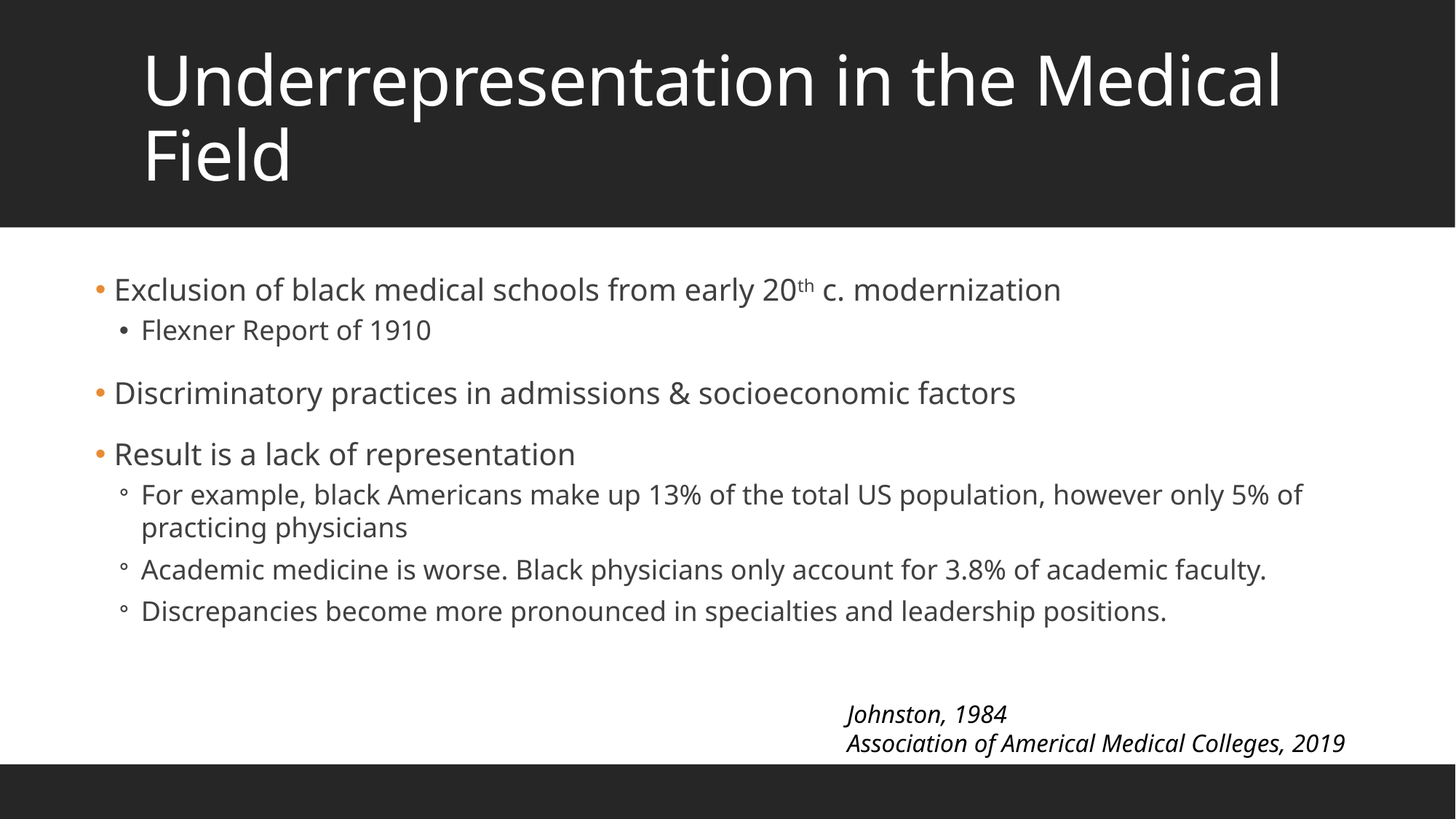

# Underrepresentation in the Medical Field
 Exclusion of black medical schools from early 20th c. modernization
Flexner Report of 1910
 Discriminatory practices in admissions & socioeconomic factors
 Result is a lack of representation
For example, black Americans make up 13% of the total US population, however only 5% of practicing physicians
Academic medicine is worse. Black physicians only account for 3.8% of academic faculty.
Discrepancies become more pronounced in specialties and leadership positions.
Johnston, 1984
Association of Americal Medical Colleges, 2019

## Slide 18
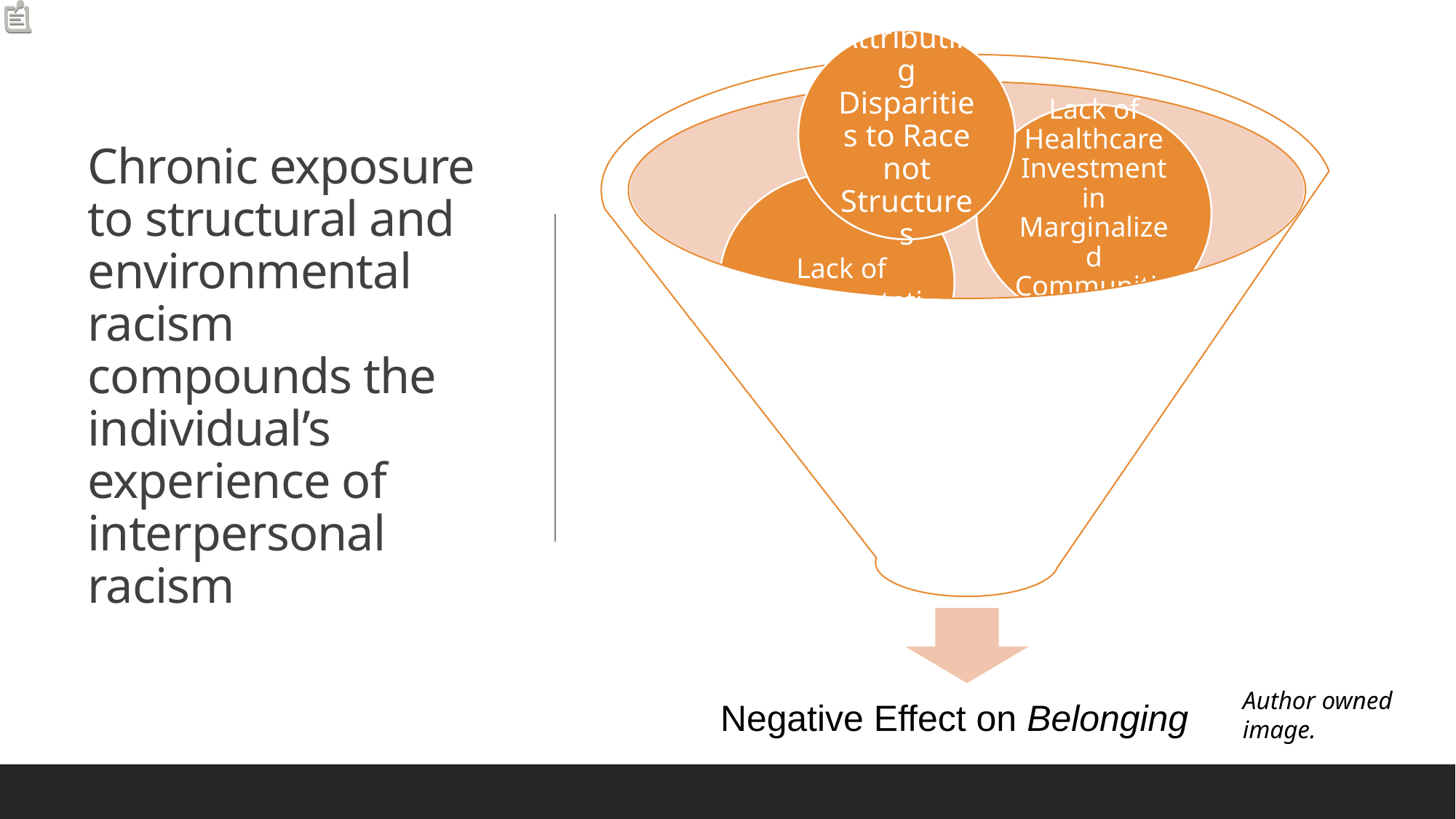

Attributing Disparities to Race not Structures
Lack of Healthcare Investment in Marginalized Communities
Failure to Address Appropriately in Med-Ed
Negative Effect on Belonging
# Chronic exposure to structural and environmental racism compounds the individual’s experience of interpersonal racism
Lack of Representation
Author owned image.

## Slide 19
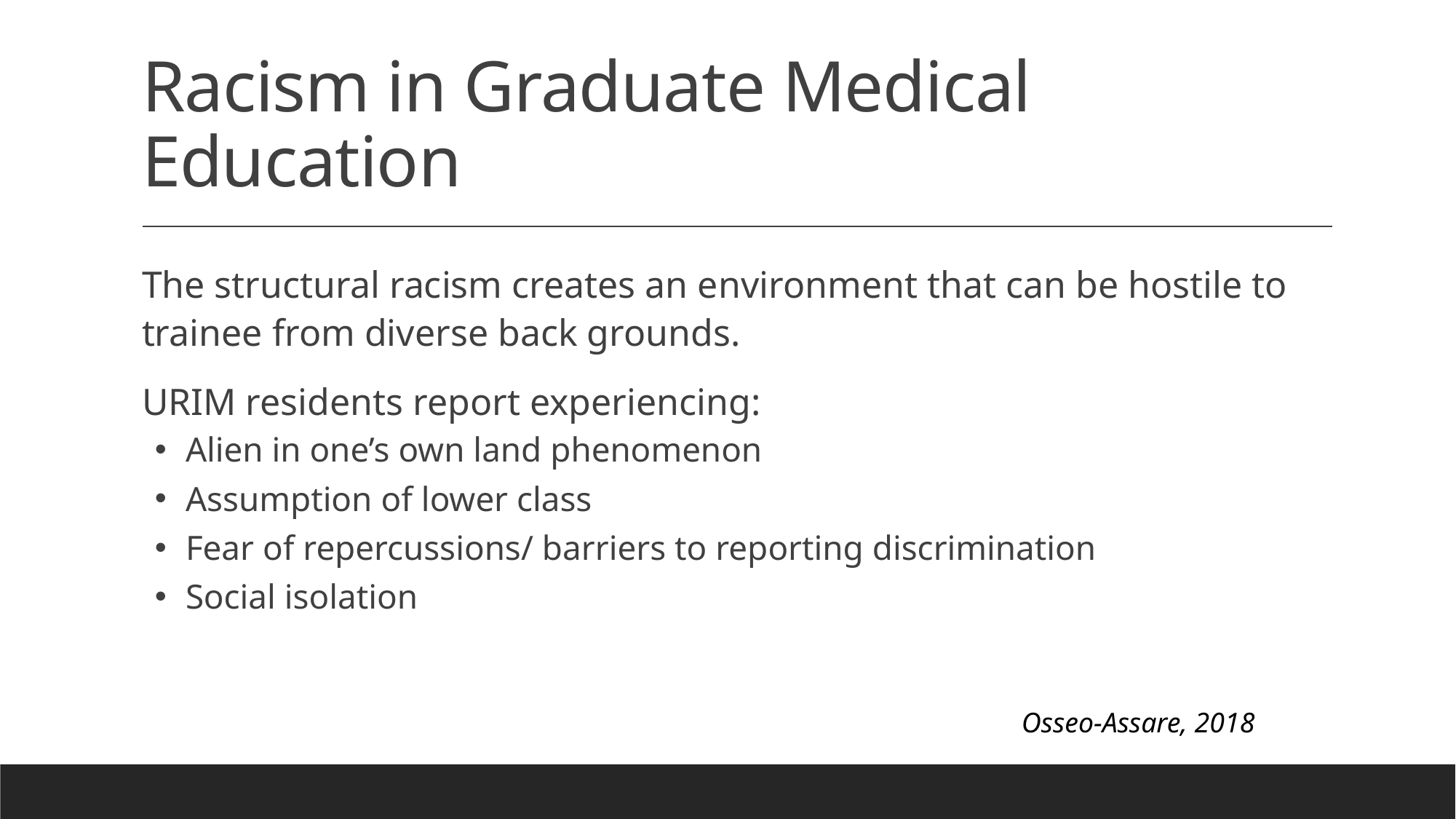

# Racism in Graduate Medical Education
The structural racism creates an environment that can be hostile to trainee from diverse back grounds.
URIM residents report experiencing:
 Alien in one’s own land phenomenon
 Assumption of lower class
 Fear of repercussions/ barriers to reporting discrimination
 Social isolation
Osseo-Assare, 2018

## Slide 20
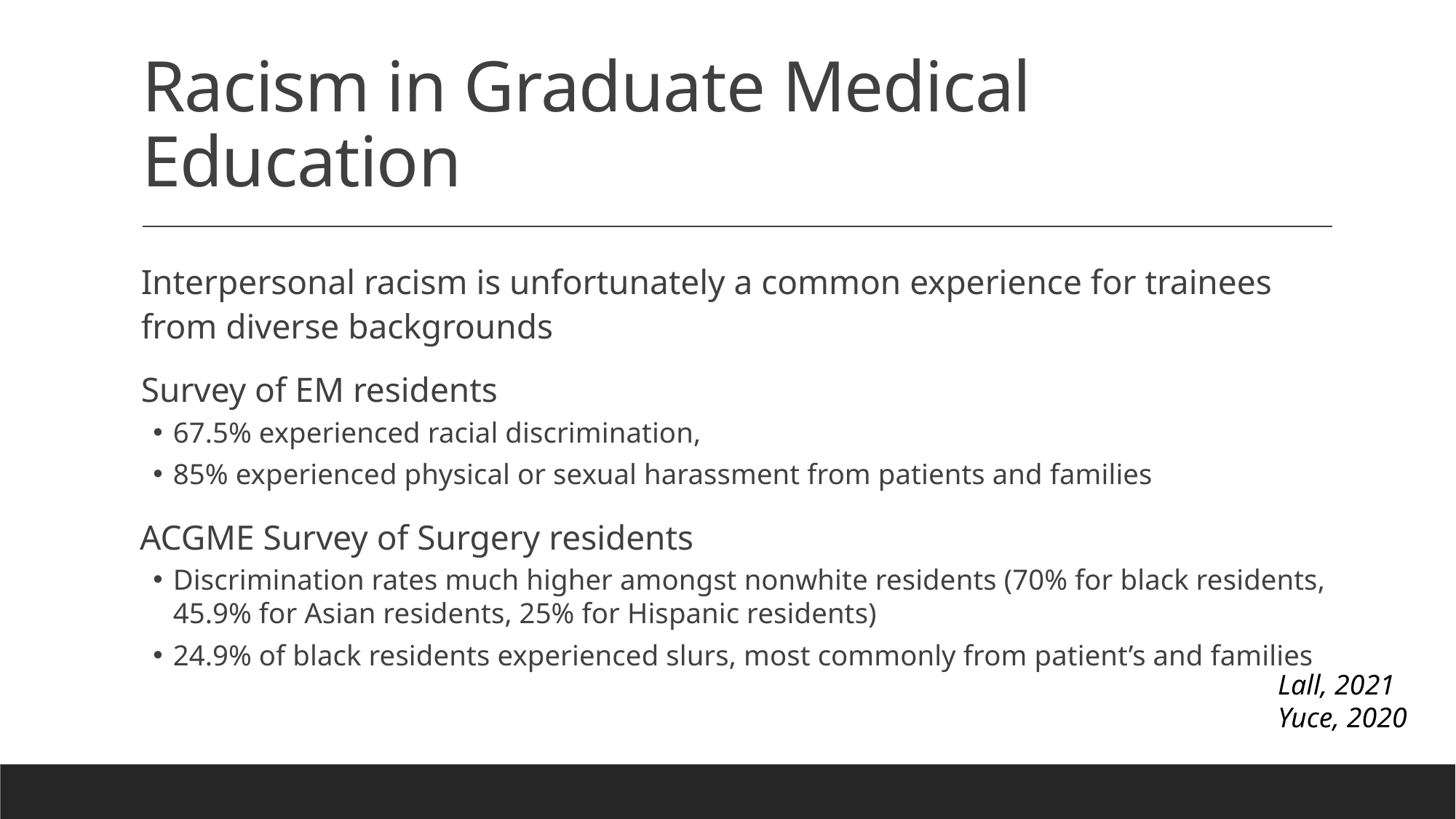

# Racism in Graduate Medical Education
Interpersonal racism is unfortunately a common experience for trainees from diverse backgrounds
Survey of EM residents
67.5% experienced racial discrimination,
85% experienced physical or sexual harassment from patients and families
 ACGME Survey of Surgery residents
Discrimination rates much higher amongst nonwhite residents (70% for black residents, 45.9% for Asian residents, 25% for Hispanic residents)
24.9% of black residents experienced slurs, most commonly from patient’s and families
Lall, 2021
Yuce, 2020

## Slide 21
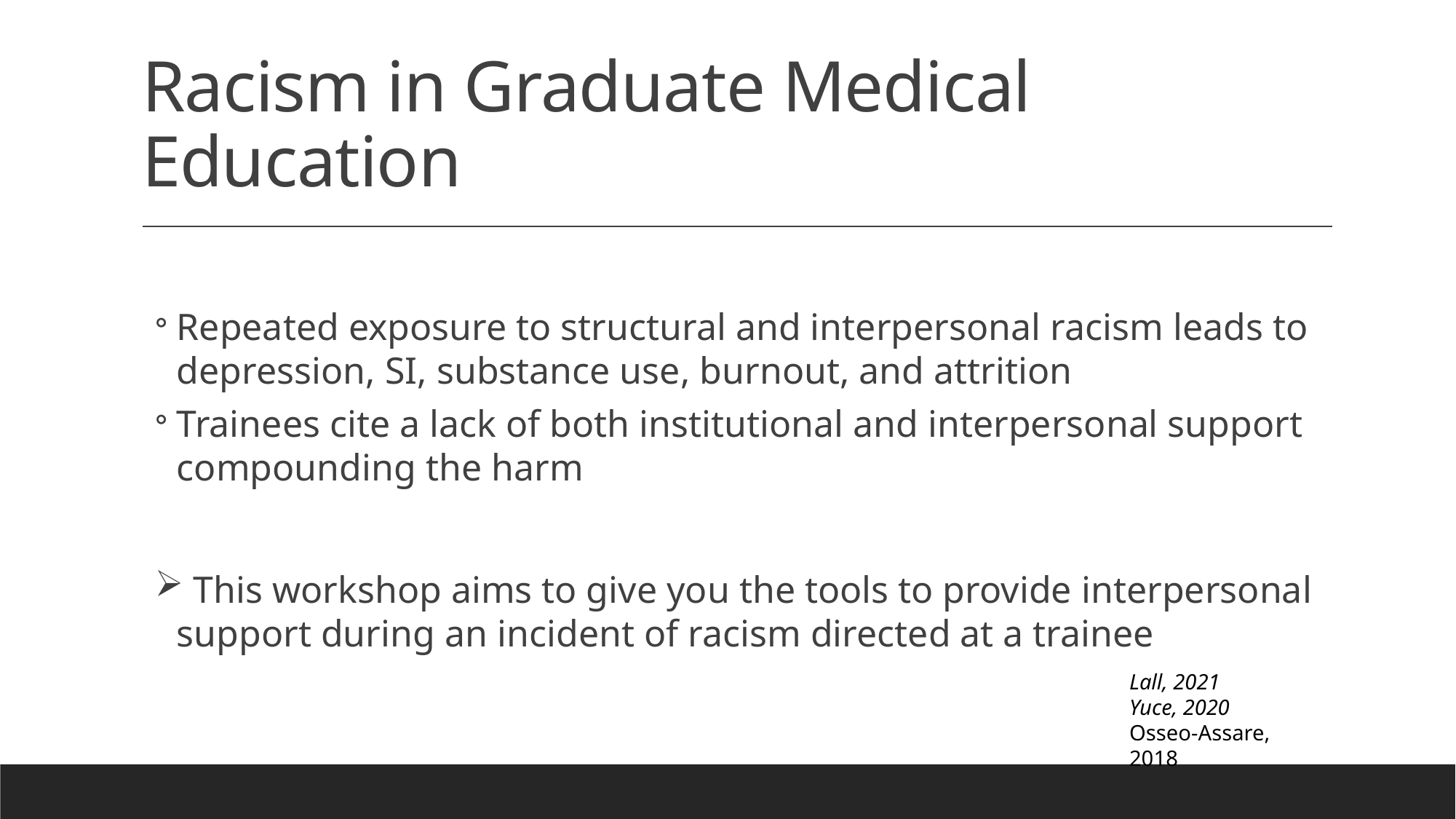

# Racism in Graduate Medical Education
Repeated exposure to structural and interpersonal racism leads to depression, SI, substance use, burnout, and attrition
Trainees cite a lack of both institutional and interpersonal support compounding the harm
 This workshop aims to give you the tools to provide interpersonal support during an incident of racism directed at a trainee
Lall, 2021
Yuce, 2020
Osseo-Assare, 2018

## Slide 22
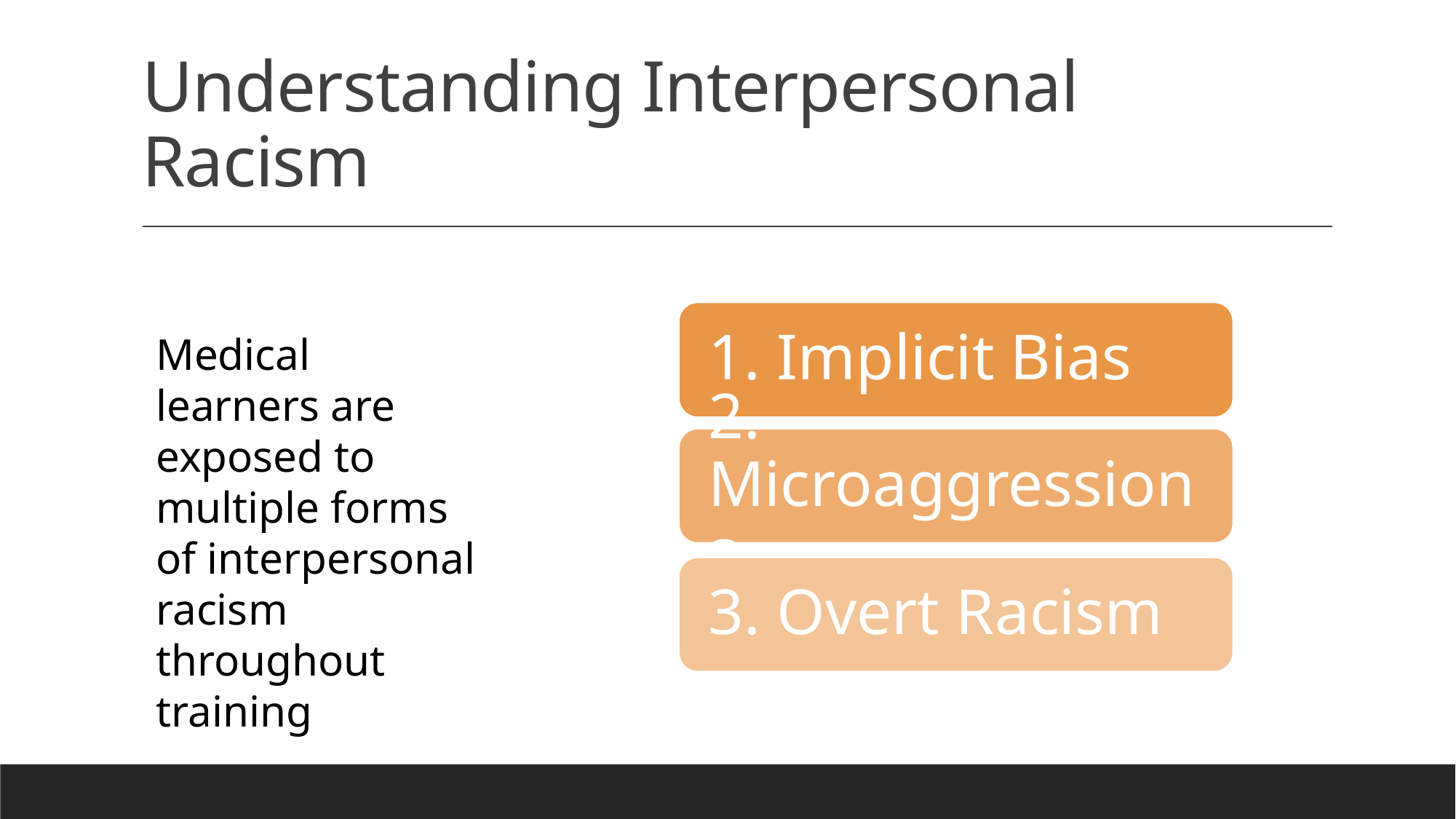

# Understanding Interpersonal Racism
Medical learners are exposed to multiple forms of interpersonal racism throughout training

## Slide 23
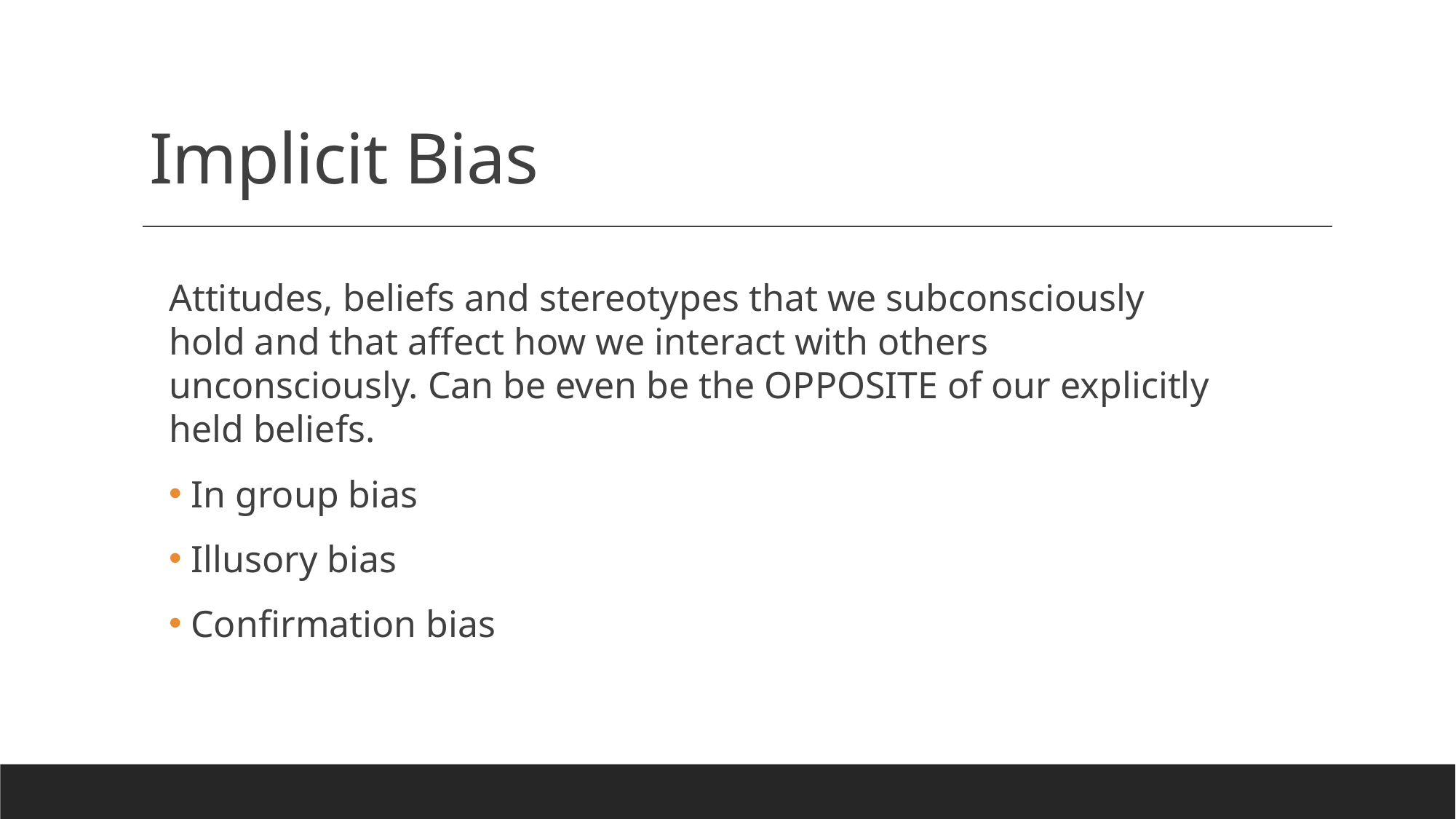

# Implicit Bias
Attitudes, beliefs and stereotypes that we subconsciously hold and that affect how we interact with others unconsciously. Can be even be the OPPOSITE of our explicitly held beliefs.
 In group bias
 Illusory bias
 Confirmation bias

## Slide 24
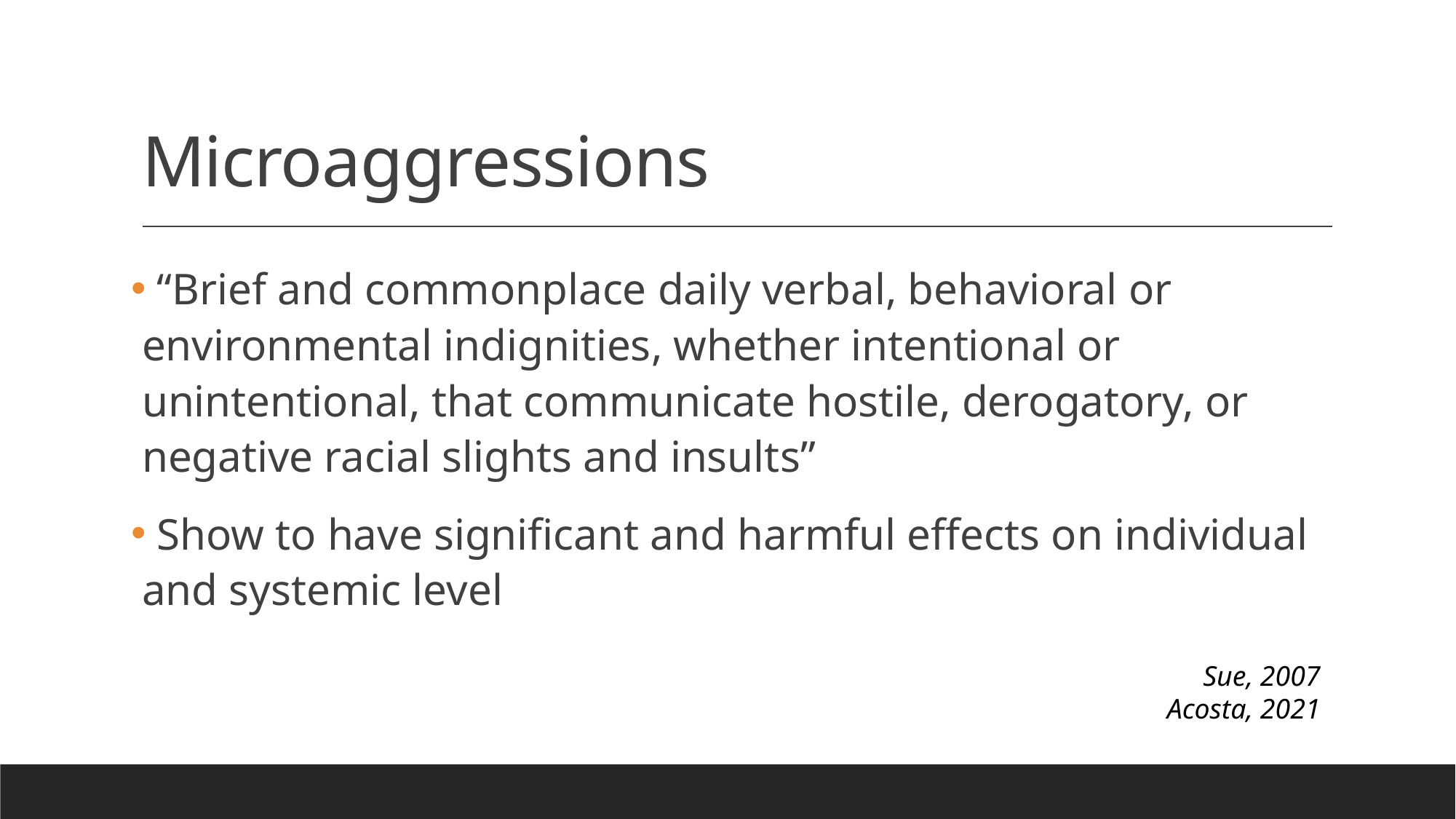

# Microaggressions
 “Brief and commonplace daily verbal, behavioral or environmental indignities, whether intentional or unintentional, that communicate hostile, derogatory, or negative racial slights and insults”
 Show to have significant and harmful effects on individual and systemic level
Sue, 2007
Acosta, 2021

## Slide 25
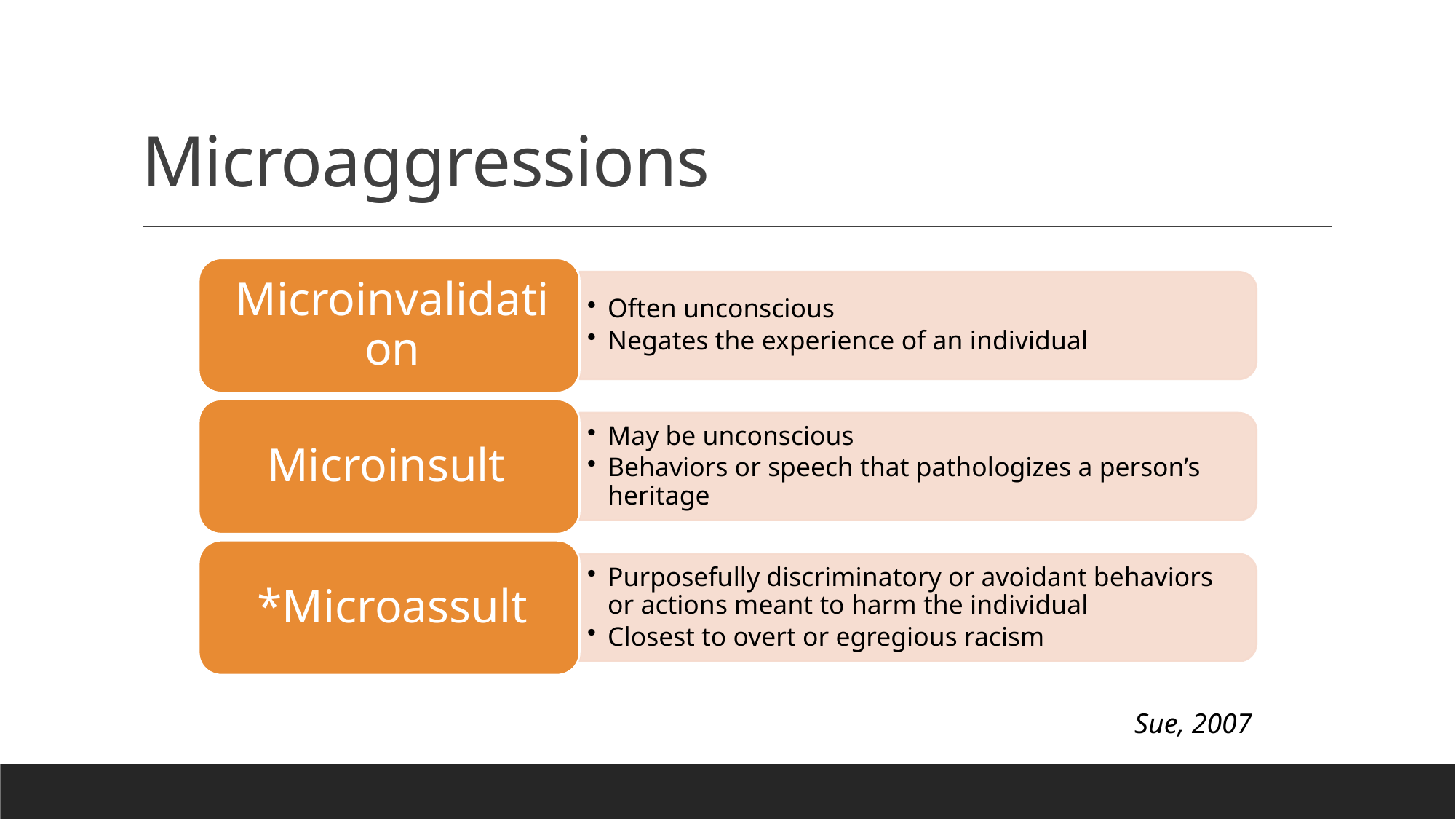

# Microaggressions
Sue, 2007

## Slide 26
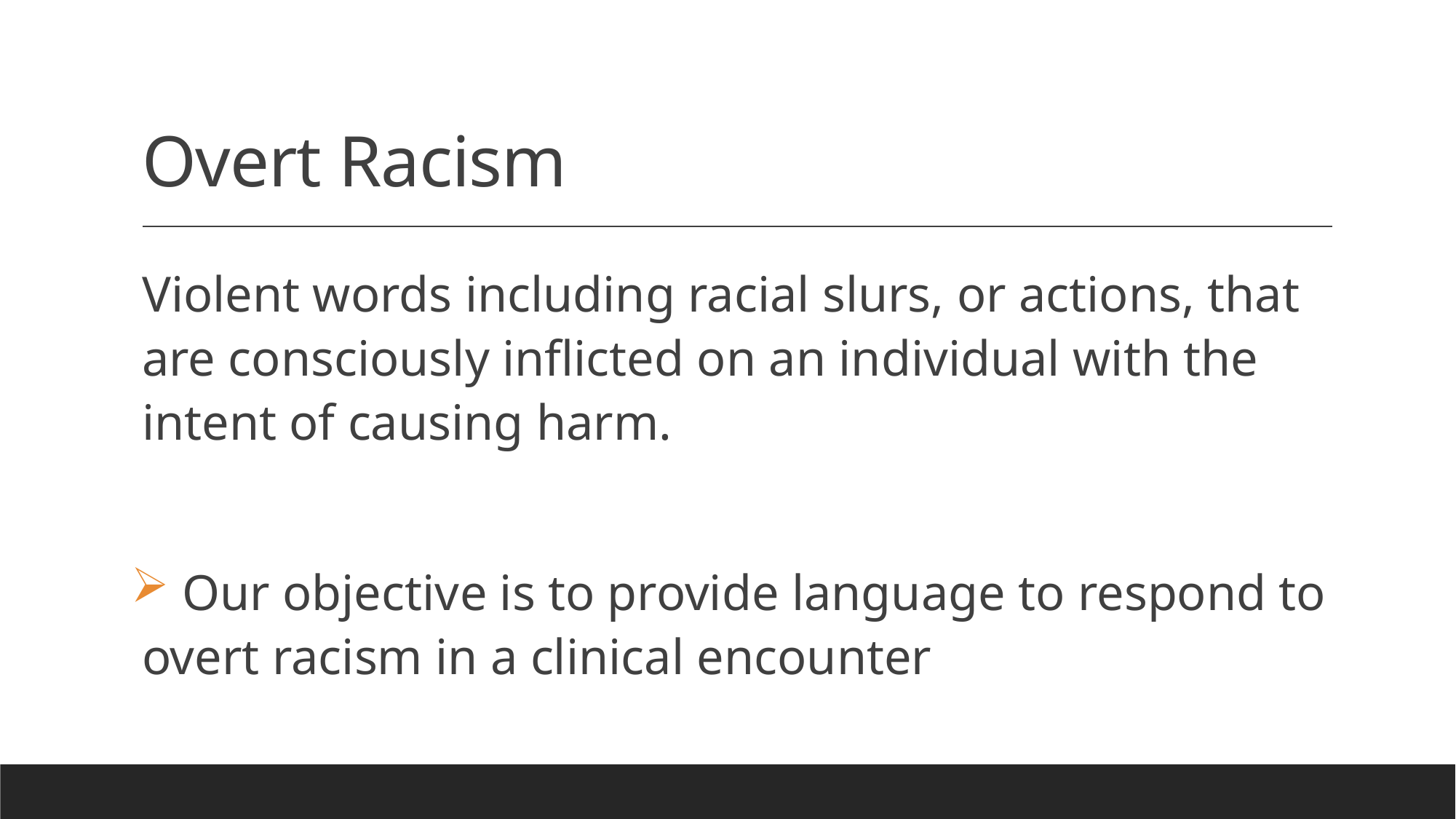

# Overt Racism
Violent words including racial slurs, or actions, that are consciously inflicted on an individual with the intent of causing harm.
 Our objective is to provide language to respond to overt racism in a clinical encounter

## Slide 27
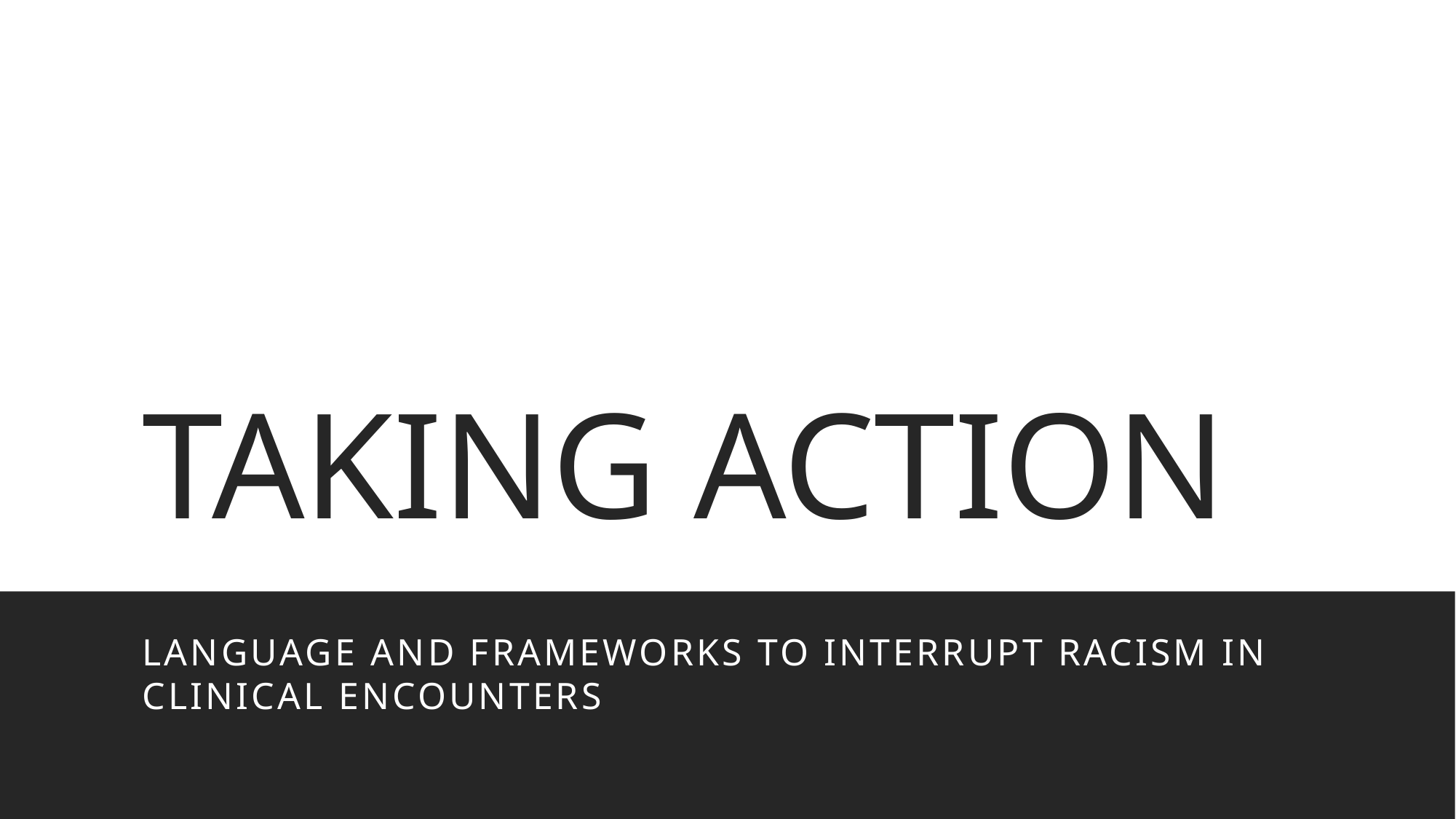

# TAKING ACTION
Language and frameworks to interrupt Racism in clinical encounters

## Slide 28
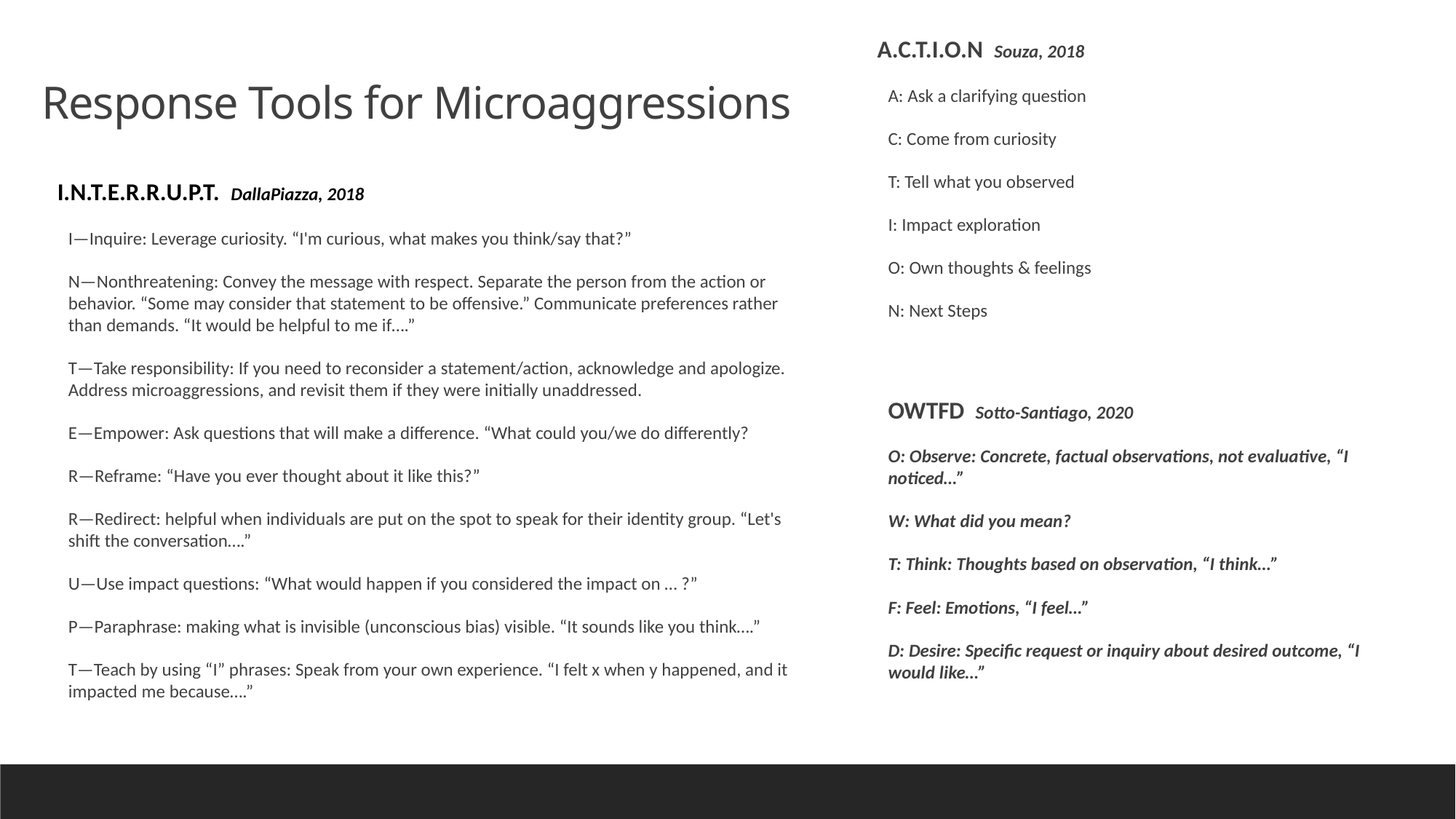

Response Tools for Microaggressions
A.C.T.I.O.N  Souza, 2018
A: Ask a clarifying question
C: Come from curiosity
T: Tell what you observed
I: Impact exploration
O: Own thoughts & feelings
N: Next Steps
I.N.T.E.R.R.U.P.T.  DallaPiazza, 2018
I—Inquire: Leverage curiosity. “I'm curious, what makes you think/say that?”
N—Nonthreatening: Convey the message with respect. Separate the person from the action or behavior. “Some may consider that statement to be offensive.” Communicate preferences rather than demands. “It would be helpful to me if….”
T—Take responsibility: If you need to reconsider a statement/action, acknowledge and apologize. Address microaggressions, and revisit them if they were initially unaddressed.
E—Empower: Ask questions that will make a difference. “What could you/we do differently?
R—Reframe: “Have you ever thought about it like this?”
R—Redirect: helpful when individuals are put on the spot to speak for their identity group. “Let's shift the conversation….”
U—Use impact questions: “What would happen if you considered the impact on … ?”
P—Paraphrase: making what is invisible (unconscious bias) visible. “It sounds like you think….”
T—Teach by using “I” phrases: Speak from your own experience. “I felt x when y happened, and it impacted me because….”
OWTFD  Sotto-Santiago, 2020
O: Observe: Concrete, factual observations, not evaluative, “I noticed…”
W: What did you mean?
T: Think: Thoughts based on observation, “I think…”
F: Feel: Emotions, “I feel…”
D: Desire: Specific request or inquiry about desired outcome, “I would like…”

## Slide 29
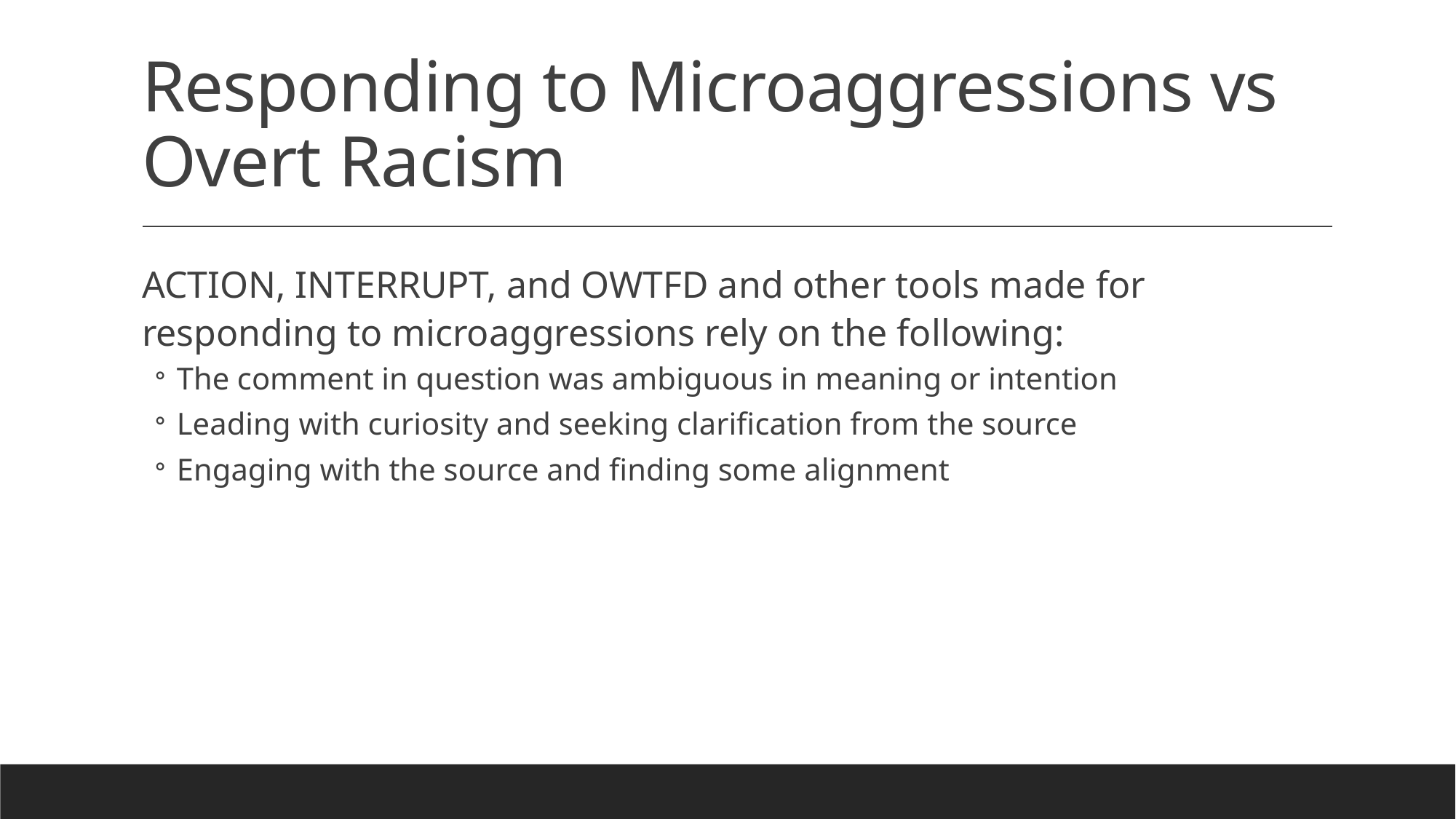

# Responding to Microaggressions vs Overt Racism
ACTION, INTERRUPT, and OWTFD and other tools made for responding to microaggressions rely on the following:
The comment in question was ambiguous in meaning or intention
Leading with curiosity and seeking clarification from the source
Engaging with the source and finding some alignment

## Slide 30
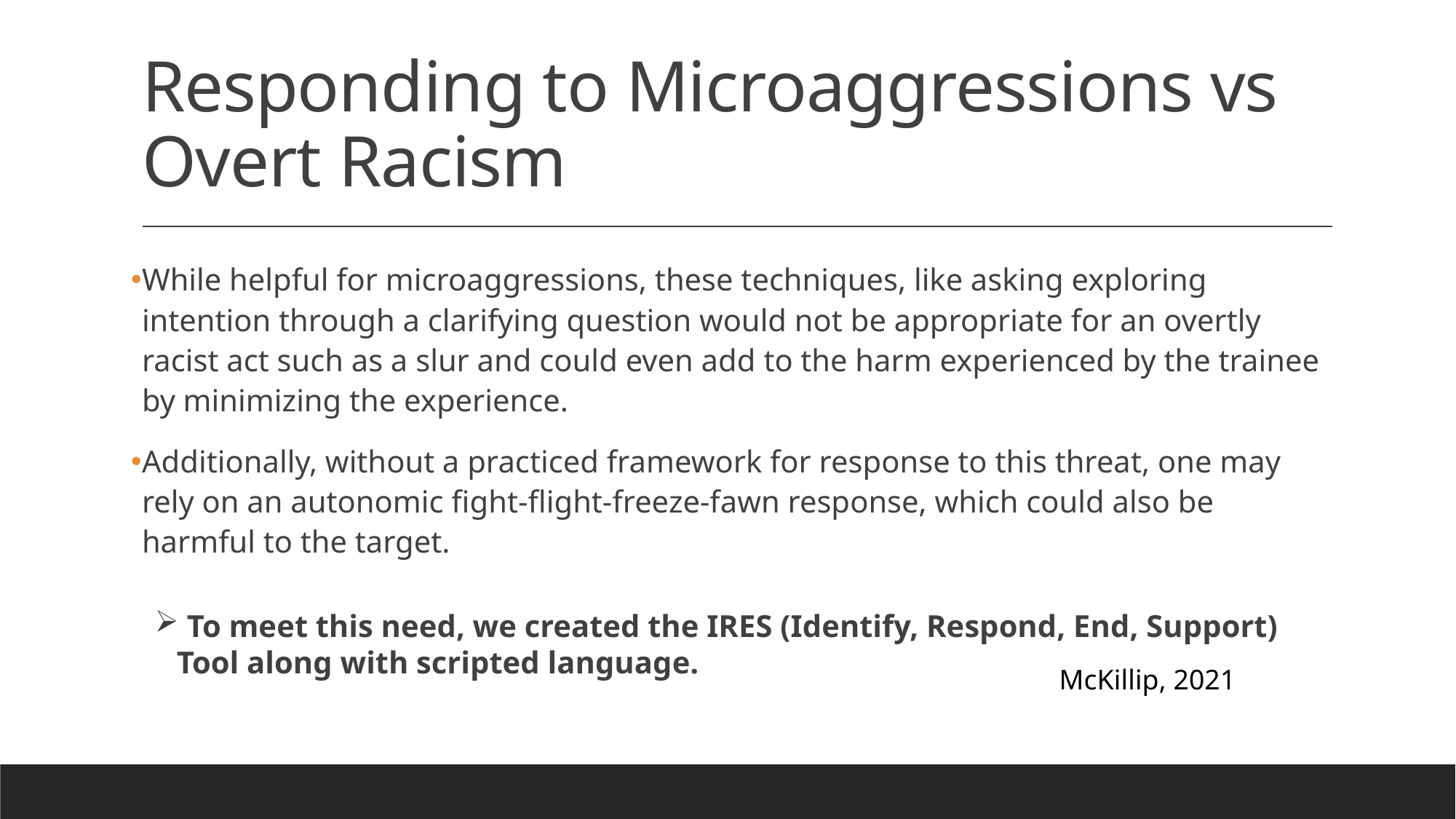

# Responding to Microaggressions vs Overt Racism
While helpful for microaggressions, these techniques, like asking exploring intention through a clarifying question would not be appropriate for an overtly racist act such as a slur and could even add to the harm experienced by the trainee by minimizing the experience.
Additionally, without a practiced framework for response to this threat, one may rely on an autonomic fight-flight-freeze-fawn response, which could also be harmful to the target.
 To meet this need, we created the IRES (Identify, Respond, End, Support) Tool along with scripted language.
McKillip, 2021

## Slide 31
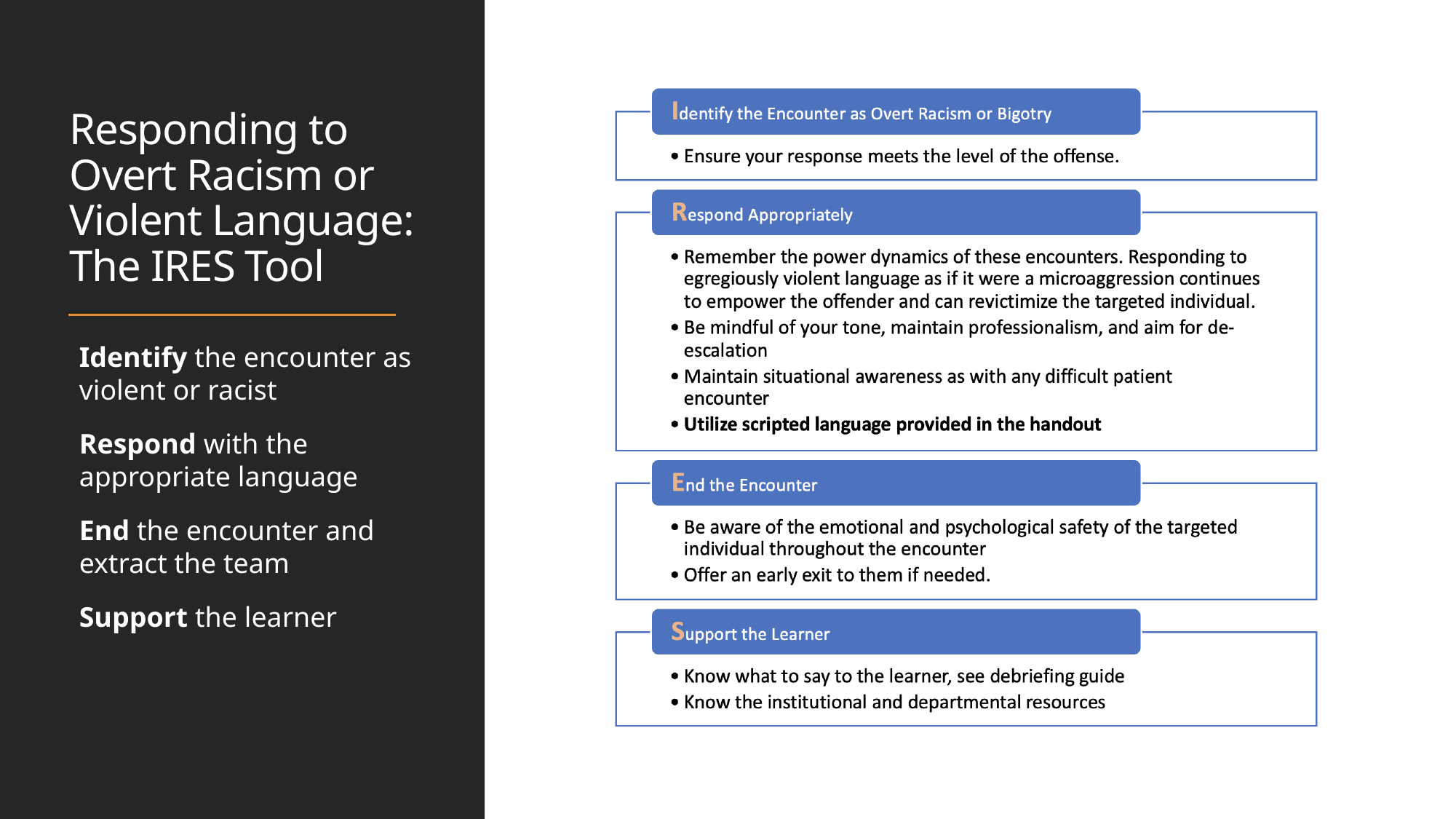

# Responding to Overt Racism or Violent Language: The IRES Tool
Identify the encounter as violent or racist
Respond with the appropriate language
End the encounter and extract the team
Support the learner

## Slide 32
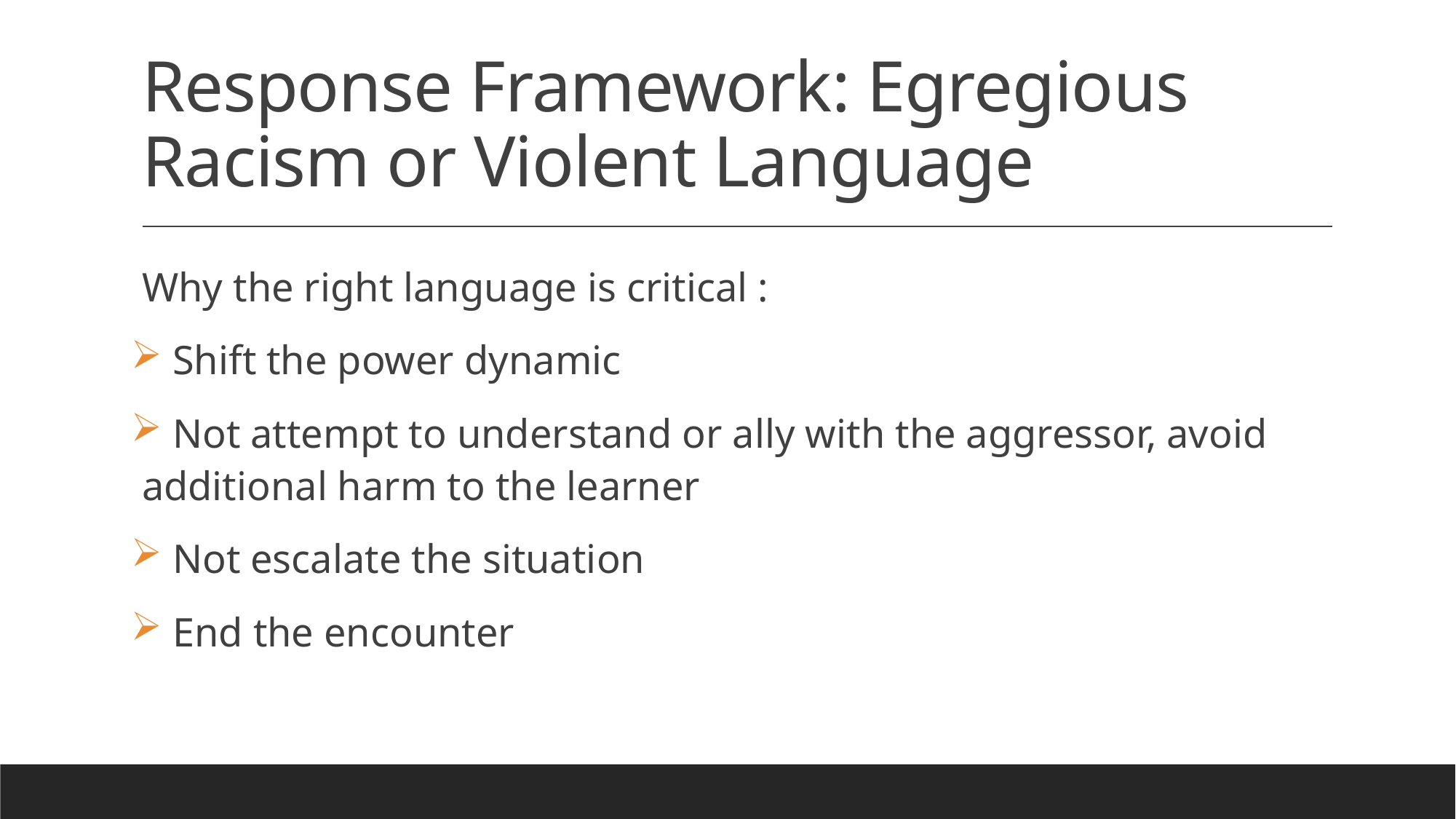

# Response Framework: Egregious Racism or Violent Language
Why the right language is critical :
 Shift the power dynamic
 Not attempt to understand or ally with the aggressor, avoid additional harm to the learner
 Not escalate the situation
 End the encounter

## Slide 33
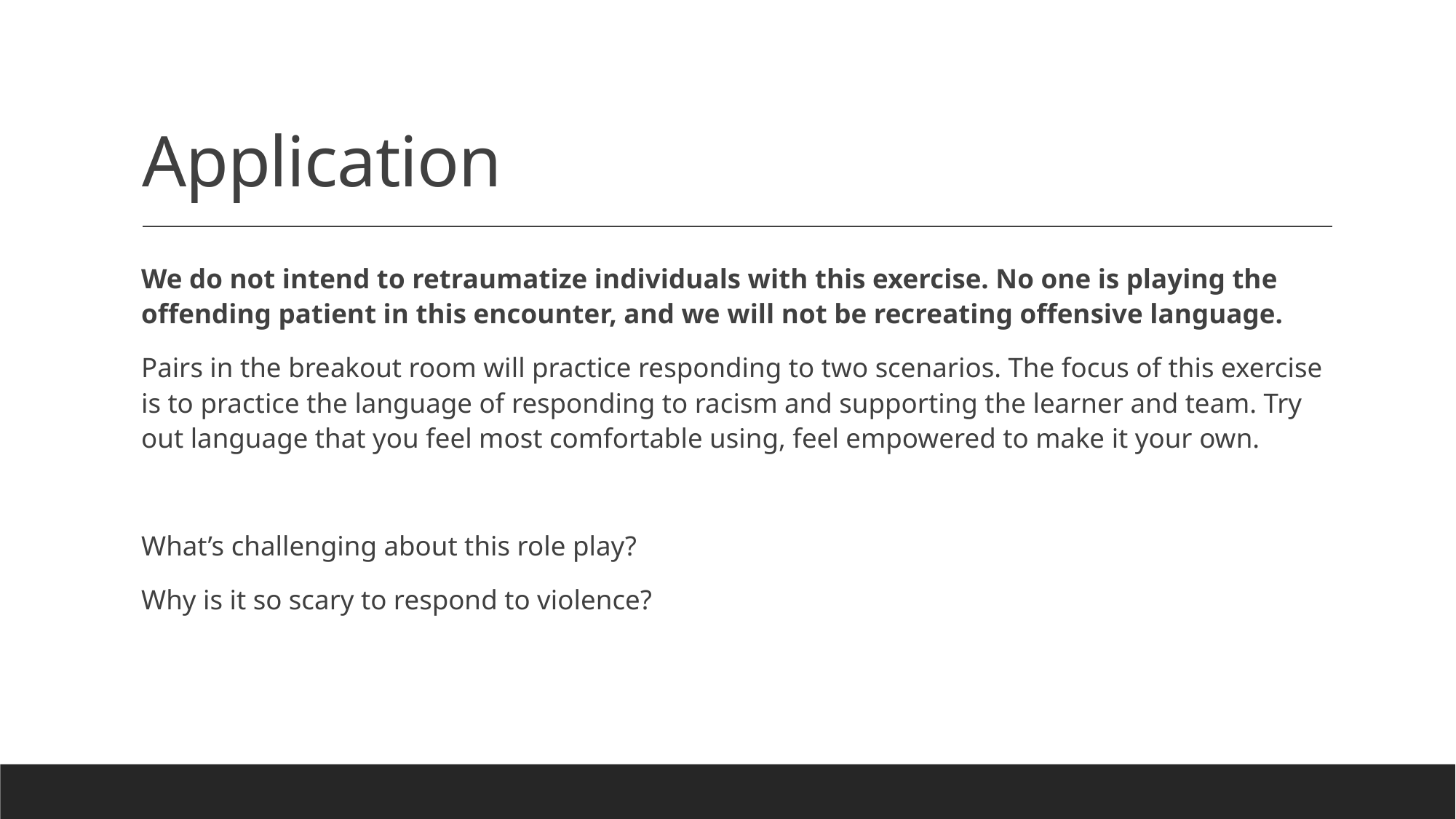

# Application
We do not intend to retraumatize individuals with this exercise. No one is playing the offending patient in this encounter, and we will not be recreating offensive language.
Pairs in the breakout room will practice responding to two scenarios. The focus of this exercise is to practice the language of responding to racism and supporting the learner and team. Try out language that you feel most comfortable using, feel empowered to make it your own.
What’s challenging about this role play?
Why is it so scary to respond to violence?

## Slide 34
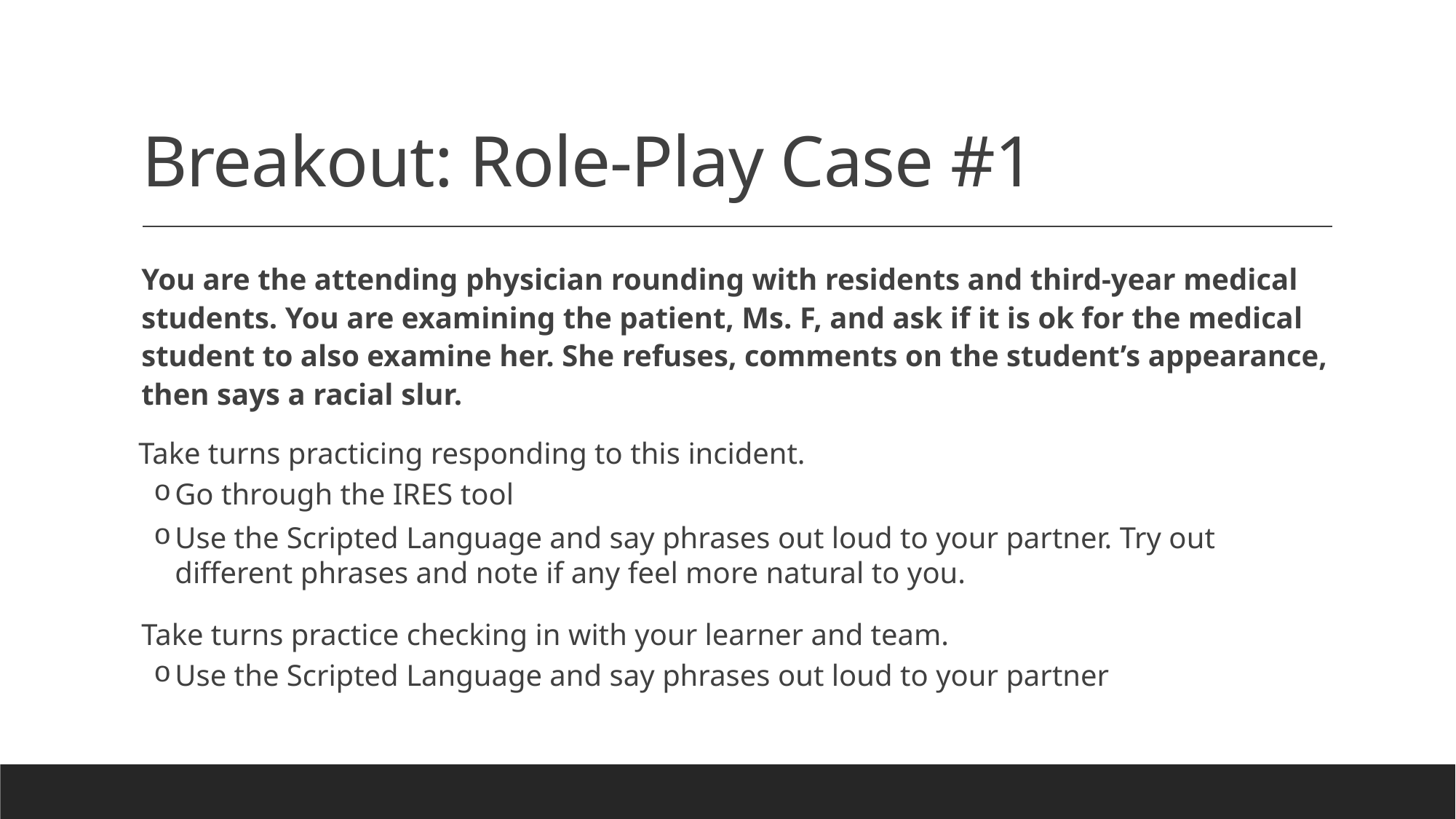

# Breakout: Role-Play Case #1
You are the attending physician rounding with residents and third-year medical students. You are examining the patient, Ms. F, and ask if it is ok for the medical student to also examine her. She refuses, comments on the student’s appearance, then says a racial slur.
 Take turns practicing responding to this incident.
Go through the IRES tool
Use the Scripted Language and say phrases out loud to your partner. Try out different phrases and note if any feel more natural to you.
Take turns practice checking in with your learner and team.
Use the Scripted Language and say phrases out loud to your partner

## Slide 35
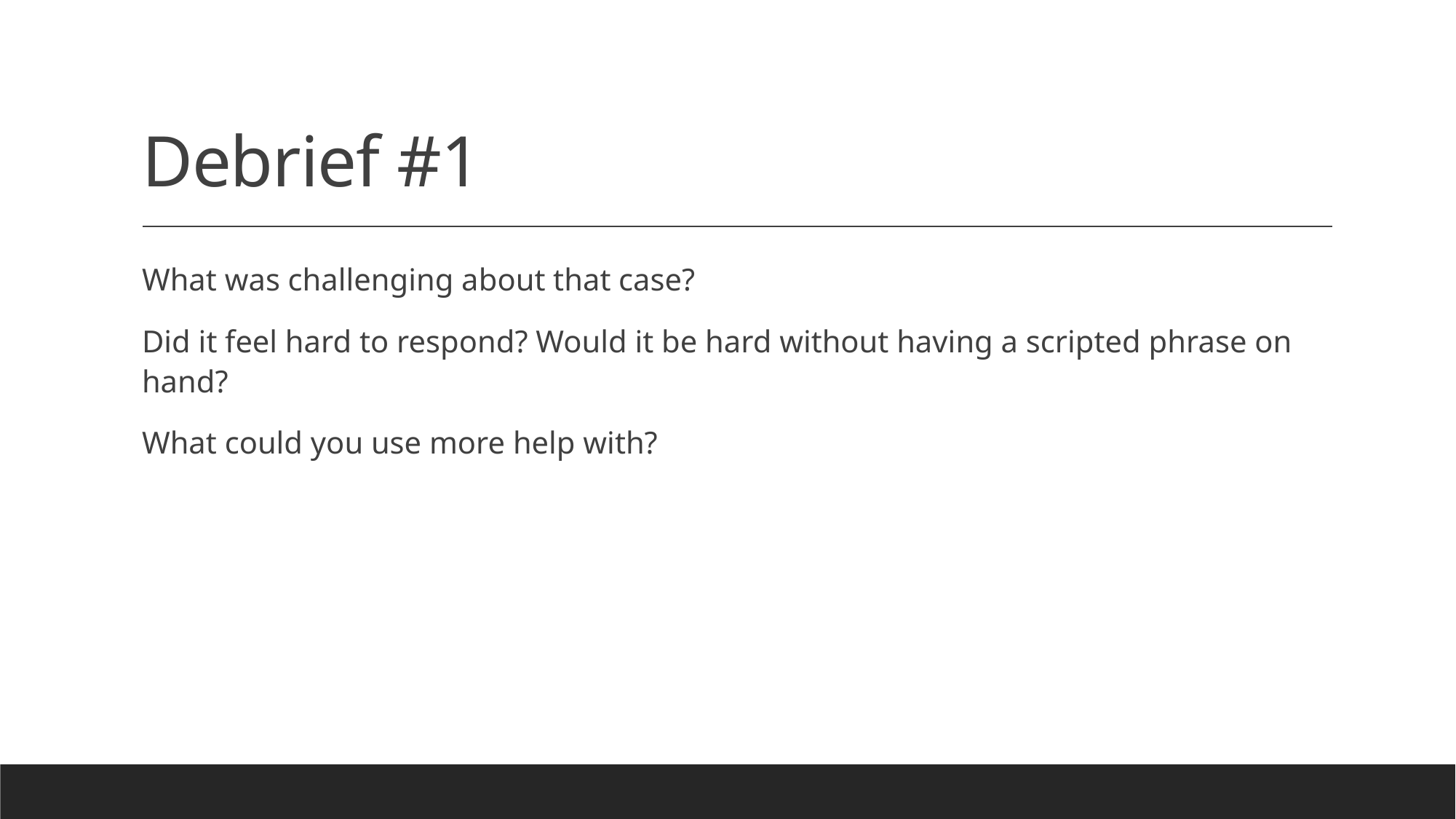

# Debrief #1
What was challenging about that case?
Did it feel hard to respond? Would it be hard without having a scripted phrase on hand?
What could you use more help with?

## Slide 36
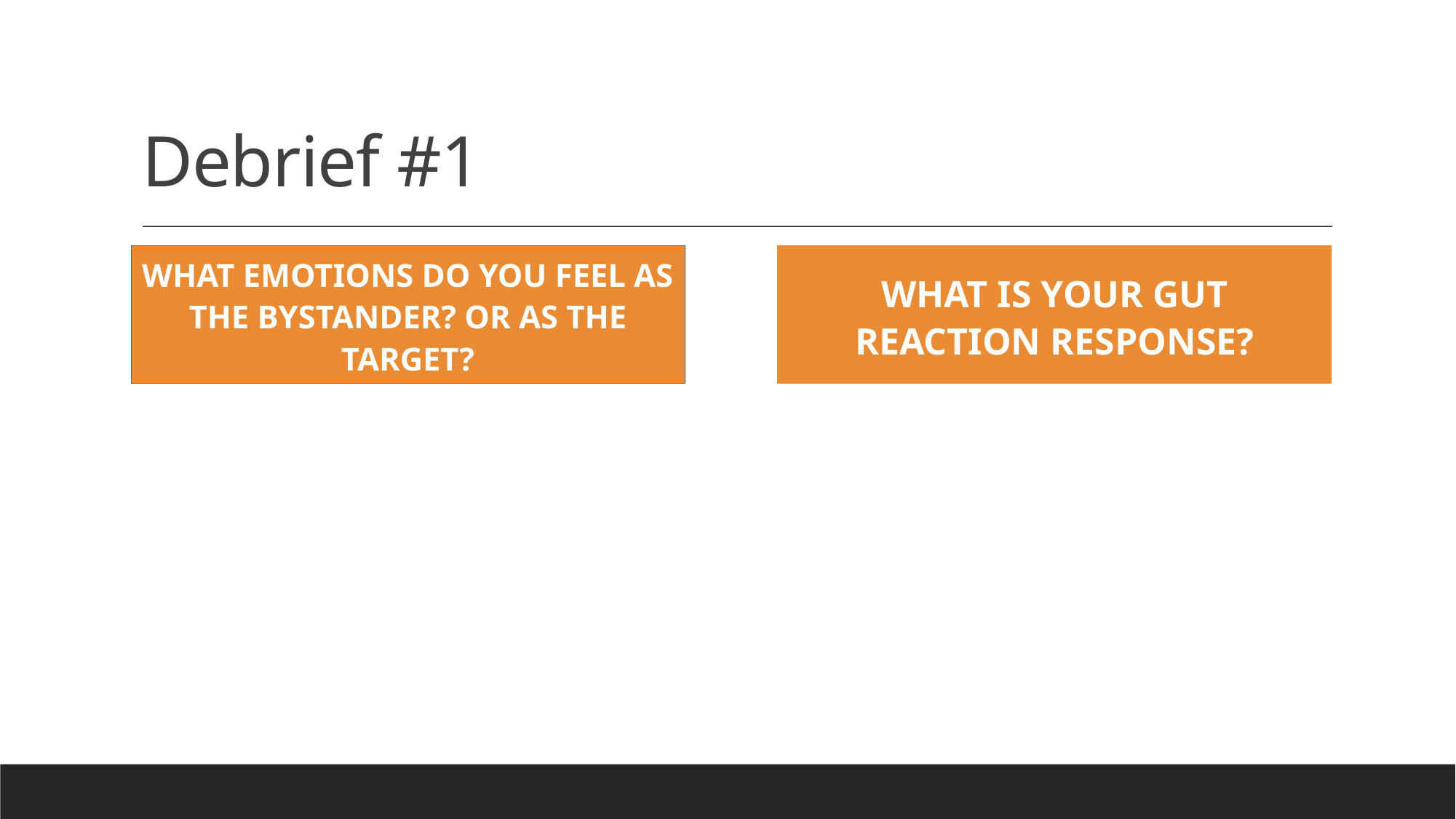

# Debrief #1
What emotions do you feel as the bystander? Or as the target?
What is your gut reaction response?

## Slide 37
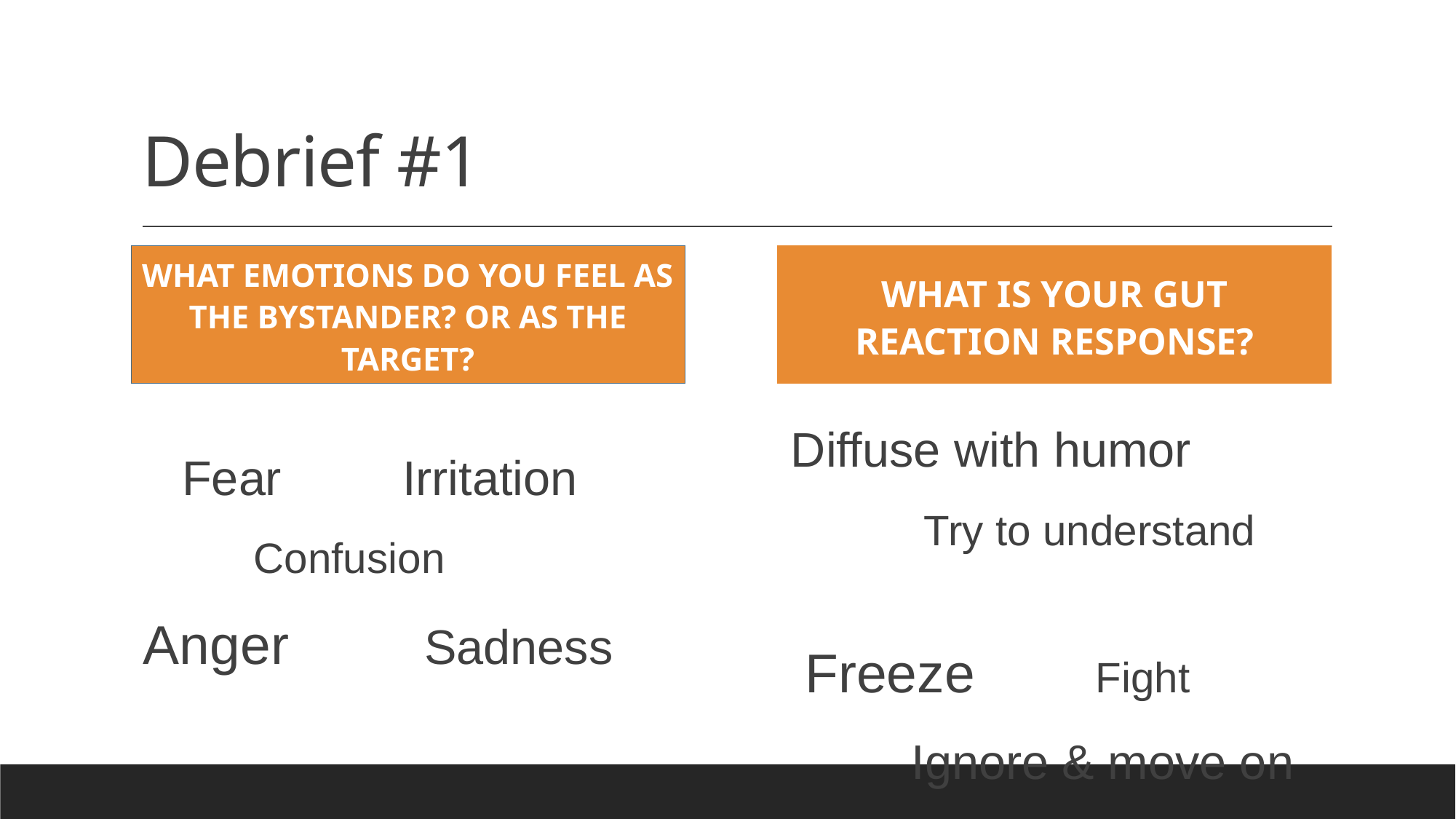

# Debrief #1
What emotions do you feel as the bystander? Or as the target?
What is your gut reaction response?
 Diffuse with humor
 Try to understand
 Freeze Fight
 Ignore & move on
 Fear Irritation
 Confusion
Anger Sadness

## Slide 38
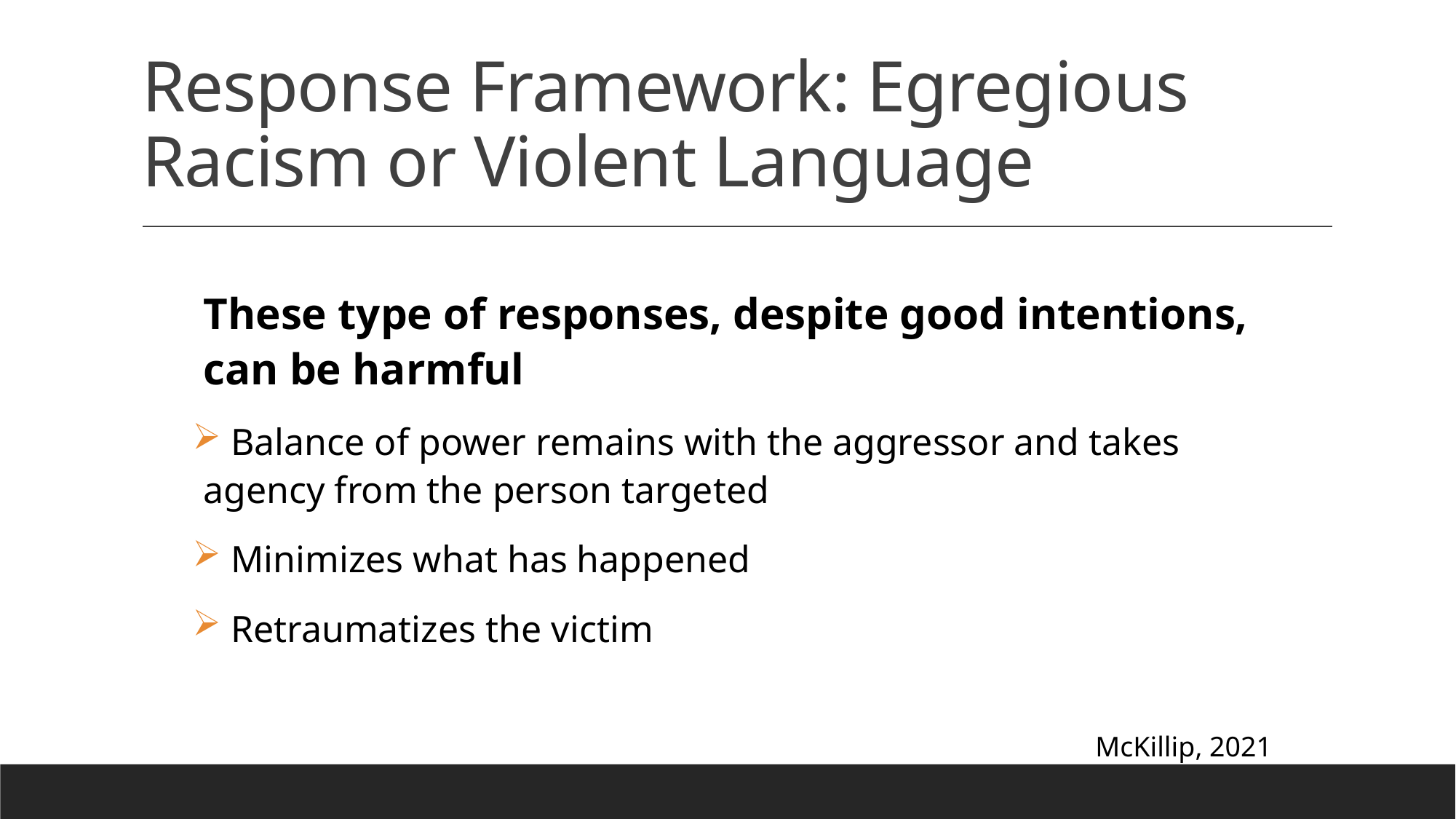

# Response Framework: Egregious Racism or Violent Language
These type of responses, despite good intentions, can be harmful
 Balance of power remains with the aggressor and takes agency from the person targeted
 Minimizes what has happened
 Retraumatizes the victim
McKillip, 2021

## Slide 39
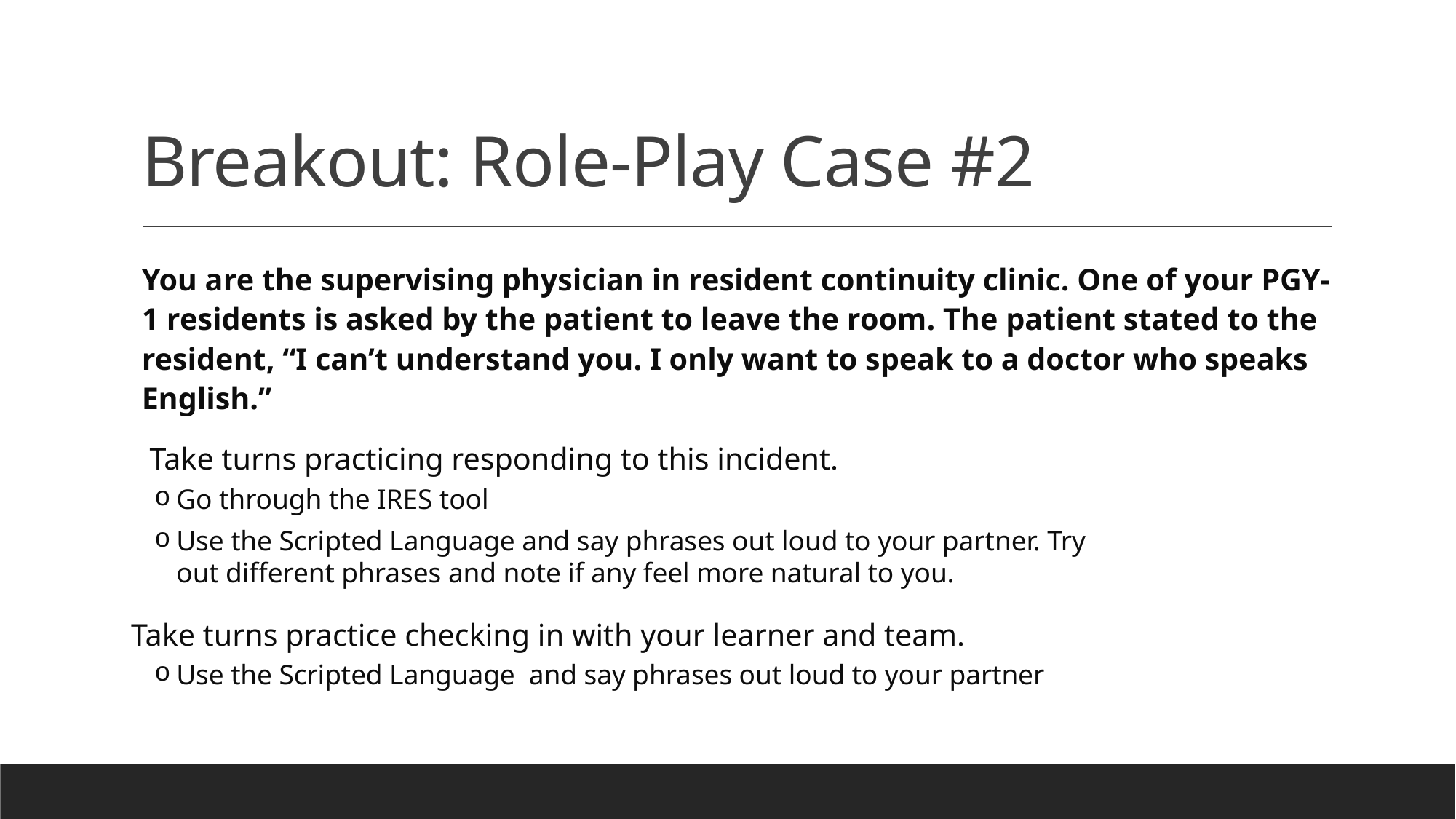

# Breakout: Role-Play Case #2
You are the supervising physician in resident continuity clinic. One of your PGY-1 residents is asked by the patient to leave the room. The patient stated to the resident, “I can’t understand you. I only want to speak to a doctor who speaks English.”
 Take turns practicing responding to this incident.
Go through the IRES tool
Use the Scripted Language and say phrases out loud to your partner. Try out different phrases and note if any feel more natural to you.
Take turns practice checking in with your learner and team.
Use the Scripted Language  and say phrases out loud to your partner

## Slide 40
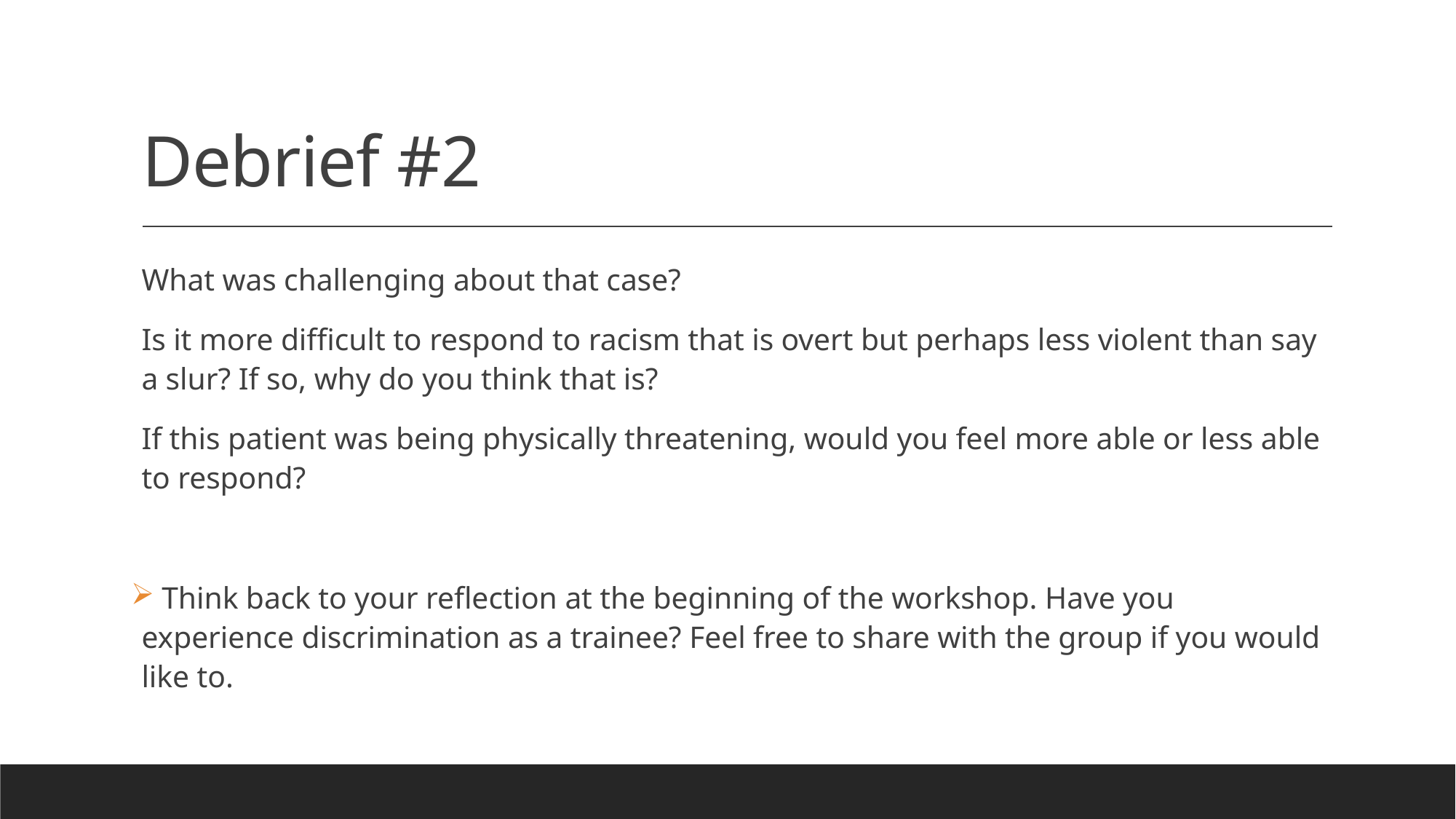

# Debrief #2
What was challenging about that case?
Is it more difficult to respond to racism that is overt but perhaps less violent than say a slur? If so, why do you think that is?
If this patient was being physically threatening, would you feel more able or less able to respond?
 Think back to your reflection at the beginning of the workshop. Have you experience discrimination as a trainee? Feel free to share with the group if you would like to.

## Slide 41
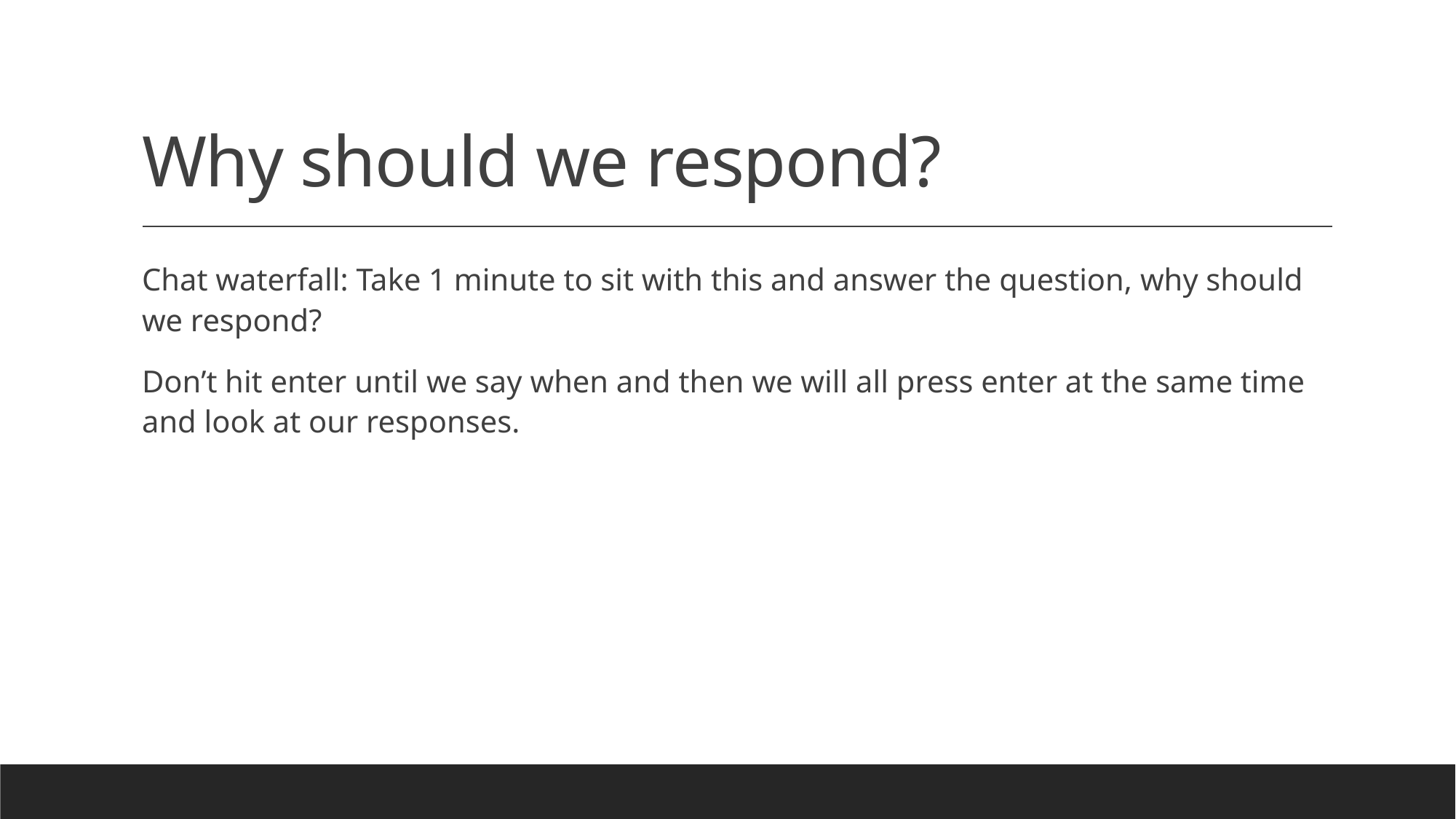

# Why should we respond?
Chat waterfall: Take 1 minute to sit with this and answer the question, why should we respond?
Don’t hit enter until we say when and then we will all press enter at the same time and look at our responses.

## Slide 42
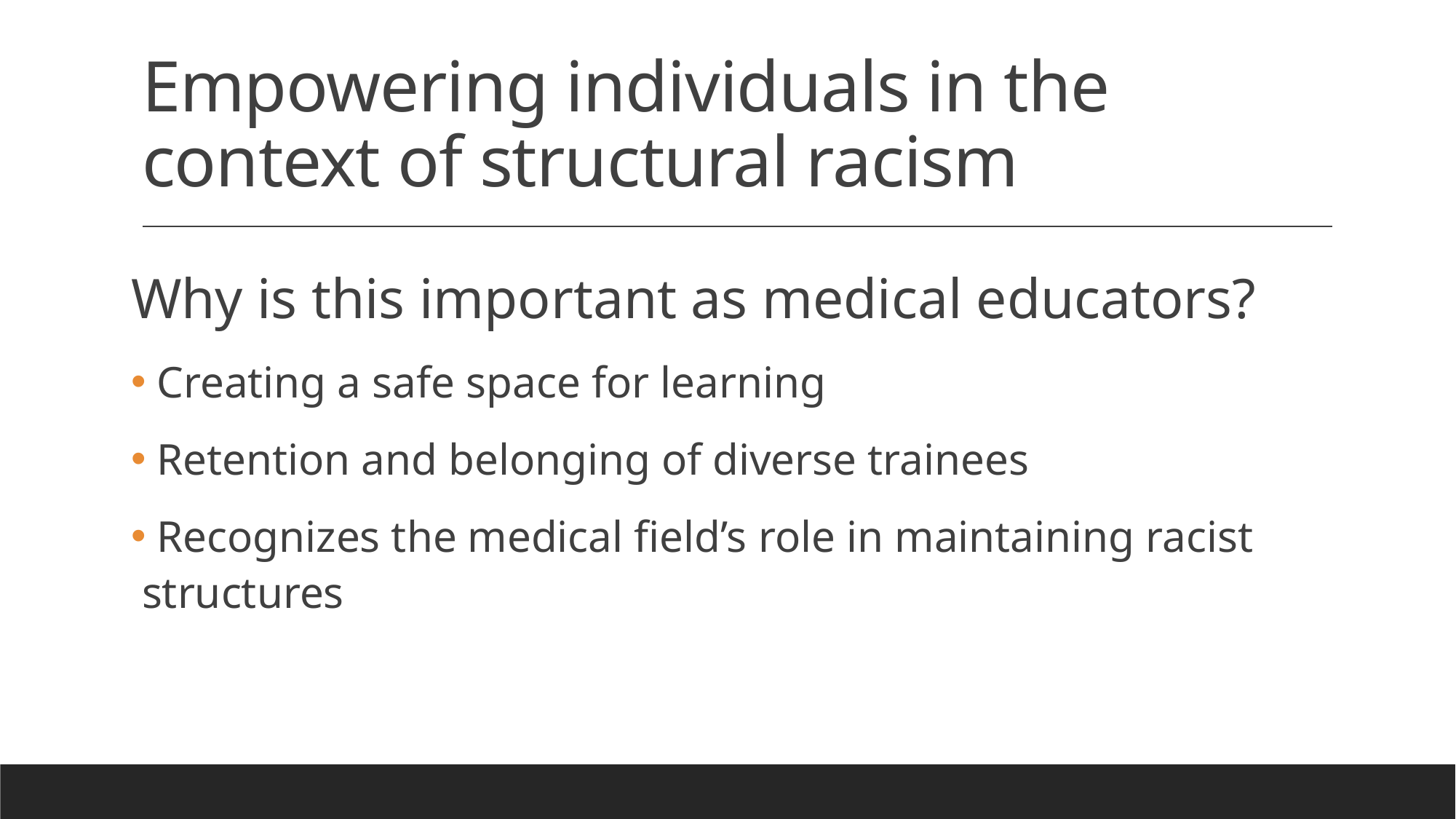

# Empowering individuals in the context of structural racism
Why is this important as medical educators?
 Creating a safe space for learning
 Retention and belonging of diverse trainees
 Recognizes the medical field’s role in maintaining racist structures

## Slide 43
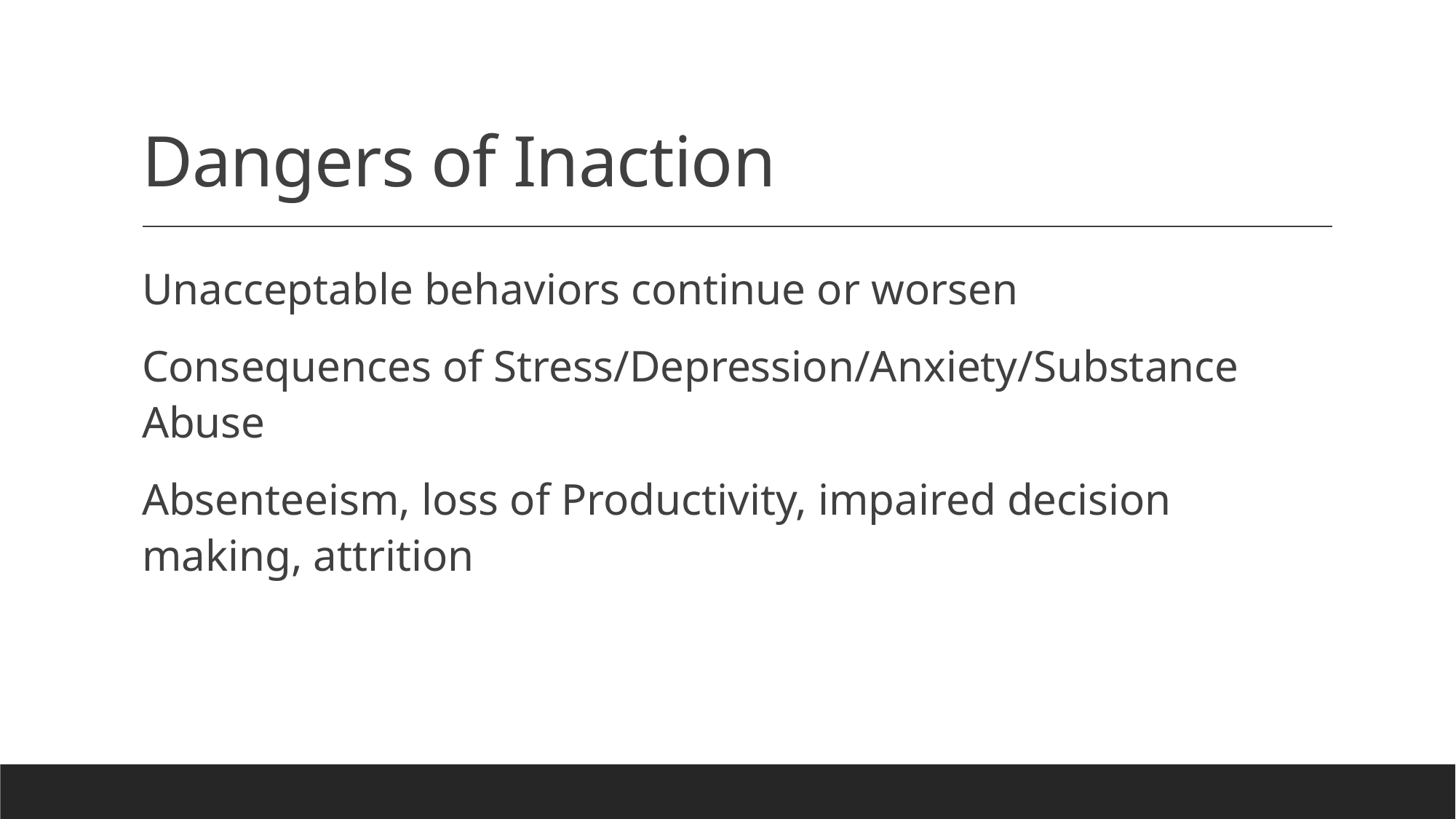

# Dangers of Inaction
Unacceptable behaviors continue or worsen
Consequences of Stress/Depression/Anxiety/Substance Abuse
Absenteeism, loss of Productivity, impaired decision making, attrition

## Slide 44
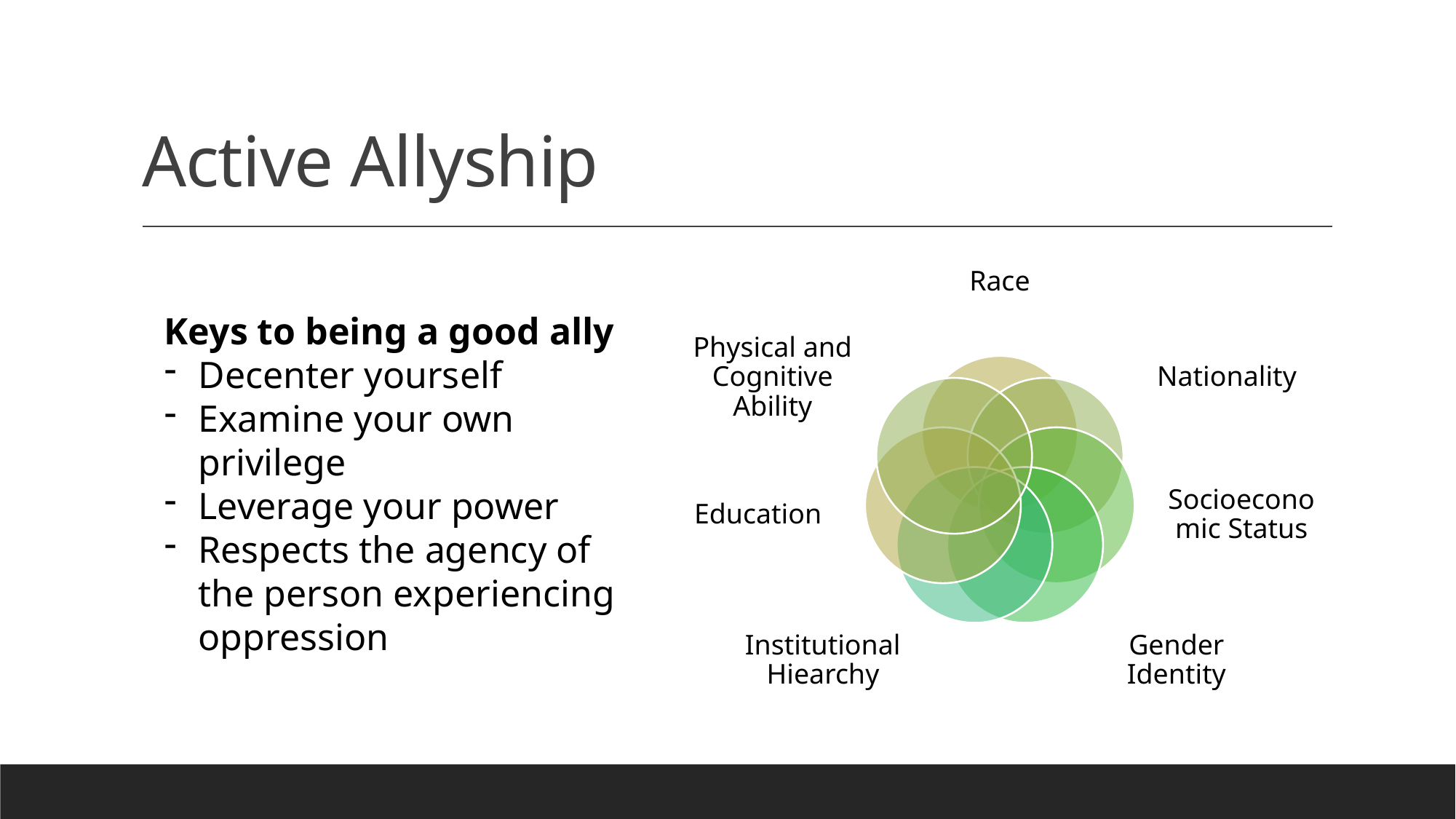

# Active Allyship
Keys to being a good ally
Decenter yourself
Examine your own privilege
Leverage your power
Respects the agency of the person experiencing oppression

## Slide 45
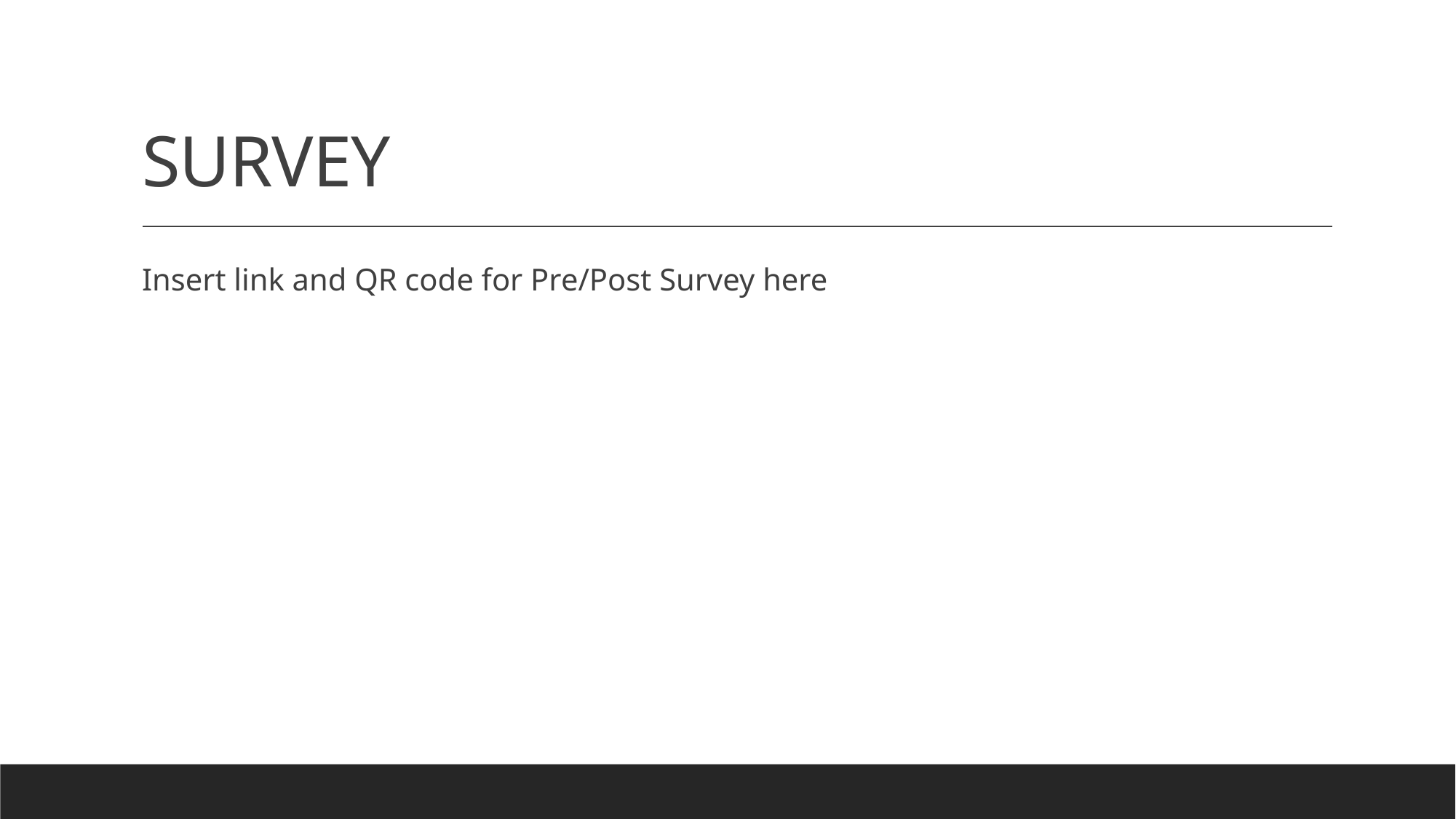

# SURVEY
Insert link and QR code for Pre/Post Survey here

## Slide 46
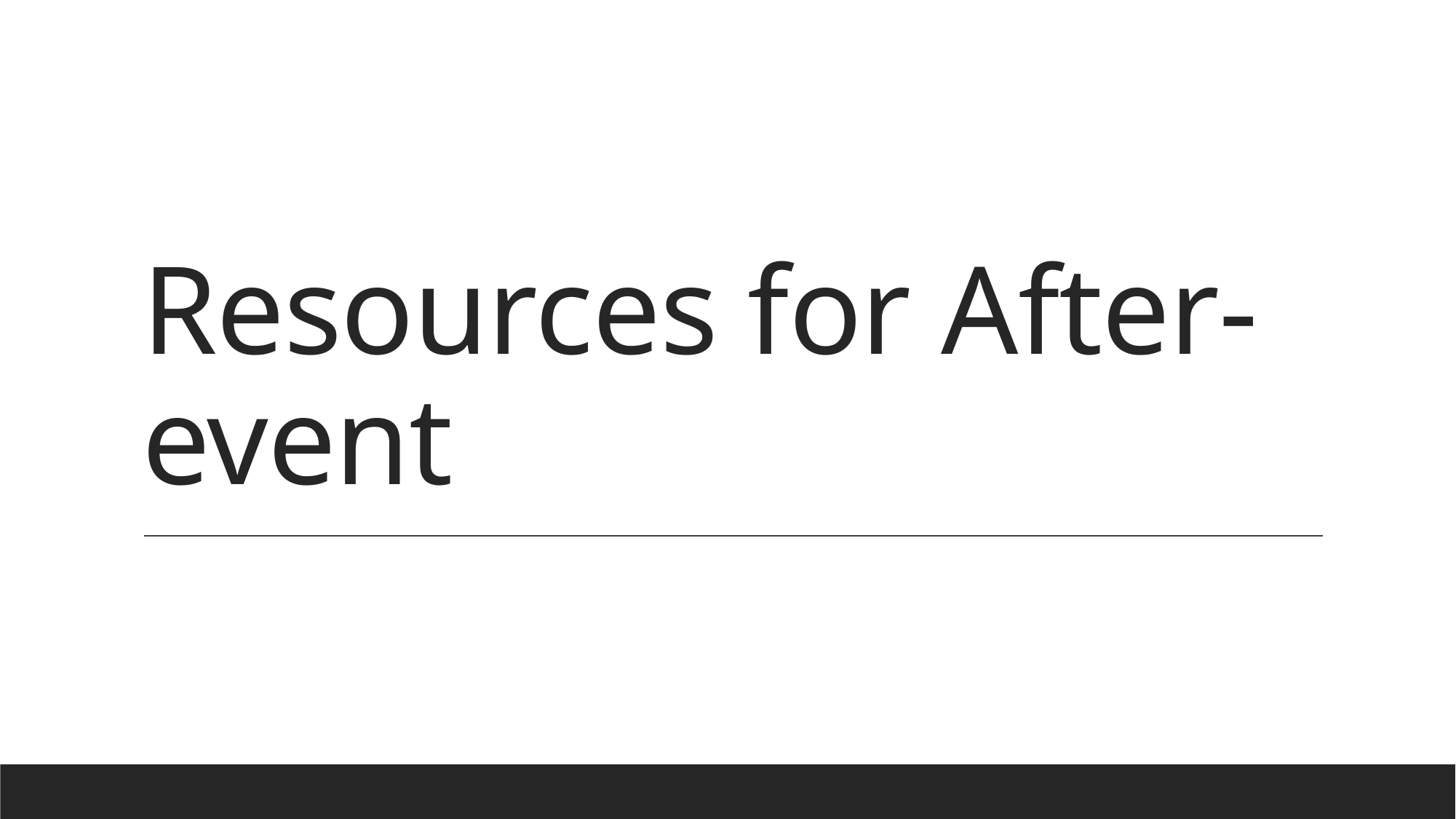

# Resources for After-event

## Slide 47
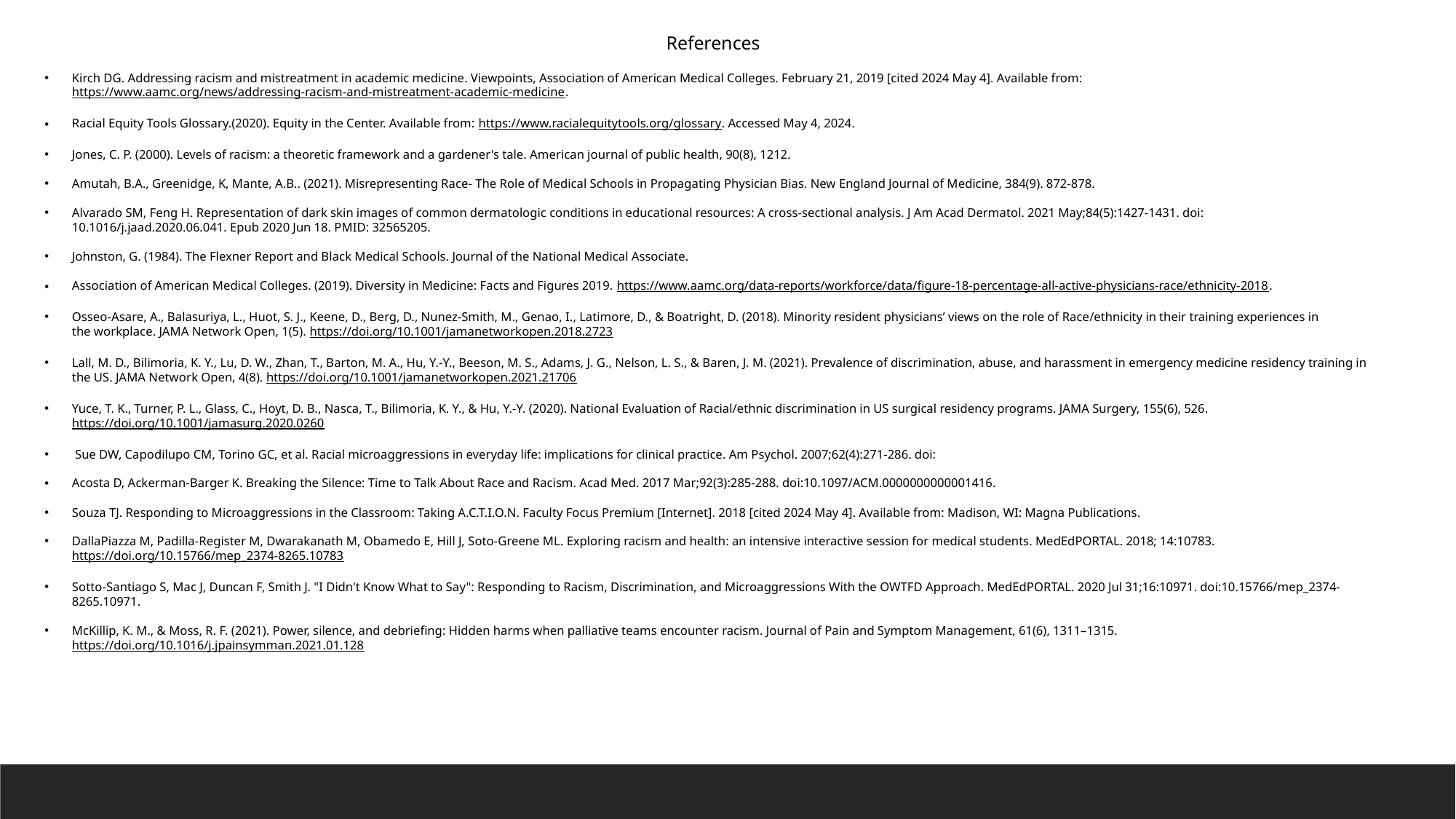

References
Kirch DG. Addressing racism and mistreatment in academic medicine. Viewpoints, Association of American Medical Colleges. February 21, 2019 [cited 2024 May 4]. Available from: https://www.aamc.org/news/addressing-racism-and-mistreatment-academic-medicine.
Racial Equity Tools Glossary.(2020). Equity in the Center. Available from: https://www.racialequitytools.org/glossary. Accessed May 4, 2024.
Jones, C. P. (2000). Levels of racism: a theoretic framework and a gardener's tale. American journal of public health, 90(8), 1212.
Amutah, B.A., Greenidge, K, Mante, A.B.. (2021). Misrepresenting Race- The Role of Medical Schools in Propagating Physician Bias. New England Journal of Medicine, 384(9). 872-878.
Alvarado SM, Feng H. Representation of dark skin images of common dermatologic conditions in educational resources: A cross-sectional analysis. J Am Acad Dermatol. 2021 May;84(5):1427-1431. doi: 10.1016/j.jaad.2020.06.041. Epub 2020 Jun 18. PMID: 32565205.
Johnston, G. (1984). The Flexner Report and Black Medical Schools. Journal of the National Medical Associate.
Association of American Medical Colleges. (2019). Diversity in Medicine: Facts and Figures 2019. https://www.aamc.org/data-reports/workforce/data/figure-18-percentage-all-active-physicians-race/ethnicity-2018.
Osseo-Asare, A., Balasuriya, L., Huot, S. J., Keene, D., Berg, D., Nunez-Smith, M., Genao, I., Latimore, D., & Boatright, D. (2018). Minority resident physicians’ views on the role of Race/ethnicity in their training experiences in the workplace. JAMA Network Open, 1(5). https://doi.org/10.1001/jamanetworkopen.2018.2723
Lall, M. D., Bilimoria, K. Y., Lu, D. W., Zhan, T., Barton, M. A., Hu, Y.-Y., Beeson, M. S., Adams, J. G., Nelson, L. S., & Baren, J. M. (2021). Prevalence of discrimination, abuse, and harassment in emergency medicine residency training in the US. JAMA Network Open, 4(8). https://doi.org/10.1001/jamanetworkopen.2021.21706
Yuce, T. K., Turner, P. L., Glass, C., Hoyt, D. B., Nasca, T., Bilimoria, K. Y., & Hu, Y.-Y. (2020). National Evaluation of Racial/ethnic discrimination in US surgical residency programs. JAMA Surgery, 155(6), 526. https://doi.org/10.1001/jamasurg.2020.0260
 Sue DW, Capodilupo CM, Torino GC, et al. Racial microaggressions in everyday life: implications for clinical practice. Am Psychol. 2007;62(4):271-286. doi:
Acosta D, Ackerman-Barger K. Breaking the Silence: Time to Talk About Race and Racism. Acad Med. 2017 Mar;92(3):285-288. doi:10.1097/ACM.0000000000001416.
Souza TJ. Responding to Microaggressions in the Classroom: Taking A.C.T.I.O.N. Faculty Focus Premium [Internet]. 2018 [cited 2024 May 4]. Available from: Madison, WI: Magna Publications.
DallaPiazza M, Padilla-Register M, Dwarakanath M, Obamedo E, Hill J, Soto-Greene ML. Exploring racism and health: an intensive interactive session for medical students. MedEdPORTAL. 2018; 14:10783. https://doi.org/10.15766/mep_2374-8265.10783
Sotto-Santiago S, Mac J, Duncan F, Smith J. "I Didn't Know What to Say": Responding to Racism, Discrimination, and Microaggressions With the OWTFD Approach. MedEdPORTAL. 2020 Jul 31;16:10971. doi:10.15766/mep_2374-8265.10971.
McKillip, K. M., & Moss, R. F. (2021). Power, silence, and debriefing: Hidden harms when palliative teams encounter racism. Journal of Pain and Symptom Management, 61(6), 1311–1315. https://doi.org/10.1016/j.jpainsymman.2021.01.128
