# Supplementary material for: Recognizing and Responding to Overt Racism Towards Medical Trainees: Using the IRES Tool and Scripted Language
Source: MedEdPORTAL. 2024 Oct 24;20:11453. doi: 10.15766/mep_2374-8265.11453 (PMC11500618; doi:10.15766/mep_2374-8265.11453)
Supplement: Supplementary file 1 — Facilitator Guide.docxSlide Deck.pptxPractice Cases.docxIRES Tool.docxScripted Language.docxPostworkshop Evaluation.docx [file mep_2374-8265.11453-s001.zip › F. Postworkshop Evaluation.docx]

Appendix F. Postworkshop Evaluation

Recognizing and Responding to Overt Racism Postsession Survey

Thank you for participating in the Recognizing and Responding to Overt Racism workshop. Please complete the following survey. Our intention is to improve faculty’s confidence in their ability to respond in the moment to racism in the learning environment. Cumulative, deidentified information from the survey will be used to improve the workshop and may be published as part of medical education research. Responses will be confidential. Consent to participate will be implied by completion of this survey. Participation is voluntary, if you do not wish to participate simply close out the survey. Non-participation in the survey will not affect your participation in the workshop. Thank you !

Demographic Information

1. Department
2. Position/ Title
3. Age
   1. 18-24
   2. 25-34
   3. 35-44
   4. 45-54
   5. 55-64
   6. 65 and over
4. Gender
5. Race

For the following questions please rank your perception BEFORE the workshop and AFTER the workshop

1. Learners from diverse backgrounds face microaggressions in clinical rotations.
   1. Perception BEFORE workshop
      - Strongly Disagree
      - Disagree
      - Neutral
      - Agree
      - Strongly Agree
   2. Perception AFTER workshop
      - Strongly Disagree
      - Disagree
      - Neutral
      - Agree
      - Strongly Agree
2. Learners from diverse backgrounds face racism in clinical rotations.
   1. Perception BEFORE workshop
      - Strongly Disagree
      - Disagree
      - Neutral
      - Agree
      - Strongly Agree
   2. Perception AFTER workshop
      - Strongly Disagree
      - Disagree
      - Neutral
      - Agree
      - Strongly Agree
3. Exposure to chronic microaggressions in the learning environment results in diminished academic performance, social withdrawal, anxiety and depression.
   1. Perception BEFORE workshop
      - Strongly Disagree
      - Disagree
      - Neutral
      - Agree
      - Strongly Agree
   2. Perception AFTER workshop
      - Strongly Disagree
      - Disagree
      - Neutral
      - Agree
      - Strongly Agree
4. I can name specific historical incidents and systemic ways in which the medical system has upheld structural inequity and racism.
   1. Perception BEFORE workshop
      - Strongly Disagree
      - Disagree
      - Neutral
      - Agree
      - Strongly Agree
   2. Perception AFTER workshop
      - Strongly Disagree
      - Disagree
      - Neutral
      - Agree
      - Strongly Agree
5. The medical community has a responsibility to acknowledge systemic inequities and work to correct them on an individual, institutional and societal level.
   1. Perception BEFORE workshop
      - Strongly Disagree
      - Disagree
      - Neutral
      - Agree
      - Strongly Agree
   2. Perception AFTER workshop
      - Strongly Disagree
      - Disagree
      - Neutral
      - Agree
      - Strongly Agree
6. The language and strategies for addressing microaggressions are different than for addressing egregious racism.
   1. Perception BEFORE workshop
      - Strongly Disagree
      - Disagree
      - Neutral
      - Agree
      - Strongly Agree
   2. Perception AFTER workshop
      - Strongly Disagree
      - Disagree
      - Neutral
      - Agree
      - Strongly Agree
7. I feel confident in my ability to address a microaggression when it occurs
   1. Perception BEFORE workshop
      - Strongly Disagree
      - Disagree
      - Neutral
      - Agree
      - Strongly Agree
   2. Perception AFTER workshop
      - Strongly Disagree
      - Disagree
      - Neutral
      - Agree
      - Strongly Agree
8. I feel confident in my ability to distinguish a microaggression from overt racism.
   1. Perception BEFORE workshop
      - Strongly Disagree
      - Disagree
      - Neutral
      - Agree
      - Strongly Agree
   2. Perception AFTER workshop
      - Strongly Disagree
      - Disagree
      - Neutral
      - Agree
      - Strongly Agree
9. I feel confident in my ability to know how to respond to microaggressions versus overt racism.
   1. Perception BEFORE workshop
      - Strongly Disagree
      - Disagree
      - Neutral
      - Agree
      - Strongly Agree
   2. Perception AFTER workshop
      - Strongly Disagree
      - Disagree
      - Neutral
      - Agree
      - Strongly Agree
10. I feel confident in my ability to debrief a learner who has been the target of of a microaggression or overt racism.
    1. Perception BEFORE workshop
       - Strongly Disagree
       - Disagree
       - Neutral
       - Agree
       - Strongly Agree
    2. Perception AFTER workshop
       - Strongly Disagree
       - Disagree
       - Neutral
       - Agree
       - Strongly Agree
11. Describe one takeaway from today’s workshop
12. What was effective about this workshop?
13. What needs improvement ?
