# Supplementary material for: Recognizing and Responding to Overt Racism Towards Medical Trainees: Using the IRES Tool and Scripted Language
Source: MedEdPORTAL. 2024 Oct 24;20:11453. doi: 10.15766/mep_2374-8265.11453 (PMC11500618; doi:10.15766/mep_2374-8265.11453)
Supplement: Supplementary file 1 — Facilitator Guide.docxSlide Deck.pptxPractice Cases.docxIRES Tool.docxScripted Language.docxPostworkshop Evaluation.docx [file mep_2374-8265.11453-s001.zip › _Educational Summary Report_11453.pdf]

MedEdPORTAL® The AAMC Journal of Teaching and Learning Resources

Original Publication Open Access

Recognizing and Responding to Overt Racism Towards Medical Trainees: Using the IRES Tool and Scripted Language

Shauna M. Gibbons, MD*, Kelli E. Krase, MD, Lindy H. Landzaat, DO, Lori A. Spoozak, MD, MHS

*Corresponding author: sgibbons@kumc.edu

Abstract

Introduction: Medical educators feel ill equipped to respond to racism directed towards trainees. Available tools for responding to microaggressions rely on clarifying questions or aligning with the aggressor, which may add to the harm experienced by the target. We aimed to provide methods for faculty to recognize and respond to overt racism so that trainees feel supported in the clinical learning environment. **Methods:** We created a faculty development workshop with didactic and experiential learning components based on Kolb's theory for program leadership to teach how to respond when racism was directed towards trainees. In the didactic session, we shared the IRES (identify, respond, end, support) tool we created to respond to overt racism. Participants took part in two small-group case-based practice sessions with group debriefing, followed by a postsession survey. **Results:** Over 2 years, we held two sessions with a total of 43 faculty participants. Postsession survey response rate was 74% and showed a 1-point Likert-scale median increase ($p < .001$) in ability to distinguish the strategies for addressing overt racism versus microaggressions, confidence in responding to microaggressions versus overt racism, and ability to debrief learners. The majority of participants noted that scripted language was a valuable tool to promote upstander behavior. **Discussion:** Participants appreciated this novel framework for responding to racism directed at trainees in the learning environment. Providing scripted language and being able to practice in a safe environment were particularly important. This training can be adapted to include residents, fellows, and other disciplines.

Keywords
Discrimination, Microaggression, Racism, Scripting, Anti-racism, Case-Based Learning, Faculty Development, Diversity, Equity, Inclusion

Educational Objectives

By the end of this workshop, faculty will be able to:

1. Differentiate between the concepts of bias, microaggressions, and overt racism.
2. Contrast the response to microaggressions versus overt racism.
3. Understand the role of academic faculty as upstanders and allies to trainees from diverse backgrounds.
4. Apply the IRES (identify, respond, end, support) tool and scripted language to identify and interrupt racism directed toward a trainee.

Citation:
Gibbons SM, Krase KE, Landzaat LH, Spoozak LA. Recognizing and responding to overt racism towards medical trainees: using the IRES tool and scripted language. *MedEdPORTAL*. 2024;20:11453.
https://doi.org/10.15766/mep_2374-8265.11453

Introduction

The Liaison Committee on Medical Education and the Accreditation Council for Graduate Medical Education have made recruitment and retention of diverse medical students, residents, and fellows a priority and thus a requirement for accreditation. Despite this, many institutions struggle to create a safe learning environment for the diverse learners that they recruit.[1,2] Experiencing racism and other forms of discrimination, especially without consistent support, causes social isolation, depression, and attrition amongst trainees from diverse backgrounds.[3-6] This became a particularly urgent issue in 2020. The death of George Floyd, the inequities exposed by the COVID-19 pandemic, and the tense political climate caused ripple effects felt at both national and local levels. Our institution saw increasing incidents of overt racism and violent language being directed towards learners by patients and family members, and faculty felt ill equipped to deal with these episodes in the moment. There were no local, institutional, or GME-level antidiscrimination statements at the time, and the only system-level coaching available was unconscious bias training, leaving faculty feeling

Copyright © 2024 Gibbons et al. This is an open-access publication distributed under the terms of the Creative Commons Attribution license.

unprepared and unsupported to combat racism directed at learners.

To address these gaps, in 2020 we created a faculty development workshop that focused on identification, interruption, and response to overt racism directed towards learners. We were motivated to respond locally to a national call to action urging medical training programs to address racism and discrimination on both an institutional and an individual level.[7-9] Unfortunately, many medical educators do not feel equipped to respond to these incidents when they occur. Reasons for this are multifactorial, including fear of alienating patients, not having institutional backing, fear of escalation, freezing, or not knowing what to say.[10] Faculty may respond with tools that are more appropriate for microaggressions or that may add to the harm by using communication skills relying too heavily on alignment or negotiation with the aggressor.[11]

Several useful approaches to address both systemic and interpersonal racism in medicine have been published. The DARE training offers a way for faculty to identify and address racism in preclinical or didactic medical curricula.[12] Interpersonal racism, including bias, microaggressions, and overt racism, remains a significant problem for medical trainees, and chronic exposure has been shown to have many detrimental effects on well-being and performance.[4,13] In the clinical setting, patients and their families are the most frequent sources.[14] Publications on techniques such as OWTFD, ACTION, OTFD, and INTERRUPT share useful approaches to addressing microaggressions that incorporate asking clarifying questions and leaving space for mutual understanding.[8,15-19] However, responding to overt or violent racist language the same way one would respond to a microaggression runs the risk of minimizing the offense and can add harm to the person who is targeted.[11] For instance, if a trainee is called a racial slur, it would not be appropriate to ask what the perpetrator meant or to engage in a conversation about what it made the target think and feel. This approach also does not emphasize the role of faculty as an upstander. By nature of the power dynamic at play, faculty are in a unique position to intervene. Other training courses do not address the important role of providing support to the target, particularly when that individual is a trainee.

Our faculty development workshop builds on several previously published works to address the gap in training available to faculty. Like Neves da Silva and colleagues' training for medical students, we use role-play and debriefing as an educational strategy.[20] We add to the literature and innovate in our focus on faculty role, our emphasis on the need for a unique response to overt racism, and the use of scripted language. Furthermore, where Neves da Silva and colleagues go into detail about analyzing and addressing several manifestations of racism ranging from subtle to explicit, our workshop has a narrow focus on overt racism. We chose this narrow focus based on cognitive load theory used in medical simulation curricula.[21] We created the IRES (identify, respond, end, support) tool and accompanying scripted language to facilitate a direct, in-the-moment response to instances of racism. Based on the theory of transformative learning, we ask participants to consider the similarities and differences between microaggressions and overt racism, as well as how their responses to such incidents could impact the effect on the learner in either a positive or a negative way. Using the Kolb cycle of experiential learning, we ask participants to practice and experiment with scripted responses and reflect on the experience in debrief sessions.[22] The workshop emphasizes how to respond firmly, end the encounter, and debrief the learner. Our approach is intentionally simple and direct as seen in stop, talk, and roll (STR). We innovate by building on STR and RISE UP through incorporating the use of scripted language and providing a case-based role-play that is specific to faculty members and emphasizes the role of support and debriefing the leaner after an event.[23,24] Scripted language, communication talking maps, and role-play are often used in medical simulation curricula and in training programs such as VitalTalk to teach clinicians communication methods for navigating serious illness conversations that are potentially emotionally volatile.[25] With evidence supporting its use in these situations, we believe scripted language can be a useful approach for learning how to respond to incidents of racism and discrimination in clinical encounters.[26]

Methods

Using Kern's method of curriculum development,[27] we created a workshop at our institution to teach faculty how to identify overt racism directed at medical trainees in the clinical environment and respond in a way that would minimize harm to the trainee as outlined below.

Needs Assessment

Because of our roles as program directors of various fellowship and residency training programs, faculty members brought to our attention several incidents of racism and discrimination directed towards learners during which they felt that they did not know how to respond. We reviewed institutional policies and training, as well as the literature, and found that there were no tools or institutional policies to provide faculty with guidance on how to respond during these situations.

Copyright © 2024 Gibbons et al. This is an open-access publication distributed under the terms of the Creative Commons Attribution license.

Targeted Needs Assessment

We conducted an informal survey of the OB/GYN and palliative medicine physician groups. The consensus opinion was that our institution's implicit bias training, the only faculty training resource at the time of the workshop's development, did not address overt racism. It did not focus on action or language and left faculty feeling unprepared for racist encounters. Additionally, a literature review at the time revealed no resources published that specifically addressed racism rather than microaggression response. To address this gap, using the nominal group technique we created the IRES tool and scripted language to be shared in a faculty development workshop focused on experiential learning to facilitate behavioral change. A pilot session was conducted with 15 faculty and fellows in the palliative medicine division and was 1 hour long. This was followed by a session with the OB/GYN department that involved 25 faculty and was 2 hours long. Both sessions included an introductory didactic component and a case-based practice component followed by a postsession survey to evaluate and improve the workshop. Participants valued having a framework for identifying racism versus microaggressions, having scripted language, and having time to practice it. The focus action was appreciated and not available in other courses. The didactic component was found to be too long. With the feedback gained from the pilot sessions, our group partnered with our institution's GME leadership to offer this faculty development workshop to all GME training programs.

After our initial needs assessment, we determined that the workshop should first be provided to faculty in positions of GME leadership, specifically, program directors, associate and assistant program directors, and core faculty, with the hope that they would be able to disseminate key learning points to others in their respective programs. In this iteration, we decided to provide the workshop to faculty and not trainees since we wanted to emphasize the power dynamic that should facilitate faculty to act more readily in these situations. We did acknowledge that the problem of racism being directed towards the attending physician, although not addressed in the workshop, was deeply harmful, and we allowed space for faculty to share their stories if desired.

For the GME sessions, we added an icebreaker session to the introduction, which had not been present in the pilot, to help facilitate open communication about this difficult subject between a group of individuals who were relative strangers. We believed that having an icebreaker session was a valuable tool in opening the group to conversation, though it did consume more time than expected. For this reason and since participants desired more time for role-playing and debriefing, the time allotted was reformatted to give the most attention to cases and discussion.

Our workshop focused on the aggressor being a patient or family member with capacity in a nonemergent situation. Several participants noted that delivering emergent medical care to an unstable patient would hinder one's ability to exit the encounter or that the communication tools used would not have the same effect if the aggressor's mental status were altered. We agreed with this and added specific language in the introduction to frame the appropriate clinical situation in which to use this tool.

Goals and Objectives

The goal of this workshop was to enable medical faculty to recognize and respond to overt racism targeting medical trainees. By the end of the workshop, faculty would be able to (1) differentiate between the concepts of bias, microaggressions, and overt racism; (2) contrast the response to microaggressions versus the response to overt racism; (3) understand the role of academic faculty as an upstander and ally to trainees from diverse backgrounds; and (4) apply the IRES tool and scripted language to interrupt racism targeting trainees in a clinical encounter.

The facilitators held two sessions over a 2-year period with 43 participants in total, 20 for the first session and 23 for the second. The workshop was open to program directors, assistant program directors, and core faculty across our institution's GME training programs. Each session was facilitated by the four faculty members who had authored the session and followed the same design outlined in the facilitator guide (Appendix A). The workshop began with a didactic portion, with slides that provided a review of the literature on harm caused by exposure to both microaggressions and overt racism in medicine, the importance of upstander behavior, and tools for responding to microaggressions versus responding to overt racism (Educational Objectives 1 and 2; Appendix B). This was followed by the experiential learning component with two case-based scenarios (Appendix C) to practice application of the IRES tool (Appendix D) and scripted language (Appendix E) with facilitated debriefing (Educational Objective 3).

Educational Strategies

Didactic and experiential learning: The workshop was grounded in transformative learning by offering an opportunity to practice applying knowledge provided in the didactic session to cases followed by debriefing and reflection. We aimed to provide an experience that expanded participants' existing skills to enter a zone of growth.[28] It was imperative that we establish a

Copyright © 2024 Gibbons et al. This is an open-access publication distributed under the terms of the Creative Commons Attribution license.

psychologically safe learning environment at the beginning of the session given the subject's difficult nature. We encouraged participants to approach each person's input with a sense of curiosity and acknowledged that it was okay to opt out if needed as this topic could be very difficult for some. Being aware of the differential needs of staff belonging to underrepresented groups during the session was of utmost importance to avoid retraumatization. We intentionally structured role-play cases without offensive language or asking anyone to act as the aggressor. We established that the session would not be recorded, participation would not be evaluated, and nothing shared by the audience during our discussion would leave the session. It was important to communicate that each of us, facilitators included, were all still learning and did not assume expertise.

Due to the COVID-19 pandemic, the workshop was held virtually via Zoom. To foster a sense of safety and trust, we asked each participant to have their camera turned on. We also had a short icebreaker exercise since participants came from programs across GME and might not have met each other before. The four authors facilitated the didactic portion by each presenting a section of the slides (Appendix B.) We began with a brief discussion of the historical and structural context of racism in medicine (slides 12-17) to give better context to the experience of diverse trainees entering this environment. The remaining didactic portion was spent teaching faculty how to identify and differentiate implicit bias, microaggressions, and overt racism. We then shared the IRES tool (Appendix D) and scripted language handouts (Appendix E).

Intervention tools and scripted language: Examples of published microaggression intervention tools were shown during the presentation (slide 28). Participants were given the IRES tool (Appendix D), and scripted language (Appendix E) developed by our group to be used in response to overt racism. Our experience and education in communication through practice of palliative medicine in an academic setting were useful in crafting the scripted language, which was refined using the nominal group technique. Additionally, through experience leading communication-based medical simulation and after a review of the literature, we determined that providing a script, instead of asking faculty to come up with their own responses, was an important component of overcoming the fight, flight, or freeze response to an emotionally intense encounter.[26]

Case scenarios and small-group experiential learning: We created two role-play case scenarios, deliberately excluding offensive language and not having anyone play the aggressor role to avoid retraumatization or singling anyone out. We divided the group into pairs using Zoom breakout rooms after provided them with the cases (Appendix C), the IRES tool (Appendix D), and the scripted language (Appendix E). Participants were asked not to act out the role of the aggressor but instead to role-play as the upstander by reading the case and to simulate responding aloud to their partner using the scripted language (Appendix E). The pairs reflected on how it felt to respond and which phrases felt more natural to them and gave feedback to each other. Participants also practiced supporting the trainee targeted in the scenario with the aid of suggested language (Appendix E).

Facilitated large-group debriefing and reflective learning: After each practice session, the facilitators conducted a large-group debrief. We began this session by reiterating that it was a safe container for discussion and providing permission for participants to opt out at any time. Participants reflected on how it felt to respond to each case, what impact the IRES tool and scripted language had on their ability to respond, and the importance of having an appropriate response to these incidents. They were also invited to share their own experiences as a bystander, upstander, or target if they felt comfortable doing so. After reflection, we provided a list of resources available at our institution to support learners and faculty.

Evaluation
At the conclusion of the workshop, we distributed a survey to all participants (Appendix F). The University of Kansas Institutional Review Board had determined it to be exempt, and participants were told that completion of the survey was voluntary. Survey responses were submitted through and stored in our institution's online survey database (REDCap). Participants rated their understanding of the concepts taught, confidence in ability to respond to overt racism, and confidence supporting the trainee using a 5-point Likert scale (anchors: *strongly disagree, strongly agree*). They rated perceived pre- and postworkshop scores for all domains. Finally, they completed three open-ended questions to understand what concepts resonated with them and what could be improved.

Data Analysis
We used GraphPad Prism Version 9.0.0 to perform statistical analysis. The Wilcoxon matched pairs signed rank test compared the difference in change between participants' perceived pre- and postworkshop scores. We conducted a qualitative review of participants' responses to open-ended questions to identify common themes.

Copyright © 2024 Gibbons et al. This is an open-access publication distributed under the terms of the Creative Commons Attribution license.

Results

A total of 43 participants completed the GME workshop, and 32 participants completed the survey, resulting in a response rate of 74%. We asked participants to provide self-reported demographic information. A wide range of departments were represented, and most individuals were currently serving as program directors or assistant program directors. Seventy-eight percent of participants self-identified as White, with the next most common demographic group being Asian/Asian American/South Asian at 9%. Full demographic responses are presented in Table 1. The postsession survey showed a 1-point Likert-scale median increase ($p < .001$) in participants' ability to distinguish the language and strategies for addressing overt racism versus microaggressions, to respond to microaggressions versus overt racism in a clinical encounter, and to debrief learners who were targets of overt racism. The session resulted in a shift in percentage of the group who agreed or strongly agreed that they were equipped to respond to racism in clinical encounters (Table 2). Four main themes were identified through qualitative analysis of the open-ended feedback: (1) the impact of role-play, (2) the value of scripted language, (3) the focus on action-oriented behavior, and (4) the importance of creating a psychologically safe environment (Table 3).

Table 1. Demographics ($N = 32$)

Demographic	No. (%) of Participants
Self-reported race/ethnicity	
Asian/Asian American/South Asian	3 (9)
Black/African American/African	0 (0)
Hispanic	2 (6)
Middle Eastern	1 (3)
Mixed race	1 (3)
White	25 (78)
Gender	
Female	19 (59)
Male	13 (41)
Age range	
25-34	2 (6)
35-44	14 (44)
45-54	14 (44)
55-64	1 (3)
65+	1 (3)
Department	
Anesthesiology	2 (6)
Emergency medicine	3 (9)
Family medicine	3 (9)
Internal medicine and subspecialties	8 (25)
Neurology	1 (3)
Obstetrics and gynecology	3 (9)
Pathology	1 (3)
Psychiatry	2 (6)
Radiation oncology	1 (3)
Surgical specialties (ophthalmology, urology, neurosurgery, plastic surgery, otolaryngology)	8 (25)
Role in GME	
Program director	10 (31)
Assistant/associate program director	4 (12)
Other faculty	18 (56)

Discussion

The focus of this faculty development workshop was on providing medical educators with a tool to help recognize racism and utilizing scripted language to interrupt racism targeting trainees. Previously published workshops have focused on education about microaggressions and racism but have not provided preparedness tools for upstander intervention.[29-31] Here, we add to the literature with a workshop that significantly improved faculty knowledge and confidence.

This workshop is novel in its use of scripted language, role-play, and reflection to teach educators how to respond to racial discrimination in the clinical learning environment, addressing an unmet need at our institution and more broadly in the medical education literature. DallaPiazza and colleagues, Acosta and Ackerman-Barger, Sotto-Santiago and colleagues, and Souza offer several useful tools for responding to microaggressions, and we add to the literature by differentiating the response to overt racism, providing scripted language, and avoiding the pitfall of using clarifying or aligning language for overt racism.[1,8,15,19,29] We build on the simple, direct structure of STR and RISE UP by constructing a workshop with simulation, using scripted language, and focusing on the additional role of faculty as support for trainees.[23,24] By offering faculty-specific training, our workshop also adds to the work done by Neves da Silva and colleagues that offers similar training for students. The results show that participants valued being given the IRES tool and scripted language and that the experiential portion of the workshop was integral to improving confidence to make a behavioral change. Based on feedback desiring more time for role-play and debriefing, we found that a longer session of 1.5-2 hours was preferable to 1 hour. Results also showed that contrasting the communication tools proposed for addressing microaggressions to those we proposed for instances of overt racism was valuable.

Since the time of this workshop's development, many actions have come out of our institutional reckoning with racism on campus, such as a GME-specific and then subsequently hospital system–wide antidiscrimination policy addressing racism and protections of faculty, trainees, and staff. We additionally have antidiscrimination training embedded in our annual compliance training for the health system. We believe that our faculty development workshop is an important contribution to drive change at our predominantly White institution and that other

Copyright © 2024 Gibbons et al. This is an open-access publication distributed under the terms of the Creative Commons Attribution license.

Table 2. Percentage of Participant Confidence in Ability to Respond to Racism in Clinical Encounters ($N = 32$)

Question	Strongly Disagree	Disagree	Neutral	Agree	Strongly Agree
I feel confident in my ability to debrief a learner who has been the target of a microaggression or overt racism.					
Presession	0%	25%	22%	50%	3%
Postsession	0%	0%	0%	50%	50%
I feel confident in my ability to know how to respond to microaggressions versus overt racism.					
Presession	0%	15%	38%	44%	3%
Postsession	0%	0%	8%	64%	28%
I feel confident in my ability to identify a microaggression when it occurs.					
Presession	0%	6%	25%	68%	0%
Postsession	0%	0%	3%	75%	22%
The language and strategies for addressing microaggressions are different than addressing egregious racism.					
Presession	0%	6%	28%	56%	9%
Postsession	0%	6%	3%	38%	53%

programs may find it similarly useful to help promote a culture of anti-racism. More work needs to be done on institutional and systemic levels to find solutions and help protect all health care workers.

There are several important limitations to this workshop. We brought to this project expertise in communication and medical education, prior training in bias and microaggressions in medicine, and personal experience of navigating medical training as members of marginalized groups, but importantly, we are not experts in this field. We suggest faculty facilitating this workshop should have baseline familiarity with anti-racism and microaggressions. While we hope the simplicity of our method can result in behavioral change, it is important to acknowledge that our one-session workshop by no means results in expertise, and we have not demonstrated the tool's effectiveness in a clinical setting. We also acknowledge that our institutional leadership is not racially diverse and that, by extension, we were not able to get the perspective of Black physicians participating in this workshop, which is a significant limitation. As shared by several non-White faculty members, racism towards faculty is also a significant problem that this workshop does not address. Furthermore, these faculty members must contend with more complex and difficult power dynamics when choosing to respond to racism directed at a trainee.

Overall, the workshop was well received by participants and GME leadership, and there was a desire for more sessions to

Table 3. Themes From Open-Ended Feedback

Theme	Theme Explanation	Representative Quote
The impact of role-play	Most participants felt that practicing responding was a key part of the workshop and important to improving their confidence in their ability to respond. Simply saying these phrases out loud to their partner, having the opportunity to try on different language, and receiving feedback from their partner were the top-cited reasons why the role-play was valuable. Several participants desired more time for dedicated practice, and no participants requested less time.	"Making us role play was very important. . . . Also appreciate this was across specialties, this helps get the whole GME organization on the same page."
The value of scripted language	When presented with violent or racist behavior, participants report feeling frozen and at a loss for words. Having prepared language during these stressful situations lessens the cognitive burden in the moment and provides space to act.	"I have scripting I didn't have before and was able to practice the words, which I think will empower me to be able to not be a bystander. I'm already thinking back on scenarios I've been a part of and how I would handle it now with this scripting."
The focus on action-oriented behavior	Participants noted that training they had received in the past often centered around creating awareness but did not give participants tools to translate their desire to be an upstander into the real world. In addition to providing these tools, we emphasized that responding in these scenarios is not a matter of going above and beyond, it is simply the right thing to do.	"Realizing I should react to verbal racism the same way as physical violence and not hesitating to protect the learner."
The importance of creating a psychologically safe environment	Taking care to ensure a safe learning environment is essential for conducting this workshop. The time spent at the beginning of the session establishing clear ground rules and a shared understanding of purpose was cited as a key part of this experience.	"The interactions and practice. Very safe space"

Copyright © 2024 Gibbons et al. This is an open-access publication distributed under the terms of the Creative Commons Attribution license.

be provided. We look forward to offering the workshop yearly to GME leadership and hope to expand it to all faculty working with trainees. Because of its simplicity, the workshop lends itself to being offered to a wider audience of faculty working with trainees, not just those in leadership roles. With some adaptation of the literature review, it could also be offered to residents and fellows. Developing an accompanying train-the-trainer course would be an important next step to ensure consistency as the program grows. We look forward to expanding this workshop to reach a larger audience of medical educators creating safer places for diverse learners to thrive.

Appendices

A. Facilitator Guide.docx
B. Slide Deck.pptx
C. Practice Cases.docx
D. IRES Tool.docx
E. Scripted Language.docx
F. Postworkshop Evaluation.docx

All appendices are peer reviewed as integral parts of the Original Publication.

Shauna M. Gibbons, MD: Assistant Professor, Division of Palliative Medicine, Department of Internal Medicine, University of Kansas School of Medicine; ORCID: https://orcid.org/0009-0008-1571-9364

Kelli E. Krase, MD: Assistant Professor, Department of Obstetrics and Gynecology, University of Kansas School of Medicine

Lindy H. Landzaat, DO: Associate Professor, Division of Palliative Medicine, Department of Internal Medicine, University of Kansas School of Medicine

Lori A. Spoozak, MD, MHS: Associate Professor, Divisions of Gynecology Oncology and Palliative Medicine, Department of Obstetrics and Gynecology, University of Kansas School of Medicine

Disclosures
None to report.

Funding/Support
None to report.

Prior Presentations
Gibbons S. Responding to racism in the learning environment. Poster presented at: ACGME Annual Education Conference; February 23-25, 2023; Nashville, TN.

Ethical Approval
The University of Kansas Medical Center Institutional Review Board deemed further review of this project not necessary.

References

1. Acosta D, Ackerman-Barger K. Breaking the silence: time to talk about race and racism. *Acad Med*. 2017;92(3):285-288. https://doi.org/10.1097/ACM.0000000000001416
2. Argueza BR, Saenz SR, McBride D. From diversity and inclusion to antiracism in medical training institutions. *Acad Med*. 2021;96(6):798-801. https://doi.org/10.1097/ACM.0000000000004017
3. Blackstock U. Why Black doctors like me are leaving faculty positions in academic medical centers. STAT. January 16, 2020. Accessed September 23, 2024. https://www.statnews.com/2020/01/16/black-doctors-leaving-faculty-positions-academic-medical-centers/
4. Osseo-Asare A, Balasuriya L, Huot SJ, et al. Minority resident physicians' views on the role of race/ethnicity in their training experiences in the workplace. *JAMA Netw Open*. 2018;1(5):e182723. https://doi.org/10.1001/jamanetworkopen.2018.2723
5. Kirch DG. Addressing racism and mistreatment in academic medicine. Association of American Medical Colleges. February 21, 2019. Accessed September 23, 2024. https://www.aamc.org/news/addressing-racism-and-mistreatment-academic-medicine
6. Torres MB, Salles A, Cochran A. Recognizing and reacting to microaggressions in medicine and surgery. *JAMA Surg*. 2019;154(9):868-872. https://doi.org/10.1001/jamasurg.2019.1648
7. Jones CP. Levels of racism: a theoretic framework and a gardener's tale. *Am J Public Health*. 2000;90(8):1212-1215. https://doi.org/10.2105/AJPH.90.8.1212
8. Sotto-Santiago S, Mac J, Duncan F, Smith J. "I didn't know what to say": responding to racism, discrimination, and microaggressions with the OWTFD approach. *MedEdPORTAL*. 2020;16:10971. https://doi.org/10.15766/mep_2374-8265.10971
9. Nasca TJ. A message from Dr. Thomas J. Nasca. Accreditation Council for Graduate Medical Education. June 5, 2020. Accessed September 23, 2024. https://www.acgme.org/newsroom/2020/6/a-message-from-dr.-thomas-j.-nasca
10. Meunier J, Merckaert I, Libert Y, et al. The effect of communication skills training on residents' physiological arousal in a breaking bad news simulated task. *Patient Educ Couns*. 2013;93(1):40-47. https://doi.org/10.1016/j.pec.2013.04.020
11. McKillip KM, Moss F. Power, silence, and debriefing: hidden harms when palliative teams encounter racism. *J Pain Symptom Manage*. 2021;61(6):1311-1315. https://doi.org/10.1016/j.jpainsymman.2021.01.128

Copyright © 2024 Gibbons et al. This is an open-access publication distributed under the terms of the Creative Commons Attribution license.

12. Jowell AR, James AK, Jasrasaria R, et al. DARE training: teaching educators how to revise internal medicine residency lectures by using an anti-racism framework. *MedEdPORTAL*. 2023;19:11351. https://doi.org/10.15766/mep_2374-8265.11351

13. Akuffo-Addo E, Dalson J, Agyei K, et al. Barriers to Black medical students and residents pursuing and completing surgical residency in Canada: a qualitative analysis. *J Am Coll Surg*. 2024;239(2):151-160. https://doi.org/10.1097/XCS.0000000000001067

14. Yuce TK, Turner PL, Glass C, et al. National evaluation of racial/ethnic discrimination in US surgical residency programs. *JAMA Surg*. 2020;155(6):526-528. https://doi.org/10.1001/jamasurg.2020.0260

15. Souza T. Responding to microaggressions in the classroom: taking ACTION. Faculty Focus. April 30, 2018. Accessed September 23, 2024. https://www.facultyfocus.com/articles/effective-classroom-management/responding-to-microaggressions-in-the-classroom/

16. OTFD—a powerful communication technique. Prezi. Updated November 13, 2013. Accessed September 23, 2024. https://prezi.com/tszt1slnknpa/otfd-a-powerful-communication-technique/

17. Ganote C, Souza T, Cheung F. Pedagogies of microresistance for equity and social justice. In: Kumar R, Refaei B, eds. *Equity and Inclusion in Higher Education: Strategies for Teaching*. University of Cincinnati Press; 2021:71-82.

18. Souza T. Managing hot moments in the classroom: concrete strategies for cooling down tension. In: *Diversity and Inclusion in the College Classroom*. Magna Publications; 2016:4-5.

19. DallaPiazza M, Padilla-Register M, Dwarakanath M, Obamedo E, Hill J, Soto-Greene ML. Exploring racism and health: an intensive interactive session for medical students. *MedEdPORTAL*. 2018; 14:10783. https://doi.org/10.15766/mep_2374-8265.10783

20. Neves da Silva HV, Heery LM, Cohen WR, et al. What happened and why: responding to racism, discrimination, and microaggressions in the clinical learning environment. *MedEdPORTAL*. 2022;18:11280. https://doi.org/10.15766/mep_2374-8265.11280

21. Felix HM. Conceptual frameworks in medical simulation. StatPearls. Updated September 26, 2022. Accessed September 23, 2024. https://www.statpearls.com/point-of-care/63798

22. Wijnen-Meijer M, Brandhuber T, Schneider A, Berberat PO. Implementing Kolb's experiential learning cycle by linking real experience, case-based discussion and simulation. *J Med Educ Curric Dev*. 2022;9:23821205221091511. https://doi.org/10.1177/23821205221091511

23. Calardo SJ, Kou M, Port C, et al. Realizing Inclusion and Systemic Equity in Medicine: Upstanding in the Medical Workplace (RISE UP)—an antibias curriculum. *MedEdPORTAL*. 2022;18:11233. https://doi.org/10.15766/mep_2374-8265.11233

24. Cheng S. Stop, talk, roll: how to deal with tough communication exchanges in the medical workplace. Georgetown University School of Medicine. May 10, 2017. Accessed September 23, 2024. https://som.georgetown.edu/diversityandinclusion/studentorganizations/stoptalkroll/

25. Mastering tough conversations. VitalTalk. Accessed September 23, 2024. https://chooseyourpath.vitaltalk.org/mastering-tough-conversations/

26. Eppich W, Cheng A. Promoting Excellence and Reflective Learning in Simulation (PEARLS): development and rationale for a blended approach to health care simulation debriefing. *Simul Healthc*. 2015;10(2):106-115. https://doi.org/10.1097/SIH.0000000000000072

27. Kern DE. Overview: a six-step approach to curriculum development. In: Thomas PA, Kern DE, Hughes MT, Tackett SA, Chen BY, eds. *Curriculum Development for Medical Education: A Six-Step Approach*. 4th ed. Johns Hopkins University Press; 2022:8-28.

28. Lovejoy C. The benefits of reflection and role models in learning medicine: an interview with Dr Paquita De Zulueta. *Camb Med J*. 2018;9:001.

29. Ackerman-Barger K, Jacobs NN, Orozco R, London M. Addressing microaggressions in academic health: a workshop for inclusive excellence. *MedEdPORTAL*. 2021;17:11103. https://doi.org/10.15766/mep_2374-8265.11103

30. White-Davis T, Edgoose J, Brown Speights JS, et al. Addressing racism in medical education: an interactive training module. *Fam Med*. 2018;50(5):364-368. https://doi.org/10.22454/FamMed.2018.875510

31. March C, Walker LW, Toto RL, Choi S, Reis EC, Dewar S. Experiential communications curriculum to improve resident preparedness when responding to discriminatory comments in the workplace. *J Grad Med Educ*. 2018;10(3):306-310. https://doi.org/10.4300/JGME-D-17-00913.1

Received: December 28, 2023
Accepted: June 6, 2024
Published: October 24, 2024

Copyright © 2024 Gibbons et al. This is an open-access publication distributed under the terms of the Creative Commons Attribution license.
